# Raman spectroscopy reveals oxidative stress-induced metabolic vulnerabilities in early-stage AR-negative prostate-cancer versus normal-prostate cell lines

**DOI:** 10.1038/s41598-024-70338-1

**Published:** 2024-10-25

**Authors:** M. Cameron, F. Frame, N. J. Maitland, Y. Hancock

**Affiliations:** 1 School of Physics, Engineering and Technology, University of York, Heslington, York, YO10 5DD UK; 2 Department of Biology, University of York, Heslington, York, YO10 5DD UK; 3 York Biomedical Research Institute, University of York, Heslington, York, YO10 5DD UK

**Keywords:** Biophysics, Cell biology, Prostate cancer, Raman spectroscopy

## Abstract

Quantitative Raman spectroscopy provides information-rich imaging of complex tissues. To illustrate its ability to characterise early-stage disease, we compared live P4E6, a low-grade Gleason-3 prostate-cancer cell line, to PNT2-C2, a normal prostate cell-line equivalent, thereby elucidating key molecular and mechanistic differences. Spectral changes from statistically relevant population sampling show P4E6 is defined by reduced DNA/RNA signatures (primarily base-pair modifications), increased protein-related signatures (synthesis), decreased whole-cell measured saturated and unsaturated fatty acids, and increased cholesterol and cholesterol ester (lipid storage). Signatures in the live-cell disease state point to the Warburg effect for aerobic glycolysis as the mechanism for cellular energy generation. A follow-on study involving catastrophic desiccation showed a key survival pathway in the cancer state in the structural robustness of DNA/RNA. Metabolic changes, namely in Warburg-to-oxidative-phosphorylation rerouting and reduced protein synthesis, were also shown. Such modifications limit cancer’s resistance to oxidative damage, and thus its ability to utilise a higher redox homeostasis for metabolic advantage. The results demonstrate the ability of quantitative Raman spectroscopy to uncover, with full molecular-heterogeneity capture, mechanistic vulnerabilities in lowest-grade tumorigenic prostate cancer, thereby revealing underlying targets for disease disruption at early stage.

## Introduction

Prostate cancer is the fourth most commonly diagnosed cancer across all genders and second most common cancer in males, with 1.4M new cases in 2020 worldwide^1^. Molecular characterisation of the disease, and determining the underpinning molecular-level mechanisms for its initiation and progression, are challenging due to its complexity and heterogeneity both within and between patients, at the single-cell, and inter- and intra-tumoral levels, and in the metastatic state. Genomic studies of prostate cancer show stratification to subtypes well beyond clinical measures of Gleason score, lymph-node invasion, and tumour grade^2^. In transcriptomic studies, the disease state exhibits complex mechanistic pathways and spatial heterogeneity in its classification and progression^3^. Length-scale complexities are also apparent, whereby genomic and transcriptomic aberrations do not to correlate to disease-related changes at other molecular length scales, such as in proteins and lipids^4,5^. As such, there are unresolved issues in combining across separate genomic, lipidomic and proteomic studies that involve destructive testing, as well as testing under different environmental conditions.

To assist with these limitations and challenges, Raman spectroscopy is proposed as a complementary approach for label-free, molecular-scale characterisation of intact specimens. Raman spectroscopy molecularly fingerprints samples via non-destructive, laser light (photon-molecule-scattering) interaction—the so-called Raman effect^6^. Raman spectroscopy has potential for biomedical applications in diagnostics and in treatment studies across a broad range of diseases and system types^7^. Specific to prostate cancer is its use to characterise the disease state of cellular components (e.g., exosomes)^8^, single cells^9^, and tissue samples^10,11^. Raman spectroscopy has also been used to elucidate mechanisms for treatment action^12^, as well as to monitor treatment response, such as in lipid droplets in prostate-cancer cells^13,14^. Pertinent to the study of molecular heterogeneity, which is of particular importance in cancer, is its ability to obtain spatially resolved molecular information down to $\sim 1\,\hbox {micron}$ sampling^15^.

This work uses Raman spectroscopy to molecularly characterise P4E6, a low-grade tumorigenic prostate-cancer cell line^16^, against the PNT2-C2 normal cell-line equivalent^16–18^. P4E6 is a Gleason-3 AR-negative cancer—a model system for an early-stage, difficult-to-treat prostate cancer^19^. To the best of our knowledge, P4E6 has not been Raman characterised before, with most studies focussed on metastatic cell lines^9,10,13,14,20,21^. In the first part of the Results, we perform Raman spectroscopy to distil robust biomarkers that determine the live P4E6 disease state by its direct comparison against the live PNT2-C2 normal cell-line results. In the second part of the Results, we go beyond routine Raman characterisation of the two cell lines in the live state to also define and elucidate mechanistic strengths and weaknesses in sustaining cancer through a catastrophic state change by extreme desiccation (air-drying). To quantitatively define this state change, biomarkers in the P4E6 dried-cell disease state are defined relative to the PNT2-C2 dried-cell normal results. These dried-state disease biomarkers are then compared to the live-state disease biomarkers to then assess across proteomic, lipidomic and genomic differences relating to mechanistic changes.

Mammalian cells have a complex response to desiccation leading to stress-induced gene-level modifications and transcription changes^22^. Other deleterious modifications include protein-structure shifts and induced disorder from water removal, as well as irreversible breakdown of the cellular lipid bilayer^23^. Raman studies of dried cancer cells have shown marked changes in DNA/RNA and proteins, such as structural breakdown of the DNA backbone and bases, as well as decrease in protein stability^24,25^. A further study on *extracted* molecular components from Calu-1 lung-cancer live and dried cells showed a relative increase in RNA-related bands and decrease in protein-related bands^26^. Hence, we expect to find specific molecular differences by comparing the live versus dried P4E6 cancer cells and PNT2-C2 normal equivalent that will then enable a mechanistic interpretation of the disease.

Cellular desiccation has been correlated to mechanistic markers relating to oxidative stress, such as an increase in free radicals in the form of reactive oxygen species (ROS), which if left unchecked, will attack DNA, proteins, and lipids^27^. Raman-spectroscopy studies have typically involved specific molecular components on the oxidative effects in lipids^28^ and oxidative stress in various nucleic acid components in DNA^29^. Few works have investigated oxidative stress in mammalian cells using Raman spectroscopy—e.g., Ref. ^30^, which studies chemically induced oxidative stress and the effects of anti-oxidant treatment in normal colon cells. Oxidative stress is a known factor in the molecular initiation and progression of prostate-cancer disease^31^. In healthy cells, redox homeostasis reinstates normal cell-state ROS levels, whereas a remodelling of redox homeostasis in cancer cells occurs by additional antioxidant mechanisms that adapt to and maintain an increased ROS state^32^. By forcing the P4E6 cancer state to undergo extreme oxidative stress, we show it metabolically transitions from one of advantage to disadvantage over the normal-state equivalent. Quantitative revelation of such processes within a model-system construct enables these pathways to be accessible for later assessment and testing in the more heterogeneous patient system, thereby allowing key mechanistic properties in difficult-to-treat AR-negative prostate cancer to be identified and disrupted at the earliest possible stage.

## Results

### Live-cell Raman molecularly characterises the P4E6 prostate-cancer cell line against the PNT2-C2 normal cell line equivalent

**Fig. 1:**
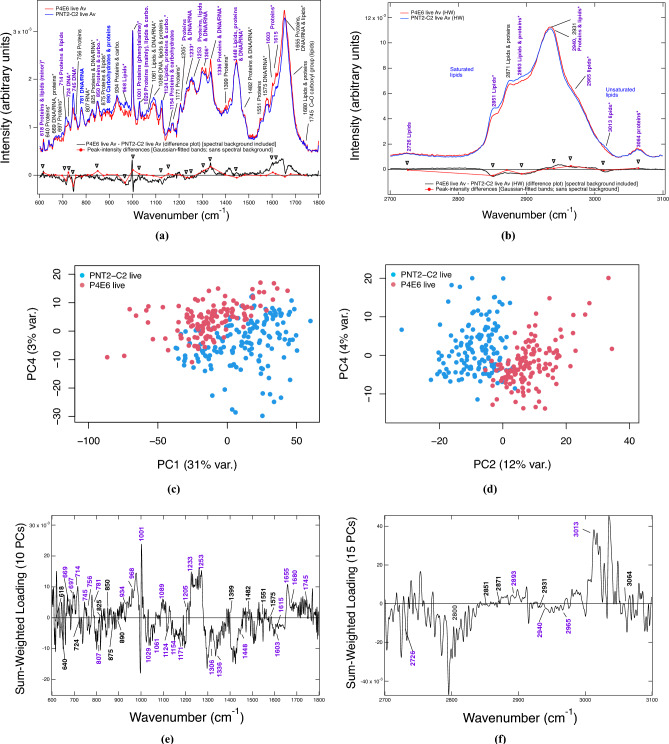
Live-cell P4E6 [${\text {N}}_{\text {s}}=137$ fingerprint (FP) and 136 high-wavenumber (HW) spectra], and PNT2-C2 [$\hbox {N}_{{\textrm{s}}}=151$ (FP) and 152 (HW) spectra]. End-to-end baselined and total-area-normalised spectral averages, spectral difference plot (black) and Gaussian-fitted peak-intensity difference plot (red): **(a)** FP region and **(b)** HW region (cf. Tables S1–S3). Representative PCA scatter plots with maximum variance and separation captured: **(c)** FP region and **(d)** HW region. Sum-weighted loadings over the minimum number of principal components (PCs) required for stable leave-one-out cross-validation (LOOV) uncertainty: **(e)** FP region (60% variance captured) and **(f)** HW region (79% variance captured) (cf. Table S4, Figs. S7, S8). Discriminatory bands found in the peak-fitted difference plots in (a), (b) that are $\gg$ the sum of the fitted SE uncertainties at 1–6% SE per fitted band, or are above background in the sum-weighted loadings in (e) and (f), are in purple. Those bands approximate to the sum of the fitted SE uncertainties in the peak-intensity plots are of secondary significance and are in blue. Bands that are labelled black in (a), (b), (e), or (f) are insignificant, i.e., $\sim 0$ peak-intensity difference and/or sum-weighted loadings. Bands later determined as markers for drying effects are also labelled *. Cf. Table 1, S5, S6 and Figs. S9, S10, S17.

Live-cell Raman spectroscopy was performed on P4E6 and PNT2-C2 cell lines (Fig. S1) and then quantitatively analysed to molecularly define the disease state. Figures 1(a) and (b) show the averaged total-area-normalised spectra obtained in the fingerprint (600–$1800\,\hbox {cm}^{-1}$) and high-wavenumber (2700–$3100\,\hbox {cm}^{-1}$) regions, together with the calculated spectral difference, inclusive of the background. The spectral averages were obtained from statistically converged sets of Raman data (Figs. S2, S3). Peak assignment labels to the fitted bands were acquired from various sources (Tables S1, S2). The fitted peak-intensity differences between the cancer and normal states were also calculated and plotted (Figs. 1(a), (b), Table S3).

PCA shows good separation of the P4E6 and PNT2-C2 classes [Figs. 1(c), (d)], with a LDA-leave-one-out cross-validation (LOOV) prediction accuracy of $\sim$98% converged at 10 PCs for the fingerprint region and 15 PCs for the high-wavenumber region (Table S4, Figs. S7, S8). Molecular classification of the disease state was assisted using a PCA-LDA calculation of a weighted-summation loadings plot, which accounts for the LDA weights associated with those PCs included in the LOOV classification result [Figs. 1(e), (f)]. For robust assignment of molecular features that discriminate comparative states, the peak-intensity subtractions [Figs. 1(a), (b)] were required to be outside of the sum of the fitted SE uncertainties determined as ± 1–6% SE per fitted band, as well as be apparent at these fitted wavenumber positions above noise in the weighted-loadings (Figs. 1(e), (f), also Tables S5, S6). The distilled results from these comparisons (Table 1) shows the main class-separating bands between the P4E6 cancer and PNT2-C2 normal-equivalent cell lines, thereby defining the P4E6 disease state.

**Table 1:**
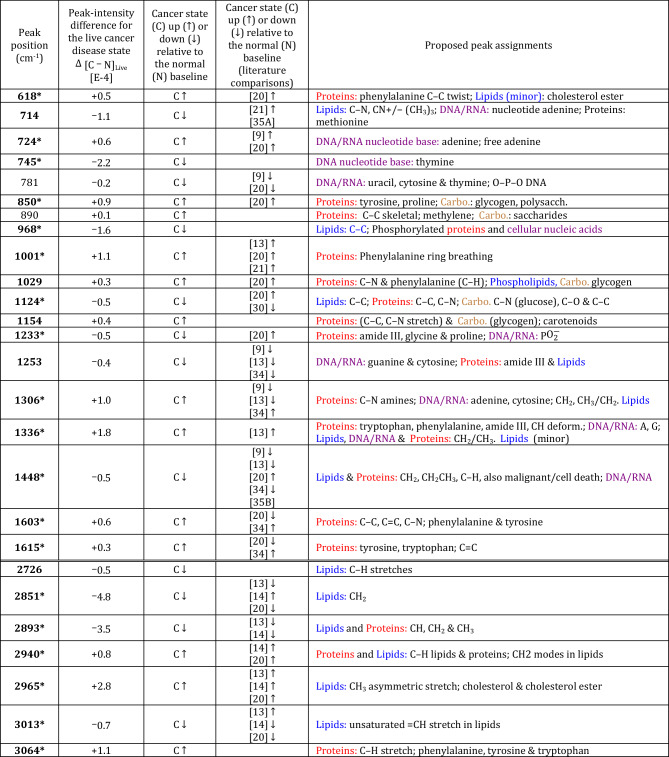
Class-separating peak assignments for P4E6 live vs. PNT2-C2 live distilled from the average spectral subtractions and PCA-LDA weighted loadings (cf. Figs. 1(a), (b), (e), and (f), Tables S3, S5, S6). **Column 1:** Class-separating bands. Those bands with $\Delta$ $>>$ the sum of the fitted SE uncertainties (1–6% SE per fitted band) are in **bold**, whereas those bands with $\Delta$ > the sum of the SE-fitted peak uncertainties are in plain text. Bands that will later also be markers for drying effects are highlighted as *. **Column 2:** Quantitative differences $\Delta = [\hbox {C}{-}\hbox {N}]_{\text {Live}} =$ [P4E6 minus PNT2-C2]$_{\text {Live}}$ in the Gaussian-fitted peak-intensity values. The cancer disease state is defined relative to the normal baseline. **Column 3:** Relative change in P4E6 cancer (C) against the normal (N) PNT2-C2 baseline where $\uparrow$ = up-regulated in cancer and $\downarrow$ = down-regulated in cancer. **Column 4:** Closest literature comparisons for cancer up-regulation and down-regulation with corresponding references at the bottom of the table showing the systems studied (cf. Table S13). Where a relative band increase ($\uparrow$) or decrease ($\downarrow$) has been indicated in the literature, then this information has also been added. **Column 5:** molecular peak assignments (cf. Table S2). Colours are used to enable quick reference to the underpinning macromolecules (Proteins, Lipids, DNA/RNA and Carbohydrates).

[9] LNCaP, PCa 2b (AR-positive) and PC3, DU145 (AR-negative) metastatic cell lines, with focus on the relative trends in AR-negative cell lines. Cells kept “moist”; [13] LNCaP and PC3 metastatic cells lines. Untreated versus treated. Formaldehyde fixed. PC3 shown; [14] PC3 and LNCaP versus PNT2 normal prostate cell line. Cells fixed with paraformaldehyde. PC3 shown; [20] DU145 vs. PNT2-C2 metastatic prostate cancer vs. normal equivalent. Ethanol fixed; [21] LNCaP (metastatic prostate cancer) vs. PNT1A (normal) cell lines. Ethanol fixed; [34] Prostate tissues from biopsy. Benign, Gleason 3, 4 and 5 comparisons. Formalin fixed; [35A] DNA fibres in $\hbox {H}_{\textrm{2}}\hbox {O}$. A-form DNA, and [35B] B-form DNA.

The P4E6 disease state exhibits relative band increases in protein signatures, which account for phenylalanine, tyrosine, and tryptophan (e.g., at 618, 1001, 1029, 1336, 1603, 1615, and $3064\, \hbox {cm}^{-1}$), as well as in other protein-dominant bands (e.g., at 850, 890, 1154, 1306, and $2940\,\hbox {cm}^{-1}$). However, bands that typically comprise convolved proteins, lipids, and DNA/RNA are down-regulated against the PNT2-C2 normal state (e.g., at 714, 968, 1233, 1253, 1448, and $2893\,\hbox {cm}^{-1}$) (Table 1). The relative decreases in the amide III component of the $1233\,\hbox {cm}^{-1}$ and $1253\, \hbox {cm}^{-1}$ bands represent key $\alpha$-helix changes as per Ref. ^33^, with these also reported as disordered protein modifications in the AR-negative DU145 metastatic cell line against other prostate-cancer types^9^. Ref. ^20^ conjectured that molecular characteristics of androgen-negative cell lines have increased protein bands (phenylalanine, tyrosine, and, in part, amide III), plus relative increases in DNA based on their Raman study of an ethanol-fixed DU145 cell line compared to the normal cell line PNT2. However, these same trends were also reported for the androgen-positive metastatic cell line LNCaP relative to the normal cell line PNT1A (ethanol fixed)^21^. In general, we believe Raman shows relative protein-band increases that are indicative of the prostate-cancer state irrespective of AR status.

The synthesis and use of amino acids is a key mechanism in prostate-cancer cell growth and survival, with these cells requiring more energy to function than normal-prostate cells^36,37^. Protein synthesis and its related metabolic routes are evidenced in Table 1, with the up-regulation of protein-specific Raman markers in the P4E6 disease state indicative of protein-synthesis pathways associated with phenylalanine, tyrosine, tryptophan, and nitrogen-based amines. Increase in these proteins occur via metabolism-to-synthesis mechanisms (e.g., phenyalanine to tyrosine synthesis) and/or via breakdown of more complex proteins to form amino acids. The metabolism of these proteins supports the cancer state by sustaining energy requirements and growth factors^36^. For example, amino-acid synthesis and metabolism are directly linked to glucose metabolism^38^.

Changes in DNA/RNA provide further discriminatory information in stratifying prostate-cancer cell lines. Relative band increases associated with the A-form of DNA and band decreases associated with the B-form of DNA were reported for the metastatic cell lines DU145^20^ and LNCaP^21^, which have different AR status. Ref. ^9^ also showed DU145 has a non-discriminatory overall decrease in DNA/RNA against other metastatic prostate-cancer cell lines. Therefore, although DNA/RNA changes are apparent, there is no consensus that conformational differences are related to AR status. In P4E6, there are no specific conformal changes associated with DNA/RNA relative to PNT2-C2. The band at $714 \,\hbox {cm}^{-1}$ attributed in part to A-DNA, and the band at $1448 \,\hbox {cm}^{-1}$ that corresponds in part to the B-form of DNA, are both reduced in P4E6 relative to PNT2-C2 (Table 1)^35^. Again, convolution plays a key role in challenging discernment. Table 1 shows that P4E6 has a relative decrease in the DNA/RNA-specific bands at $745\, \hbox {cm}^{-1}$ related to thymine, and $781\,\hbox {cm}^{-1}$ related to the U, C, and T bases and O–P–O stretch. The results indicate structural modifications to the base pairs as a key P4E6 cancer-state distinction—suspected due to base-pair mismatches and methylation^39^, as well as modifications to the phosphate backbone, supported also in addition by the down-regulation in P4E6 in the $\hbox {PO}^{-}_{2}$ component of the $1233 \,\hbox {cm}^{-1}$ marker. An exception is adenine at $724 \,\hbox {cm}^{-1}$, which is up-regulated in the P4E6 cancer state and hence possibly related to increased adenine nucleotide as free “unused” adenine or as insertions (i.e., adenene richness) as evidenced by other studies in prostate cancer^40^ and other cancers^41^.

Other key considerations in defining the prostate-cancer disease state are lipid accumulation and lipid metabolism indicated by relative changes in the unsaturated and saturated fatty-acid content in the cells, along with cholesterol-ester accumulation^13,14,55^. P4E6 shows in its lipid indicators, higher relative cholesterol and cholesterol ester ($2965\, \hbox {cm}^{-1}$), lower-relative saturated fatty-acids (at $968\, \hbox {cm}^{-1}$—absent in P4E6, and at 1448, 2851, and $2893\, \hbox {cm}^{-1}$), and lower-relative unsaturated fatty-acids ($3013\, \hbox {cm}^{-1}$) compared to the PNT2-C2 normal-state—bearing in mind that some of these markers are also convolved with other macromolecules, such as protein signatures (Table 1). Increased cellular cholesterol and cholesterol-ester conversion are beneficial in AR-independent prostate-cancer growth^43^. The results also show a larger relative decrease in P4E6 in the whole cell–measured saturated fatty-acid bands compared to the unsaturated fatty-acid bands relative to the normal state. The peak intensity ratio for the total unsaturated fatty-acid to total fatty-acid content (TUFA/TFA^13^) in the disease state is $3013{/}2851= 0.14 \pm 0.01$.

A relative decrease in the total saturated-to-unsaturated fatty-acid content as measured by Raman, as well as an increase in fatty-acid storage, in general, have been proposed as indicators of prostate-cancer cells^13^. The relative decrease in both overall saturated and unsaturated lipid-content in P4E6 may be representative of higher lipid metabolism in prostate cancer. Alternatively, this change may also be an artefact of the full-nucleus Raman sampling, and hence preference in prostate-cancer cells for increased lipid-droplet storage against free cellular availability. For example, Ref.^14^ showed that a key saturated lipid band relating to $\hbox {CH}_2$ at $2851\, \hbox {cm}^{-1}$ can appear to have a relative reduced intensity when averaged over the entire cell nucleus. Their work also showed a relative increase in cholesterol esters (unsaturated fatty acids) compared to triacylglycerols (saturated fatty acids) in the PC3 AR-negative metastatic cell line compared to PNT2. This result is supported in the P4E6 finding by the relative increase in the $2965\, \hbox {cm}^{-1}$ band associated with cholesterol and cholesterol ester being indicative of the prostate-cancer state (see also Ref. ^44^). It is also commensurate with Ref. ^43^, which showed an increase in free-cholesterol availability and cholesterol-ester lipid-droplet storage in prostate-cancer cells through their lipidomic study of the PC3 cell line.

In summary, the live-cell P4E6 prostate-cancer state has been characterised for the first time by Raman spectroscopy. P4E6 is defined by reduced signatures in DNA/RNA implying base-pair mismatches and/or methylation (the exception being adenine), an increase in protein-related signatures relating to synthesis, an increase in cholesterol and cholesterol-ester indicators, and a relative decrease in whole cell–measured saturated and unsaturated fatty acids (beneficial lipid metabolism). Such changes against the normal state defined by PNT2-C2 define key mechanisms by which low-grade tumorigenic prostate cancer at the single-cell level, which is AR-negative, can be fortified and sustained. The combination of these metabolic features, *together* with relative band increases related in part to (poly)saccharides and glycogen (850, 890, 1029 and $1154 \,\hbox {cm}^{-1}$) versus reduction in phosphorylated proteins and nucleic acids ($968 \,\hbox {cm}^{-1}$) and increase in adenine ($724 \,\hbox {cm}^{-1}$), point to the Warburg effect^36,45,46^, as opposed to oxidative phosphorylation (i.e., NADH production and use), as being active in sustaining the live P4E6 prostate-cancer state.

**Fig. 2:**
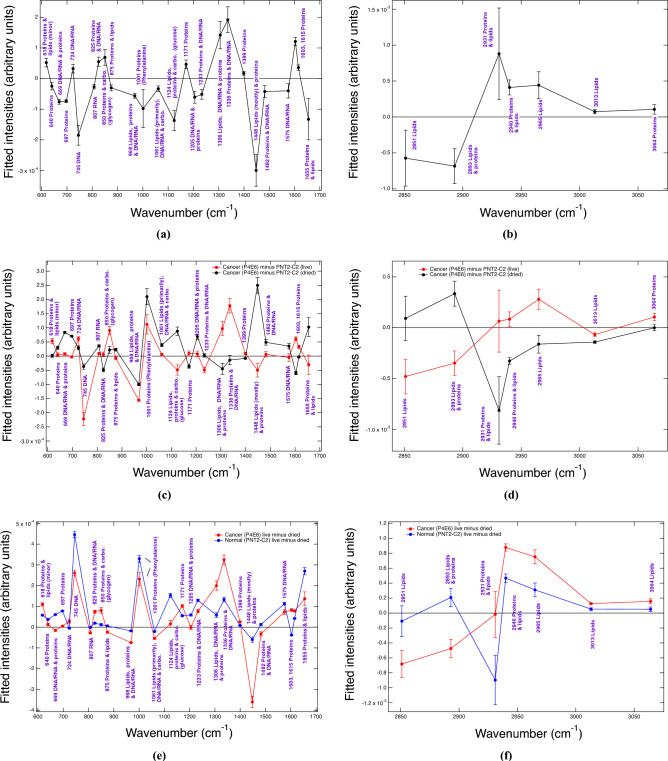
Gaussian peak-fitted difference plots obtained from the normalised spectral averages for live P4E6 [fingerprint (FP): $\hbox {N}_{\text {s}}=137$; high wavenumber (HW): $\hbox {N}_{\text {s}}=136$], live PNT2-C2 [FP: $\hbox {N}_{\text {s}}=151$; HW: $\hbox {N}_{\text {s}}=152$], dried P4E6 [FP: $\hbox {N}_{\text {s}}=112$; HW: $\hbox {N}_{\text {s}}=112$], and dried PNT2-C2 [FP: $\hbox {N}_{\text {s}}=154$; HW: $\hbox {N}_{\text {s}}=154$] cell lines (see also Fig. 18 of the SI for the corresponding spectral averages). $\hbox {N}_{\text {s}}$ is the number of spectra in each statistically converged dataset. **(a)**, **(b)** FP and HW live-minus-dried *disease-state* difference plots, where the live and dried disease states are defined relative to their respective normal-equivalent baselines. **(c)**, **(d)** FP and HW, P4E6 minus PNT2-C2 live and dried disease-state plots. **(e)**, **(f)** FP and HW P4E6 cancer and PNT2-C2 live-minus-dried plots. Cf. Tables 2 and 3, also SI Table S3. The uncertainties in these figures are taken from the sum of the peak-fitted SE uncertainties at $\sim 1$–6% SE per fitted band.

### Extreme cellular-desiccation stress reveals key mechanistic strengths and weaknesses in P4E6 cancer pathways

A comparative dried-cell analysis was performed to assess the mechanistic resilience of the P4E6 cancer state. Direct comparison of the Gaussian-fitted peak-intensity difference plots shows cancer-specific changes caused by drying effects via biomarkers that molecularly discriminate the P4E6 cancer disease state relative to the PNT2-C2 normal state (Fig. 2). PCA-LDA classification for the P4E6 and PNT2-C2 dried-cell comparisons gives excellent class separation of 100% in the fingerprint region with 54% of the variance captured (10 PCs), and 99.6% in the high-wavenumber region with 77% of the variance captured (7 PCs). For the PCA and PCA-LDA results, plus full sets of the quantitative differences across all measures, see Table S4, the tables in the SI Sections E–K, and the Figs. in SI Sections D–J.

The results extracted from the full set of quantitative comparisons—together with the closest equivalent, literature comparisons, which show favourable comparisons—are in Tables 2 and 3. The most significant differences caused by drying effects (i.e., with $\vert \delta \vert \ge 0.5$ magnitude difference in the live relative to the dried disease state outside of the combined SE peak-fitted uncertainties) are at 618, 669*, 697*, 745, 825*, 850, 968, 1001, 1124, 1171*, 1205*, 1233, 1306, 1336, 1448, 1603, 1655*, 2851, 2893, 2931*, 2940, 2965, 3013, and $3064\,\hbox {cm}^{-1}$. Those biomarkers with * are new, outside of those previously determined for the live disease-state comparison (cf. Table 1).

The most substantial effects due to catastrophic drying in DNA/RNA occur in base changes at 669, 724, 745, 1205, 1306, 1336, 1482, 1575, and $1655 \,\hbox {cm}^{-1}$, with secondary effects in the O–P–O backbone at 807, 825, 1061, and $1233 \,\hbox {cm}^{-1}$ (Table 2). The predominance in DNA/RNA base changes is supported by other literature evidence (e.g., Refs. ^47,48^, plus references therein). Underpinning most of these relative differences, the cancer disease state is more resilient against desiccation-induced DNA/RNA modifications with $\vert \Delta _{\text {C}}\downarrow \vert <\vert \Delta _{\text {N}}\downarrow \vert$, else $\vert \Delta _{\text {C}}\vert \sim 0$ in DNA/RNA-dominant bands (Table 2). There are two notable exceptions to this trend. The first exception is at $724 \,\hbox {cm}^{-1}$ relating to adenine, which is likely due to cancer’s increase in adenine via base insertion, or lack of free adenine use in cellular energy production in the live state (noted also in the live-cell study). The second exception is at $807 \,\hbox {cm}^{-1}$, which is an emergent difference relating to the RNA O–P–O stretch. The $745\, \hbox {cm}^{-1}$ band related to thymine, which was particularly implicated in DNA base-molecule modifications in the live-cell cancer disease state ($-2.2$ C$\downarrow$ in Table 1), is again dominant in the dried-cell study ($\delta =1.8\downarrow$ in the live versus dried disease state).

**Table 2:**
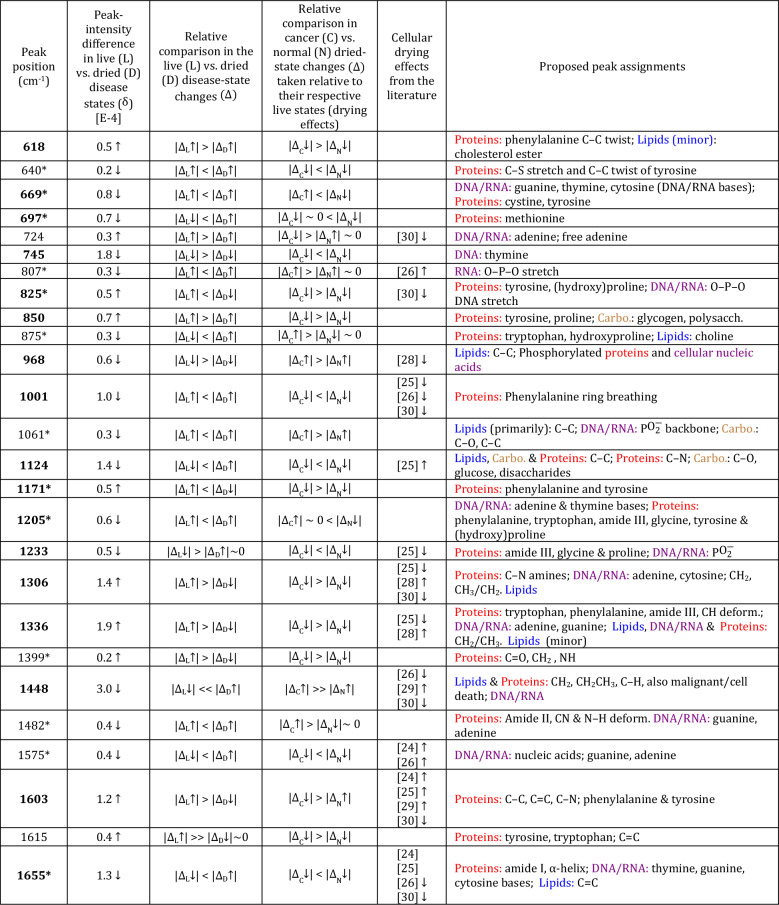
Peak assignments that discriminate drying effects for P4E6 cancer (C) versus the PNT2-C2 normal (N) states (fingerprint region). Biomarkers that are statistically significant outside of the sum of the standard errors for the fitted peak intensities at 1-6% SE uncertainty per fitted band are in: **bold** $>>$ SE, and plain text > SE. Those with * are emergent due to cellular desiccation. **Column 1:** resolved band positions. **Column 2:** Fitted peak-intensity differences ($\delta$) between the live (L) and dried (D) cancer disease states defined relative to their respective normal states (i.e., [P4E6 minus PNT2-C2]$_{\text {Live}}$ minus [P4E6 minus PNT2-C2]$_{\text {Dried}}$). The arrow indicates positive ($\uparrow$) or negative ($\downarrow$) change relative to the dried disease-state baseline [cf. Fig. 2(a)]. **Column 3:** Magnitude comparisons for live and dried disease-state changes ($\uparrow$ or $\downarrow$) in cancer (C) relative to their respective normal (N) baselines, i.e., $\Delta _{\text {L}}$ = [P4E6 minus PNT2-C2]$_{\text {Live}}$ and $\Delta _{\text {D}}$ = [P4E6 minus PNT2-C2]$_{\text {Dried}}$. **Column 4:** Magnitude comparisons for dried-state changes ($\uparrow$ or $\downarrow$) in cancer (C) and normal (N) relative to their respective live states (i.e., relating to drying effects), i.e., $\Delta _{\text {C}}$ = [P4E6$_{\text {Dried}}$ minus P4E6$_{\text {Live}}$] and $\Delta _\text {N}$ = [PNT2-C2$_{\text {Dried}}$ minus PNT2-C2$_{\text {Live}}$]. **Column 5:** Closest literature comparisons for dried-state changes (cf. with either $\Delta _{\text {C}}$ or $\Delta _{\text {N}}$ in Column 4). Where a relative band increase ($\uparrow$) or decrease ($\downarrow$) has been indicated in the literature, then this information has also been added. Specific systems from these references are listed as footnotes below (cf. Tables S14, S15). **Column 6:** Proposed peak assignments. Colours are used for quick reference to the underpinning macromolecules (Proteins, Lipids, DNA/RNA and Carbohydrates). See also Fig. 2 and SI Table S3 for the detailed quantitative values associated with the qualitative trends summarised in this table.

[24] MLE-12 mouse lung cells (live vs. dead); [25] A549 human lung adenocarcinoma cells. Live cells vs. cells that are dead in culture via in situ Raman; [26] Calu-1 human non-small-cell lung cancer (live vs. air dried). RNA and protein differences only (extracted molecules); [28] Linoleic acid—oxidation damage by time-dependent heating; [29] Chemically-oxidised purine bases (nucleotides); [30] CCD-18Co normal colon cell line (paraformaldehyde fixed) with oxidative damage via *t*BHP addition—surface-enhanced Raman spectroscopy (SERS).

**Table 3:**
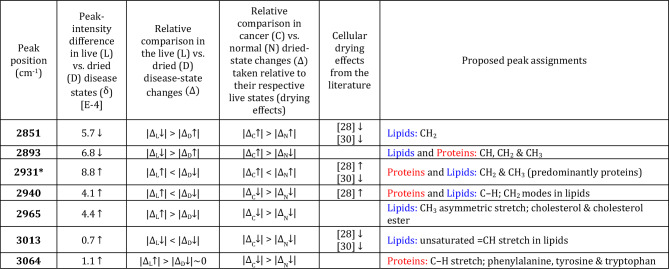
Peak assignments that discriminate drying effects for P4E6 cancer (C) versus the PNT2-C2 normal (N) states (fingerprint region). Biomarkers that are statistically significant outside of the sum of the standard errors for the fitted peak intensities at 1-6% SE uncertainty per fitted band are in: **bold** $>>$ SE, and plain text > SE. Those with * are emergent due to cellular desiccation. **Column 1:** resolved band positions. **Column 2:** Fitted peak-intensity differences ($\delta$) between the live (L) and dried (D) cancer disease states defined relative to their respective normal states (i.e., [P4E6 minus PNT2-C2]$_{\text {Live}}$ minus [P4E6 minus PNT2-C2]$_{\text {Dried}}$). The arrow indicates positive ($\uparrow$) or negative ($\downarrow$) change relative to the dried disease-state baseline [cf. Fig. 2(a)]. **Column 3:** Magnitude comparisons for live and dried disease-state changes ($\uparrow$ or $\downarrow$) in cancer (C) relative to their respective normal (N) baselines, i.e., $\Delta _{\text {L}}$ = [P4E6 minus PNT2-C2]$_{\text {Live}}$ and $\Delta _{\text {D}}$ = [P4E6 minus PNT2-C2]$_{\text {Dried}}$. **Column 4:** Magnitude comparisons for dried-state changes ($\uparrow$ or $\downarrow$) in cancer (C) and normal (N) relative to their respective live states (i.e., relating to drying effects), i.e., $\Delta _{\text {C}}$ = [P4E6$_{\text {Dried}}$ minus P4E6$_{\text {Live}}$] and $\Delta _\text {N}$ = [PNT2-C2$_{\text {Dried}}$ minus PNT2-C2$_{\text {Live}}$]. **Column 5:** Closest literature comparisons for dried-state changes (cf. with either $\Delta _{\text {C}}$ or $\Delta _{\text {N}}$ in Column 4). Where a relative band increase ($\uparrow$) or decrease ($\downarrow$) has been indicated in the literature, then this information has also been added. Specific systems from these references are listed as footnotes below (cf. Tables S14, S15). **Column 6:** Proposed peak assignments. Colours are used for quick reference to the underpinning macromolecules (Proteins, Lipids, DNA/RNA and Carbohydrates). See also Fig. 2 and SI Table S3 for the detailed quantitative values associated with the qualitative trends summarised in this table.

[28] Linoleic acid—oxidation damage by time-dependent heating; [30] CCD-18Co normal colon cell line (paraformaldehyde fixed) with oxidative damage via *t*BHP addition—surface-enhanced Raman spectroscopy (SERS).

DNA/RNA changes in live- versus dried-cell studies have been related to structural modifications^25^, with changes to the bases linked to base-pair mismatches and/or methylation^39^. A greater relative decrease in band intensity associated with key DNA/RNA bands due to desiccation in the normal cell line PNT2-C2 indicates the healthy state is *less robust* to base-pair fragmentation and/or other chemical modifications linked to catastrophic drying than the P4E6 disease-state. Inclusive of such modifications is reactive oxygen species (ROS) attack, which would be expected due to desiccation as a result of oxidative stress. Oxidative stress is beneficial to initiating, sustaining, and proliferating the cancer disease state^49^; hence, such mechanisms to tolerate and utilise oxidative stress are already in place. For example, against the normal-state comparison, cancer has increased antioxidant activity^32^ and uses mechanistic processes, such as increased base-excision repair (BER) that directly involve the DNA/RNA bases^50,51^. In addition, ROS-induced methylation in the cancer state can lead to a more compact structure, with a higher-relative stability and increased structural rigidity^52^. We therefore conjecture these mechanistic advantages are associated with P4E6 cancer’s resilience to desiccation-induced DNA/RNA modifications. Hence, the results imply critical base-pair modifications *advantageous* to the cancer state that lead to the P4E6 DNA/RNA demonstrating greater structural robustness and resilience.

The dried-cell analysis also indicates the relative importance of protein modifications with respect to cancer- and normal-cell differences. In the live-cell comparison, up-regulation of protein synthesis and its link to metabolic purpose were implicated in P4E6 cancer (C$\uparrow$ for most of the key protein-related markers in column 2 of Table 1). In the dried-state comparison, the live disease state remains mostly up-regulated against the desiccated disease state, as evidenced by $\delta$=$\uparrow$ for protein-dominant biomarkers in column 2 of Tables 2 and 3 (e.g., at 618, 825, 850, 1171, 1306, 1336, 1399, 1603, 1615, 2931, 2940, and $3064\, \hbox {cm}^{-1}$). The change in the P4E6 cancer state due to drying is also typically greater than the change in the PNT2-C2 normal state ($\vert \Delta _{\text {C}}\downarrow \vert >\vert \Delta _{\text {N}}\downarrow(\uparrow) \vert$). Key exceptions to this trend occur in the antioxidant methionime at $697\,\hbox {cm}^{-1}$ ($\delta =0.7\downarrow$) and phenylalanine at $1001\,\hbox {cm}^{-1}$ ($\delta =1.0\downarrow$), with these having greater decreases in the normal live-state to normal dried-state transition. Another key exception occurs at 968 $\hbox {cm}^{-1}$, which relates in part to phosphorylated proteins ($\delta$ = 0.6 $\downarrow$) having up-regulation in the cancer versus normal dried state ($\vert \Delta _{\text {C}}\uparrow \vert >\vert \Delta _{\text {N}}\uparrow \vert$).

The resulting switch from favourable up-regulation of protein synthesis in the live disease state to decreased protein synthesis in the dried disease state ($\delta =\uparrow$ and $\vert \Delta _{\text {C}}\downarrow \vert >\vert \Delta _{\text {N}}\downarrow(\uparrow) \vert$) matches literature evidence suggesting a down-regulation of protein synthesis occurs due to oxidative stress^53,54^. The results show that P4E6 cancer is more affected by this mechanistic change than the normal state upon catastrophic desiccation. This shift would also explain the smaller relative decrease in phenylalanine in the cancer dried state at $1001\,\hbox {cm}^{-1}$ versus the normal dried state as being indicative of a metabolic block on phenylalanine throughput-to-synthesis (e.g., to tyrosine creation). The combination of these findings implicate metabolic switching rather than mere protein fragmentation, which could be conjectured from either a $\Delta _{\text {C}}\downarrow$ or $\Delta _{\text {N}}\downarrow$ change in isolation. The P4E6 cancer-state also increases antioxidant pathways to manage the catastrophic desiccation, for example, in maintaining methionime availability as indicated at $697\,\hbox {cm}^{-1}$. The results therefore indicate the importance of protein-related pathways against catastrophic disruption, establishing these as key mechanistic pathways that preferentially enable cancer’s cell-level survival.

Lipid differences also show marked changes in the dry-cell state (Tables 2 and 3). Cholesterol production and metabolism are vital in sustaining and proliferating the prostate-cancer disease state, for cell-death protection, and in membrane production^42,43^. A larger reduction in the cholesterol and cholesterol-ester band at $2965\,\hbox {cm}^{-1}$ in the cancer disease state (see also Fig. 2) indicates higher cholesterol synthesis and use under catastrophic desiccation via oxidative stress against the normal state. The $\hbox {CH}_2$ lipid band at $2851\,\hbox {cm}^{-1}$ shows a relative increase in P4E6 versus PNT2-C2 in the dried state as opposed to the live state ($\delta =5.7\downarrow$ in live), indicating a greater relative increase in saturated fatty acids as per $\vert \Delta _{\text {C}}\uparrow \vert >\vert \Delta _{\text {N}}\uparrow \vert$ (Table 3). We surmise this measured increase occurs due to lysis of the lipid droplets in the cells from cellular dehydration, in which case the lipid droplet content would be dispersed over the cell nuclei. This result implies lipid droplets contain more saturated fats in P4E6 than the normal state, in agreement with the relative increases in other lipid-dominant bands in the fingerprint region having $\vert \Delta _{\text {C}}\uparrow \vert >\vert \Delta _{\text {N}}\uparrow \vert$ at 968, 1061, and $1448\,\hbox {cm}^{-1}$ (Table 2) and as per other supporting literature^14,44^. The results also show increased unsaturated lipids in the live disease state as per the $3013\,\hbox {cm}^{-1}$ marker ($\delta =0.7\uparrow$ in Table 3), but with preferentially more unsaturated-lipid use under extreme cellular desiccation in the P4E6 cancer state ($\vert \Delta _{\text {C}}\downarrow \vert >\vert \Delta _{\text {N}}\downarrow \vert$). The key saturated-lipid result at $2851\,\hbox {cm}^{-1}$ and unsaturated-lipid result at $3013\,\hbox {cm}^{-1}$ are commensurate with other studies relating to cell desiccation and oxidative damage effects on lipids^28,30^.

## Discussion

The present study shows robust classification of the P4E6 cancer versus PNT2-C2 normal cell lines across univariate and multivariate methods in the live- and dried-cell states being enabled by the acquisition of Raman datasets that have demonstrated statistical convergence. To our knowledge, this is the first time that the P4E6 cell line, being representative of AR-negative low-grade (Gleason 3) tumorigenic cells, has been Raman fingerprinted and characterised. In the live-cell study, the cancer disease state (P4E6 relative to PNT2-C2) was defined by changes to the DNA/RNA bases (suspected due to base-pair modifications and/or methylation); increases in protein synthesis, carbohydrate, and cholesterol/cholesterol ester; and reductions in saturated and unsaturated lipids. These findings are commensurate with other Raman-spectroscopy studies that have characterised cell-level prostate cancer (e.g., Refs. ^9,13,14,20,21^). Together with the relative increase in (poly)saccharide and glycogen signatures, increase in free adenine (suspected not to be used in NAD+/NADPH), and reduction in phosphorylated proteins and nucleic acids, we propose the Warburg effect, a process for accelerated aerobic glycolysis^36,45^, as opposed to oxidative phosphorylation in mechanistically sustaining the live P4E6 prostate-cancer state.

The Warburg effect is beneficial to cancer cells by supporting cell proliferation and increasing ROS production^56,57^. ROS production is a function of several normal cellular functions, with metabolism being one such mechanism^32,58^. Under normal conditions, cellular homeostasis is maintained against ROS through the production and use of antioxidants to prevent oxidative stress. If left unchecked, oxidative stress opens pathways to cellular death, such as apoptosis. However, increased ROS production is beneficial to prostate cancer by protecting and proliferating the disease state, and its production is therefore heightened and sustained for this purpose, for example, by increased metabolism, which cancer exhibits^32^. Cancer has substantially higher energy demands, with metabolic pathways being one of the main differentiating factors between healthy and disease states. Such metabolic rewiring is achieved from modifications at the genomic, mRNA, and proteomic levels that determine amino-acid and lipid metabolism and glyco usage, namely via the Warburg effect^4^.

A follow-on aim was to elucidate key mechanistic pathways that cancer employs to sustain cellular viability under catastrophic attack—in this case via oxidative stress caused by extreme desiccation. A characteristic of prostate disease is increased methylation of DNA/RNA. Under oxidative stress and increased ROS attack, the DNA/RNA in prostate-cancer cells undergoes oxidative modification to the backbone and to the bases^58^. Methylation increases structural robustness, and, with increased ROS in the P4E6 disease state required mechanistically, the base pairs would have already been substantively modified. Hence, the reduction in DNA/RNA is less in the disease state relative to the normal state (Table 2). In the PNT2-C2 normal cell line, the DNA/RNA is assumed to be more susceptible to oxidative changes as its mechanism for anti-oxidant protection breaks down as a function of extreme desiccation^30^.

There is evidence of increased protein synthesis in the live P4E6 cancer state compared to the live PNT2-C2 normal state as seen in the relative increase in phenylalanine in cancer, a precursor of tyrosine, and in other amino acids involved in prostate-cancer metabolism (Table 1). This picture also fits with cancer having higher metabolic demands. Under extreme oxidative stress due to desiccation, Raman shows P4E6 to re-route this metabolic pathway as evidenced by a greater relative reduction in protein signatures, which signify reduced protein synthesis compared to the normal state, and also via metabolic blocks to key synthesis pathways (e.g., increased phenylalanine against tyrosine conversion) (Tables 2, 3). This switching in metabolic pathway acts to reduce metabolically generated ROS and instead favours increased anti-oxidant production above baseline demands—methionime being maintained in the dried cancer-state and glucose usage as another pathway for antioxidant production ($\vert \Delta _{\text {C}}\downarrow \vert <\vert \Delta _{\text {N}}\downarrow \vert$ at 697 and $1124\,\hbox {cm}^{-1}$ in Table 2). The production of NADH, an antioxidant, is another such example^58,59^. Higher NADH and hence anti-oxidant regulation under extreme desiccation occurs in the cancer state by rerouting from Warburg to oxidative phosphorylation—a necessity as the Warburg effect cannot be sustained above further increases in toxic ROS. Such re-routing is evidenced by the up-regulation of phosphorylated proteins and cellular nucleic acids at $968\,\hbox {cm}^{-1}$. The production of NADH is also indicated in the up-regulation of the $1001\, \hbox {cm}^{-1}$ band *together with* the $1575\, \hbox {cm}^{-1}$ biomarker in the dried-cell disease-state, plus equivalence of the dried-cell P4E6 and PNT2-C2 states at $1615\, \hbox {cm}^{-1}$ ($\vert -$D$\vert \sim 0$)—these being commensurate with literature evidence of key NADH markers at $1000\, \hbox {cm}^{-1}$, $1577\,\hbox {cm}^{-1}$ and $1618\,\hbox {cm}^{-1}$^60^.

Increased pooled lipids (mostly triacylglycerols and cholesterol esters) provides stored energy in cancer and a means of protecting lipids against oxidation effects^61^. Through lipid analysis, the P4E6 cancer state was shown to have higher cholesterol-ester and cholesterol bands compared to the PNT2-C2 normal state (Table 1). A relative decrease in cholesterol ester and cholesterol in the P4E6 cancer state after extreme desiccation implies increased cholesterol ester to cholesterol synthesis and immediate use of cholesterol, which has a protective effect in prostate cancer for its viability and survival^42,43,62,63^. However, through the lysis of lipid droplets, a more rapid synthesis of cholesterol ester to cholesterol, hence increased ROS production and oxidation of lipids, may have occurred leading to a deleterious effect on the cancer state^63^. Without the protective mechanism of the lipid-droplet barrier, there would be uncontrolled dispersion of free-cell lipids—also indicated in the higher, relative amount of saturated lipids in the dried-cell disease state. In this respect, extreme cellular desiccation is suspected to have a greater catastrophic effect on the prostate-cancer cell state.

In summary, Raman spectroscopy has shown sensitivity to characterise live and dried low-grade P4E6 (Gleason 3-equivalent) and PNT2-C2 normal prostate cell lines with full molecular-heterogeneity capture at the single-cell level. Relative to the normal-state baseline, P4E6 shows key mechanistic and metabolic differences that aid and sustain it in the live-cell state, including signatures of the Warburg effect (aerobic glycosis). Hence, there is evidence of redox homeostasis, specifically via a ROS steady state, even in this low-grade Gleason-3 cancer. Under catastrophic desiccation, P4E6 shows relative robustness in DNA/RNA, yet a down-regulation of protein metabolism and synthesis—the latter being a key sustaining pathway. Lysis of the lipid droplets and consequential uncontrolled release of free cellular fatty acids, including cholesterol and cholesterol ester, leads to damage to the prostate-cancer state. To counter act uncontrolled increases in ROS and oxidative damage through catastrophic desiccation, Raman shows cancer reroutes from Warburg to oxidative phosphorylation with compensatory increased production of anti-oxidants. We have used Raman spectroscopy to reveal complex mechanisms in disease-state switching in low-grade AR-negative prostate cancer, demonstrating molecular pathways for targeted arrest and disruption in difficult-to-treat tumorigenic prostate cancer at the earliest possible stage.

## Methods

All methods were carried out in accordance with relevant guidelines and regulations under approval from the University of York’s Biology Ethics Committee (https://www.york.ac.uk/biology/current-students-staff/ethics/bec/) (biol-ethics@york.ac.uk). The cell lines used in this study are immortalised cell lines, and not primary cell cultures.

**Cell lines:** PNT2-C2: A well-differentiated, normal prostate epithelial cell line derived from prostate luminal secretory cells obtained from the prostate tissue of a 33-year old male post mortem^17^. The cell line was immortalised via transfection with simian virus 40 (SV40). Prostate specific markers, AR (androgen receptor protein marker), PSA (prostate specific antigen) and PAP (prostatic acid phosphatase), are not detected. PNT2-C2 is a sub-clone developed from a parental clonal cell line (PNT). Its original purpose was as a means of establishing a consistent *in vivo* cell-line model of prostate disease states by using it to develop other sub-clonal lines via transfection. PNT2-C2 was obtained from Professor Norman Maitland’s laboratory at the University of York. See Refs.^16–18,64^.

P4E6: A well-differentiated, low-grade epithelial prostate-cancer cell line derived from a primary tissue sub-set expressing higher relative 8p allelic imbalance and characteristic of well-differentiated prostate cancers. P4E6 expresses the prostate specific marker, PSA. It is AR negative, and is representative of a Gleason grade 3 cancer. Immortalisation of P4E6 was achieved by transfection with the HPV E6 gene. P4E6 was derived at the University of York and obtained from Professor Norman Maitland’s laboratory. It is also available from the European Collection of Authenticated Cell Cultures (ECACC) at https://www.culturecollections.org.uk/nop/product/p4e6. See also Refs. ^16,65^.

**Cell culturing:** Both the PNT2-C2 and P4E6 cell lines were cultured in T75 tissue culture-treated flasks. PNT2-C2 was grown in RPMI (Roswell Park Memorial Institute-1640, Gibco) medium with 10% foetal calf serum (FCS) (R10 media) and 2mM L-glutamine. P4E6 was grown in KSFM (Keratinocyte Serum-Free Medium, Invitrogen) media with $50\mu \hbox {g/ml}$ bovine pituitary extract (BPE), 5ng/ml human epidermal growth factor (EGF) supplements, 2% FCS (K2 media) and 2mM L-glutamine. The cells were incubated at $37^{\circ }\hbox {C}$ in a humidified atmosphere containing 5% $\hbox {CO}_{{2}}$. No antibiotics were used during standard culture conditions.

**Sample preparation for Raman spectroscopy:** Preparation of the cell samples for Raman spectroscopy analysis follows a three-day protocol. On day one, cells were prepared by washing in PBS followed by incubation in trypsin and re-suspension in R10 media to inactivate the trypsin. The cells were then centrifuged, resuspended in the appropriate media (as above) and counted using a haemocytometer. A $\hbox {CaF}_2$ Raman grade 13mm (D) x 1mm (T) disc (Crystran Limited, Poole UK) was placed in a 35mm tissue culture dish. 50,000 cells were plated onto the disc in 200$\mu$l of media and left for 10 minutes to adhere. Media was then added to a total volume of 2.5ml, with antibiotic-antimycotic (ABM) solution (Thermo Fisher Scientific) since the discs are not sterile. The dishes were then placed in a $37^{\circ }\hbox {C}$ incubator. On the second day, media was changed to starvation media (as follows) to synchronise the cells so that they were not in the process of dividing, which would skew results. Starvation media for the PNT2-C2 cells grown in RPMI + 10% FCS comprised RPMI only, minus FCS and L-glutamine. For P4E6, starvation media was KSFM only, minus the supplements, FCS and L-glutamine. On the third day for live cell analysis, the cells were washed three times in HBSS buffer and then 2.5ml of fresh HBSS buffer was added to the dish. The dish containing the disc was then taken to the Raman microscope for analysis. For dried-cell analysis, the cells were washed three times in HBSS and then air desiccated (open air dried) for 30-minutes in a fume cupboard before Raman spectroscopy measurements. This amount of time ensured the cells were fully dried, yet with their biology best preserved in the air-dried state.

**Raman spectroscopy measurements:** Raman point spectra were collected using an HORIBA XploRA micro-Raman in confocal setting ($100 \,\mu \hbox {m}$ pinhole), with $200 \,\mu \hbox {m}$ slit, and 532 nm laser wavelength at 3.5 mW laser-power. For live-cell measurements, a Zeiss Wplan Apochromat 63X (NA = 1.0) Ph3 dipping lens was used, and for dried-cell measurements, an 100X (NA = 0.9) MPLN Olympus air objective was used. For both lenses, the diffraction-limited laser spot size using the 532 nm laser was $\sim 1\,\mu \hbox {m}$. A spectral resolution of $3\,\hbox {cm}^{-1}$ was obtained using a 2400 lines/mm diffraction grating. Spectra were acquired using 45s laser exposure averaged over two spectral acquisitions. A cell-viability test using trypan blue was performed, which showed the Raman measurements were non-destructive (Fig. S1). The cells were also monitored during real-time acquisition to ensure no spectral changes, and optical inspection was also performed after each measurement. In the live-cell study, single-cell measurements were performed with one spectrum per randomly chosen cell nuclei taken across the cell population. In the dried-cell study, five random spectra were obtained per optically well-defined and demarcated nucleus of randomly selected dried cells per cell population. In addition to cell-culturing processes that best removed apoptotic cells due to their detachment (inability to seed), we also excluded from the random selection process in the dried-state, those that had morphological features associated with apoptotic cells. Namely, we only sampled dried cells with regular, intact edges and surfaces (no evidence of blebbing, excessive thickening of the nuclear membrane or significant loss of membrane integrity / leakage) and having intact cellular and nuclear structures (i.e., no fractionation). See for example Ref.^66^, which describes the identification of morphological apoptotic cell features.

**Spectral pre-processing:** The spectra were first cut using the Raman Tool Set version 2.1.0^67^ to the fingerprint (600–$1800\,\hbox {cm}^{-1}$) and high-wavenumber (2700–$3100\,\hbox {cm}^{-1}$) ranges. Each spectrum was interpolated using code written in IGOR Pro Version 9.01 (WaveMetrics, Inc., Lake Oswego, OR, USA) to ensure the same wavenumber increments across the spectra for follow-on PCA analyses. The spectra had minimal background removal using the Raman Tool Set as the inclusion of spectral background has been shown to be beneficial in discriminating cell phenotypes (see for example, Ref. ^68^. Therefore, for the fingerprint regions of the live and dried cell Raman spectra, and for spectra in the dried-cell high-wavenumber region, an end-to-end linear baseline was applied. However, due to additional HBSS background in the high-wavenumber region of the live-cell spectra, an $\hbox {n}=3$ polynomial background subtraction was required. Following background subtraction, further pre-processing was performed on each spectrum using the Raman Tool Set, namely, total area normalisation, and spectral smoothing using a 0.65 cubic spline.

**Convergence testing:** Convergence of the average spectrum, twice the standard deviation (2xSD) and standard-error of the mean (SE) for increasing numbers of spectra ensured the data sets were statistically-representative of population-level, live-cell and dried-cell states^15^. Statistical convergence is shown for P4E6 with 137 live-cell and 112 dried-cell spectra collected in the fingerprint region, and 136 live-cell and 112 dried-cell spectra collected in the high-wavenumber region. For statistical convergence of PNT2-C2, there were 151 live-cell and 154 dried cell-spectra collected in the fingerprint region, and 152 live-cell and 154 dried-cell spectra collected in the high-wavenumber region. The results from the statistical convergence tests are in Figs. S2–S6 of the SI. Strict convergence of the statistical quantities ensured experimental variability and molecular-scale heterogeneity were fully accounted for.

**Principal component analysis for multivariate dimension reduction:** Principal component analysis (PCA) was performed with loadings and scatter plots produced using code written in R and executed in RStudio version 2022.07.2+576^69^.

**Linear discriminant analysis:** After PCA, linear discriminant analysis (LDA) was performed followed by leave-one-out cross-validation using code written in R and executed in RStudio version 2022.07.2+576^69^. The optimal number of PCs for LDA inclusion was determined by assessing the stability of the leave-one-out cross validation result as a function of the number of PCs included about the Kaiser criterion point with respect to the proportional variance captured per principal component (PC), and cumulated variance captured as a function of increasing principal components (PCs) (Table. S4 and Figs. S7, S8). Leave-one-out cross validation (LOOV) was used to determine the classification accuracy, as well as to check the numerical stability of the PCA-LDA result. Histograms for the LDA classification result were then generated. An LDA-weighted, loadings summation over the included PCs (sum-weighted loadings) was obtained, as well as measures for the group-mean separations, within-group variance, and ratio of the within-to-between-group variances determined using in-house code written in R. Biomarkers from the loadings results were deemed viable if they were significantly prominent above the fluctuating loadings background. See SI Tables S5–S12 and Figs. S9–S17.

**Peak intensity ratio (PIR) analyses:** Gaussian peak fitting was performed on the statistically converged, normalised average spectra per cell line across linear-baselined, local spectral windows using the Multipeak Fitting 2 function in IGOR Pro Version 9.01 (WaveMetrics, Inc., Lake Oswego, OR, USA). The fitted peak intensities were used to obtain disease-state biomarkers that differentiate the live and dried disease states via peak-intensity subtractions. Robust biomarkers were determined under criteria where the magnitude of the disease-state marker needed to be greater than the combined, fitted standard-error uncertainties corresponding to each de-convolved band per individual spectrum, with these also being checked against their significance in the sum-weighted loadings, and individual PC loadings. See SI Tables S3, S5–S12.

## Supplementary Information

Supplementary Information.

## Data Availability

The datasets generated and/or analysed during this study are available by request from Y.H. from the University of York repository (10.15124/802c9711-c3cd-4470-aa11-5624bc4854b9).
