# Supplementary material for: Raman spectroscopy reveals oxidative stress-induced metabolic vulnerabilities in early-stage AR-negative prostate-cancer versus normal-prostate cell lines
Source: Sci Rep. 2024 Oct 25;14:25388. doi: 10.1038/s41598-024-70338-1 (PMC11512068; doi:10.1038/s41598-024-70338-1)
Supplement: Supplementary file 1 — Supplementary Information. [file 41598_2024_70338_MOESM1_ESM.pdf]

Supplementary Information

Raman spectroscopy reveals oxidative stress–induced metabolic vulnerabilities in early-stage AR-negative prostate-cancer versus normal-prostate cell lines

M. Cameron,[1] F. Frame,[2,3] N. J. Maitland,[2,3] and Y. Hancock[1,3,*]

[1] *School of Physics, Engineering and Technology, University of York, Heslington, York, U.K., YO10 5DD*

[2] *Department of Biology, University of York, Heslington, York, U.K., YO10 5DD*

[3] *York Biomedical Research Institute, University of York, Heslington, York, U.K., YO10 5DD*

[*] y.hancock@york.ac.uk

I. SUPPLEMENTARY TABLES

A. RAMAN BAND POSITIONS FOR THE CELL TYPES IN THIS STUDY

TABLE S1: Raman band positions (cm^{-1}) for the standard cell lines in this study (live and dried) and the average calculated band positions. The peak assignments were obtained by fitting to the statistically-converged average spectrum for each cell line, with each average spectrum minimally baselined with no other significant pre-processing. For the fingerprint region, a two-point linear baseline between the ends of the averaged spectrum was used for both the live and the dried cell spectral fittings. For the high-wavenumber region, a three-point (n=3), polynomial baseline was used in the live-cell studies, and a two-point, linear baseline between the ends of the average spectrum was used in the dry-cell studies. Peak assignments were then obtained using Gaussian peak-fitting in linear baselined, local de-convolved band regions (alphabetically indicated). In a small number of cases, where there were missing or anomalous peak assignments caused by low signal-to-noise or being outside of experimental uncertainty of ± 3 cm^{-1}, then these have been indicated as underlined, or missing.

**Fitting region**	**PNT2-C2 Live**	**PNT2-C2 Dried**	**P4E6 Live**	**P4E6 Dried**	**Average**
A	618	619	617	618	**618**
B	640	640	640	641	**640**
B	671	667	671	667	**669**
C	698	698	696	695	**697**
C	714	716	713	714	**714**
C	724	724	724	724	**724**
D	745	745	744	745	**745**
D	756	754	756	756	**756**
D	781	783	781	780	**781**
E	807	809	806	807	**807**
E	825	825	825	826	**825**
F1	850	850	850	851	**850**
F2	875	876	876	874	**875**
F2	892	885	893	890	**890**
F3	934	934	935	932	**934**
F3	969	965	—	971	**968**
F3	1001	1001	1000	1000	**1001**
H1	1029	1030	1029	1029	**1029**
H2	1062	1060	1062	1061	**1061**
H2	1089	1090	1090	1088	**1089**

Continued on next page

TABLE S1 (continued): Raman band positions (cm^{-1})

**Fitting region**	**PNT2-C2 Live**	**PNT2-C2 Dried**	**P4E6 Live**	**P4E6 Dried**	**Average**
I	1125	1125	1124	1124	**1124**
J1	1155	1155	1154	1152	**1154**
J2	1171	1171	1171	1171	**1171**
K	1205	1206	1204	1204	**1205**
L1	1230	1239	1232	1230	**1233**
L2	1253	1259	1253	1248	**1253**
M1	1307	1306	1306	1306	**1306**
M1	1337	1335	1336	1336	**1336**
M2	1398	—	1400	—	**1399**
N1	1448	1448	1449	1448	**1448**
N2	1483	1483	1482	1482	**1482**
O1	1552	1552	1550	1552	**1551**
O2	1576	1574	1575	1575	**1575**
P1	1602	1603	1602	1604	**1603**
P2	1615	1616	1615	1614	**1615**
Q1	1656	1656	1654	1655	**1655**
Q2	1682	1681	1679	1679	**1680**
R	—	1746	—	1744	**1745**
HW1	2725	2728	2726	2726	**2726**
HW2	2850	2852	2850	2851	**2851**
HW2	2871	2872	2872	2870	**2871**
HW2	2894	2895	2890	2895	**2893**
HW2	2932	2930	2932	2930	**2931**
HW2	2940	2940	2941	2938	**2940**
HW2	2966	2963	2967	2964	**2965**
HW2	3014	3015	3010	3015	**3013**
HW3	3064	3064	3063	3065	**3064**

B. BIOMOLECULE PEAK-ASSIGNMENTS OBTAINED FROM THE LITERATURE

TABLE S2: Peak ranges, general and detailed assignments including literature references.

**Peak Range (cm^{-1})**	**General assignment**	**Detailed biomolecule assignments**
614–620	Proteins	C–C twist of proteins [60]<br>Phenylalanine C–C twist [11][20][60]<br>Lipids (minor): cholesterol ester
639–643	Proteins	C–S stretch & C–C twist of tyrosine [60]<br>C–C twist of tyrosine [11][20][21]
665–678	DNA/RNA<br>& Proteins	Guanine and thymine ring breathing mode [21][60]<br>Tyrosine-G backbone in RNA [60]<br>Guanine ring breathing mode and C–S stretching mode of cystine [11]<br>Guanine, tyrosine and cystine [60]<br>Cystine [21]
695–699	Proteins	Aminoacid methionine ν(C–S) *trans* [60]
713–719	Proteins, lipids & DNA/RNA	Aminoacid methionine ν(C–S) *trans* [60]<br>$CN^{+/-}(CH_3)_3$ stretch in lipids [60]<br>C–N membrane phospholipid head stretch [11][60]<br>Adenine nucleotide peak [11][60]
723–726	DNA/RNA<br>& free adenine	DNA [20][60]<br>Adenine [11][21][60][71]
743–746	DNA	Thymine ring breathing mode [20][60]
754–760	Proteins	Symmetric breathing of tryptophan [11]<br>Tryptophan ring breathing mode [60]<br>Tryptophan [11][21][25]
781–784	DNA/RNA	Uracil, Cytosine and thymine ring breathing modes [20][21][25][60]<br>Nucleic acids [9]<br>O–P–O stretch in DNA [60]
806–810	DNA/RNA	O–P–O stretch RNA [60]

Continued on next page

TABLE S2 (continued): Biomolecule peak-assignments obtained from the literature.

Peak Range (cm^{-1})	General assignment	Detailed biomolecule assignments
821–828	Proteins	Tyrosine out-of-plane ring breathing and O–P–O stretch in DNA [11][21][25][60]
	& DNA/RNA	DNA backbone (O–P–O) [9]
		Proline and hydroxyproline [60]
		Out-of-plane tyrosine ring breathing [20]
848–853	Proteins	Tyrosine ring breathing [20][25][60]
		C–C stretch in proline and tyrosine ring breathing mode [11][21]
	& Carbo.	Carbohydrates: glycogen, polysaccharides C–O-C stretch [60]
875–880	Proteins	Proteins C–C stretch, antisymmetric vibrations of choline $N(CH_3)_3$ (phospholipids), hydroxyproline, tryptophan (proteins) [60]
		Tryptophan [20][60]
885–896	Carbohydrates & Proteins	Saccharide band, methylene and C–C skeletal, proteins (protein modes for tumours) [60]
933–936	Proteins	Skeletal C–C stretch α-helix (protein), proline, valine [11][60]
	& Carbohydrates	C–C backbone stretch α-helix [25][9][21]
		C–O-C glycogen (carbohydrate) [60]
965–976	Lipids, Proteins & DNA/RNA	Lipids (at 968) mostly; phosphate monoester groups of phosphorylated proteins and cellular nucleic acids [60]
999–1001	Proteins	Phenylalanine ring breathing [11][20][21][25][60]
		Bound & free NADH [60]
1022–1033	Proteins (namely),	Phenylalanine C–H in-plane bending mode [11][20][21][25][60]; C–N stretch of proteins [60]
	Lipids	CH_2CH_3 bending phospholipids [60]
	& Carbohydrates	Glycogen [60]
1060–1070	Lipids, DNA/RNA	Lipids chain C–C stretch [11][25][60]

Continued on next page

TABLE S2 (continued): Biomolecule peak-assignments obtained from the literature.

**Peak Range (cm^{-1})**	**General assignment**	**Detailed biomolecule assignments**
	& Carbohydrates	DNA/RNA PO_2^- backbone stretch [60]<br>Carbo. C–O, C–C stretching
1087–1091	DNA, Lipids<br><br>& Proteins	DNA PO_2^- backbone stretch and C–C stretch (gauche) in lipids [11][60]<br>C–N of Proteins [60]<br>Lipids [9]
1123–1126	Lipids, Proteins<br>& Carbohydrates	Protein C–N stretch [21][25][60]<br>C–C stretch in lipids [11][21][25][60]<br>C–C stretch in proteins [11][60]<br>C–N stretch (glucose), C–O & C–C stretch in carbohydrates [11][60]<br>Phospholipids [9]
1153–1155	Proteins<br>& Carbohydrates	C–C and C–N stretch in proteins [20][25][60]<br>Carotenoids—correlated to disease [60]<br>Glycogen [60]
1169–1172	Proteins	(CH) phenylalanine, tyrosine [60]<br>Bending mode (in-plane) tyrosine [11][25]
1202–1206	Proteins &<br>DNA/RNA	Tyrosine and phenylalanine [60]<br>Phenylalanine and tryptophan [11][25][60]<br>Phenylalanine [20]<br>CH_2 wagging of glycine and proline [60]<br>Amide III [60]<br>A,T ring breathing modes of DNA/RNA [60]<br>Hydroxyproline & tyrosine [11]
1228–1239	Proteins & DNA/RNA	Amide III and CH_2 wagging of glycine and proline [60]<br>PO_2^- antisymmetric stretch [60]; Amide III [20][21][25]
1249–1259	Proteins, DNA/RNA & Lipids	Amide III, guanine, cytosine (NH_2); A, T in DNA/RNA; CH_2 deformation in lipids; formalin damage [60]

Continued on next page

TABLE S2 (continued): Biomolecule peak-assignments obtained from the literature.

Peak Range (cm^{-1})	General assignment	Detailed biomolecule assignments
1306–1309	Proteins, Lipids	CH_2 deformation in lipids, adenine and cytosine [11][60]
	& Nucleic acids	CH_3/CH_2 modes in lipids / collagen [11][60]
		C–N stretch in amines [60]
		Nucleic acids [9]
1336 –1339	Proteins, DNA/RNA & Lipids	CH_3CH_2 wagging, twisting &/or bending of collagen, lipids, tryptophan & nucleic acids (adenine and guanine) [60]
		Amide III, phenyl group (phenylalanine) [60]
		CH_2 deformation in lipids, adenine and cytosine [11]
		Adenine and guanine; protein C–H deformation [25]; Adenine [21]
1396–1400	Proteins	C=O, CH_2, NH deformation [60]
1448–1450	Lipids, proteins & DNA/RNA	CH_2 deformation in lipids and proteins; at 1450 cm^{-1} in malignant tissues [21][60]
		CH_2 bending mode of proteins [20][11]
		C–H deformation & vibration in lipids and proteins [25][60]
		CH_2CH_3 deformation [60]
		Nuclear acids [9]; CH_2 in-plane deformation (ring stretch) in DNA/RNA [21]
1482 –1485	Protein & DNA/RNA	Amide II CN stretch & N-H bend [60]
		Guanine and adenine ring-breathing modes [11][21][60]
1550–1555	Proteins	Tryptophan [11][60]; Amide II [60]
1574–1579	DNA/RNA &	Guanine and adenine ring-breathing modes [11][21][25][60]
		Nucleic acids [9][11]
	Proteins	Bound and free NADH [60]

Continued on next page

TABLE S2 (continued): Biomolecule peak-assignments obtained from the literature.

**Peak Range (cm^{-1})**	**General assignment**	**Detailed biomolecule assignments**
1602–1605	Proteins	C=C bending mode of phenylalanine and tyrosine [11][25][60]; C–C ring stretch of phenyl [60]; C–C and C–N stretch (proteins) [20]
1613–1616	Proteins	Tyrosine, tryptophan C=C stretch [11][21][25][60], bound and free NADH [60]
1654–1659	Proteins, DNA/RNA & lipids	Amide I C=O stretch, α-helix conformation; proteins [11][25][60],<br>α-helix proteins & disordered proteins [9]<br>DNA/RNA T, G, C [60]<br>C=C stretch of lipids [11][13][25][60]
1674–1686	Lipids & Proteins	C=C cholesterol; amide I [60]
1744–1754	Lipids	C=O carboxyl stretch [60]
2850–2853	Lipids	CH_2 symmetric stretch in lipids [13][14][60]
2871–2874	Lipids & proteins	CH_2 asymmetric stretch in lipids and proteins [60]; CH_2 symmetric stretch in lipids [60]; CH_3 symmetric stretch in lipids & proteins [13]
2890–2896	Lipids & proteins	CH stretch of lipids and proteins (predominantly) and CH_3 symmetric stretch [60]<br>CH_2 deformation [60]
2930–2935	Proteins & lipids	CH_2 asymmetric stretch [13][60], CH_3 symmetric stretch (predominantly proteins) [60]
2940–2943	Lipids & proteins	C–H vibrations in lipids & proteins. CH_2 modes in lipids [60]
2963–2969	Lipids	CH_3 asymmetric stretch in lipids, cholesterol & cholesterol ester [14][60]
3009–3017	Lipids	Unsaturated =CH stretch in lipids [60]<br>=CH stretch (unsaturated lipids) [13][14]
3063–3065	Proteins	CH stretch [60]<br>CH stretch in proteins; aromatic residues: phenylalanine, tyrosine & tryptophan [72]

C. GAUSSIAN PEAK-FITTING RESULTS (KEY BANDS)

TABLE S3: Gaussian peak-fitting results for key bands. Uncertainties range from 1–6% SE per fitted band.

Band	C−N(L)	C−N(D)	$\Delta C_{rel.}$	C(L−D)	N(L−D)	$\Delta D_{rel.}$	Assignments
618	5.4E-5	1E-6	$\|\Delta_L \uparrow\| > \|\Delta_D \uparrow\|$	1.10E-4	5.8E-5	$\|\Delta_C \downarrow\| > \|\Delta_N \downarrow\|$	Proteins: phenylalanine; Lipids (minor): cholesterol ester
640	5E-6	3.0E-5	$\|\Delta_L \uparrow\| < \|\Delta_D \uparrow\|$	1.3E-5	3.7E-5	$\|\Delta_C \downarrow\| < \|\Delta_N \downarrow\|$	Proteins: tyrosine
669	8E-6	8.4E-5	$\|\Delta_L \uparrow\| < \|\Delta_D \uparrow\|$	−1.5E-5	6.1E-5	$\|\Delta_C \uparrow\| < \|\Delta_N \downarrow\|$	DNA/RNA: G,T,C bases; Proteins: cystine, tyrosine
697	−3E-6	7.0E-5	$\|\Delta_L \downarrow\| < \|\Delta_D \uparrow\|$	5E-6	7.8E-5	$\|\Delta_C \downarrow\| < \|\Delta_N \downarrow\|$	Proteins: methionine
714	−1.2E-4	−9.1E-5	$\|\Delta_L \downarrow\| > \|\Delta_D \downarrow\|$	6.3E-5	8.0E-5	$\|\Delta_C \downarrow\| < \|\Delta_N \downarrow\|$	Lipids: C–N, $CN^{+/-}(CH_3)_3$; DNA/RNA: nucleotide peak; adenine; Proteins: methionine
724	6.2E-5	2.9E-5	$\|\Delta_L \uparrow\| > \|\Delta_D \uparrow\|$	2.8E-5	−5E-6	$\|\Delta_C \downarrow\| > \|\Delta_N \uparrow\|$	DNA: adenine; free adenine
745	−2.2E-4	−3.7E-5	$\|\Delta_L \downarrow\| > \|\Delta_D \downarrow\|$	2.61E-4	4.46E-4	$\|\Delta_C \downarrow\| < \|\Delta_N \downarrow\|$	DNA: thymine
781	−2.4E-5	1.0E-5	$\|\Delta_L \downarrow\| > \|\Delta_D \uparrow\|$	8.7E-5	1.21E-4	$\|\Delta_C \downarrow\| < \|\Delta_N \downarrow\|$	DNA/RNA: uracil, cytosine & thymine; O–P–O DNA
807	9E-6	3.6E-5	$\|\Delta_L \uparrow\| < \|\Delta_D \uparrow\|$	−2.8E-5	−1E-6	$\|\Delta_C \uparrow\| > \|\Delta_N \uparrow\|$	RNA: O–P–O stretch
825	6E-6	−4.9E-5	$\|\Delta_L \uparrow\| < \|\Delta_D \downarrow\|$	7.4E-5	1.9E-5	$\|\Delta_C \downarrow\| > \|\Delta_N \downarrow\|$	Proteins: tyrosine, (hydroxy)proline; DNA/RNA: O–P–O DNA stretch
850	9.1E-5	2.2E-5	$\|\Delta_L \uparrow\| > \|\Delta_D \uparrow\|$	8.1E-5	1.2E-5	$\|\Delta_C \downarrow\| > \|\Delta_N \downarrow\|$	Proteins: tyrosine, proline; Carbo: glycogen, polysacch.
875	−7E-6	2.3E-5	$\|\Delta_L \downarrow\| < \|\Delta_D \uparrow\|$	−2.4E-5	6E-6	$\|\Delta_C \uparrow\| > \|\Delta_N \downarrow\|$	Proteins: tryptophan, proline, valine; Lipids: choline
968	−1.56E-4	−1.00E-4	$\|\Delta_L \downarrow\| > \|\Delta_D \downarrow\|$	−7.4E-5	−1.8E-5	$\|\Delta_C \uparrow\| > \|\Delta_N \uparrow\|$	Lipids: C–C (mostly)
1001	1.12E-4	2.10E-4	$\|\Delta_L \uparrow\| < \|\Delta_D \uparrow\|$	2.32E-4	3.30E-4	$\|\Delta_C \downarrow\| < \|\Delta_N \downarrow\|$	Proteins: Phenylalanine ring breathing; Bound and free NADH
1029	2.7E-5	1.1E-5	$\|\Delta_L \uparrow\| > \|\Delta_D \uparrow\|$	1.13E-4	9.7E-5	$\|\Delta_C \downarrow\| > \|\Delta_N \downarrow\|$	Proteins: C–N & Phenylalanine (C–H); Phospholipids; Carbo.: glycogen
1061	6.0E-6	3.9E-5	$\|\Delta_L \uparrow\| < \|\Delta_D \uparrow\|$	−5.4E-5	−2.1E-5	$\|\Delta_C \uparrow\| > \|\Delta_N \uparrow\|$	Lipids: C–C; DNA/RNA: PO_2^- backbone; Carbo.: C–O, C–C stretching

Continued on next page

TABLE S3 (continued): Gaussian peak-fitting results for key bands

Band	C−N(L)	C−N(D)	$\Delta C_{rel.}$	C(L−D)	N(L−D)		Assignments
1124	−4.9E-5	8.8E-5	$\lvert\Delta_L \downarrow\rvert < \lvert\Delta_D \uparrow\rvert$	1.6E-5	1.53E-4	$\lvert\Delta_C \downarrow\rvert < \lvert\Delta_N \downarrow\rvert$	C–C Lipids, Carbo. & Proteins; C–N Glucose; C–O Carbo.
1154	3.8E-5	3.1E-5	$\lvert\Delta_L \uparrow\rvert > \lvert\Delta_D \uparrow\rvert$	5.5E-5	4.8E-5	$\lvert\Delta_C \downarrow\rvert > \lvert\Delta_N \downarrow\rvert$	Proteins: C–C, C–N stretch; Carbo.: glycogen; carotenoids
1171	1.0E-5	−3.7E-5	$\lvert\Delta_L \uparrow\rvert < \lvert\Delta_D \downarrow\rvert$	1.02E-4	5.5E-5	$\lvert\Delta_C \downarrow\rvert > \lvert\Delta_N \downarrow\rvert$	Proteins: phenylalanine and tyrosine
1205	8E-6	6.9E-5	$\lvert\Delta_L \uparrow\rvert < \lvert\Delta_D \uparrow\rvert$	−2E-6	5.9E-5	$\lvert\Delta_C \uparrow\rvert < \lvert\Delta_N \downarrow\rvert$	DNA/RNA: A,T bases; Proteins: phenylalanine, tryptophan, amide III, glycine, tyrosine & (hydroxy)proline
1233	−4.9E-5	2E-6	$\lvert\Delta_L \downarrow\rvert > \lvert\Delta_D \uparrow\rvert$	7.7E-5	1.29E-4	$\lvert\Delta_C \downarrow\rvert < \lvert\Delta_N \downarrow\rvert$	Proteins: amide III, glycine & proline; DNA/RNA: PO_2^-
1253	−4.2E-5	−7.1E-5	$\lvert\Delta_L \downarrow\rvert < \lvert\Delta_D \downarrow\rvert$	9.0E-5	6.0E-5	$\lvert\Delta_C \downarrow\rvert > \lvert\Delta_N \downarrow\rvert$	DNA/RNA: guanine & cytosine; Proteins: amide III & Lipids
1306	9.7E-5	−4.5E-5	$\lvert\Delta_L \uparrow\rvert > \lvert\Delta_D \downarrow\rvert$	2.00E-4	5.8E-5	$\lvert\Delta_C \downarrow\rvert > \lvert\Delta_N \downarrow\rvert$	Proteins: C–N amines; DNA/RNA: adenine, cytosine. CH_2, CH_3/CH_2. Lipids
1336	1.78E-4	−1.4E-5	$\lvert\Delta_L \uparrow\rvert > \lvert\Delta_D \downarrow\rvert$	3.25E-4	1.33E-4	$\lvert\Delta_C \downarrow\rvert > \lvert\Delta_N \downarrow\rvert$	Proteins: tryptophan, phenylalanine, amide III, CH deform.; DNA/RNA: A, G. CH_3/CH_2. Lipids (minor)
1399	9.2E-6	−8E-6	$\lvert\Delta_L \uparrow\rvert > \lvert\Delta_D \downarrow\rvert$	2.6E-5	8.8E-6	$\lvert\Delta_C \downarrow\rvert > \lvert\Delta_N \downarrow\rvert$	Proteins: C=O, CH_2, NH
1448	−5.0E-5	2.50E-4	$\lvert\Delta_L \downarrow\rvert << \lvert\Delta_D \uparrow\rvert$	−3.60E-4	−6.0E-5	$\lvert\Delta_C \uparrow\rvert > \lvert\Delta_N \uparrow\rvert$	Lipids & Proteins: CH_2, CH_2CH_3, C–H, also malig./cell death; DNA/RNA
1482	6E-6	4.9E-5	$\lvert\Delta_L \uparrow\rvert < \lvert\Delta_D \uparrow\rvert$	−3.3E-5	1.0E-5	$\lvert\Delta_C \uparrow\rvert > \lvert\Delta_N \downarrow\rvert$	Proteins: amide II, CN & N-H deform. DNA/RNA: G,A bases
1575	−5.0E-6	3.5E-5	$\lvert\Delta_L \downarrow\rvert < \lvert\Delta_D \uparrow\rvert$	7.3E-5	1.13E-4	$\lvert\Delta_C \downarrow\rvert < \lvert\Delta_N \downarrow\rvert$	DNA/RNA: nucleic acids, guanine, adenine; Proteins: Bound and free NADH
1603	6.1E-5	−6.0E-5	$\lvert\Delta_L \uparrow\rvert > \lvert\Delta_D \downarrow\rvert$	8.3E-5	−3.8E-5	$\lvert\Delta_C \downarrow\rvert > \lvert\Delta_N \uparrow\rvert$	Proteins: C–C, C=C, C–N phenylalanine & tyrosine

Continued on next page

TABLE S3 (continued): Gaussian peak-fitting results for key bands

Band	C−N(L)	C−N(D)	$\Delta C_{rel.}$	C(L−D)	N(L−D)		Assignments
1615	3.3E-5	−3.0E-6	$\lvert\Delta_L\uparrow\rvert >> \lvert\Delta_D\downarrow\rvert$	7.8E-5	4.2E-5	$\lvert\Delta_C\downarrow\rvert > \lvert\Delta_N\downarrow\rvert$	Proteins: tyrosine, tryptophan C=C, bound and free NADH
1655	−3.1E-5	1.02E-4	$\lvert\Delta_L\downarrow\rvert < \lvert\Delta_D\uparrow\rvert$	1.37E-4	2.70E-4	$\lvert\Delta_C\downarrow\rvert < \lvert\Delta_N\downarrow\rvert$	Proteins: amide I, α-helix; DNA/RNA; Lipids: C=C
2726	−4.6E-5	−6.1E-5	$\lvert\Delta_L\downarrow\rvert < \lvert\Delta_D\downarrow\rvert$	−8.9E-5	−1.05E-4	$\lvert\Delta_C\uparrow\rvert < \lvert\Delta_N\uparrow\rvert$	Lipids: C–H stretches
2851	−4.81E-4	9.2E-5	$\lvert\Delta_L\downarrow\rvert > \lvert\Delta_D\uparrow\rvert$	−6.82E-4	−1.09E-4	$\lvert\Delta_C\uparrow\rvert > \lvert\Delta_N\uparrow\rvert$	Lipids: CH_2
2893	−3.49E-4	3.35E-4	$\lvert\Delta_L\downarrow\rvert > \lvert\Delta_D\uparrow\rvert$	−4.75E-4	2.09E-4	$\lvert\Delta_C\uparrow\rvert > \lvert\Delta_N\downarrow\rvert$	Lipids and Proteins: CH, CH_2 & CH_3
2931	6.5E-5	−8.15E-4	$\lvert\Delta_L\uparrow\rvert < \lvert\Delta_D\downarrow\rvert$	-1.8E-5	−8.97E-4	$\lvert\Delta_C\uparrow\rvert < \lvert\Delta_N\uparrow\rvert$	Proteins and Lipids: CH_2 & CH_3 (predominantly proteins)
2940	8.3E-5	−3.28E-4	$\lvert\Delta_L\uparrow\rvert < \lvert\Delta_D\downarrow\rvert$	8.78E-4	4.67E-4	$\lvert\Delta_C\downarrow\rvert > \lvert\Delta_N\downarrow\rvert$	Proteins and Lipids: C–H lipids & proteins; CH_2 modes in lipids
2965	2.80E-4	−1.62E-4	$\lvert\Delta_L\uparrow\rvert > \lvert\Delta_D\downarrow\rvert$	7.52E-4	3.10E-4	$\lvert\Delta_C\downarrow\rvert > \lvert\Delta_N\downarrow\rvert$	Lipids: CH_3 asymmetric stretch; cholesterol & cholesterol ester
3013	−6.87E-5	−1.42E-4	$\lvert\Delta_L\downarrow\rvert < \lvert\Delta_D\downarrow\rvert$	1.26E-4	5.3E-5	$\lvert\Delta_C\downarrow\rvert > \lvert\Delta_N\downarrow\rvert$	Lipids: unsaturated =CH stretch in lipids
3064	1.05E-4	−9.8E-7	$\lvert\Delta_L\uparrow\rvert > \lvert\Delta_D\downarrow\rvert$	1.57E-4	5.1E-5	$\lvert\Delta_C\downarrow\rvert > \lvert\Delta_N\downarrow\rvert$	Proteins: CH stretch; phenylalanine, tyrosine & tryptophan

D. LEAVE-ONE-OUT, CROSS-VALIDATION STABILITY CHECKS FOR LDA

TABLE S4. Leave-one-out cross-validation (LOOV %-accuracy) stability checks for each of the cell line comparisons in this study (fingerprint and high-wavenumber calculations). The stability of the LOOV result is determined as a function of the number of PCs in the LDA classification model. Here, 'stability' refers to there being a stable minimum %-accuracy associated with the LOOV + SE determined over 1000 independent calculations. The number of PCs associated with the minimum %-accuracy LOOV (bold and underlined) is then used as the optimum input to the LDA classification model (PC_{LDA}). The Kaiser criterion point, which includes all PCs that have variance > 1% is highlighted. Graphs that show the variance for each PC and cumulative variance as a function of increasing numbers of PCs are in Figs. S7 and S8, respectively.

**Cell line comparisons**	**%-variance captured**	No. of PCs	LOOV Av. % error + SE
**PNT2-C2 live vs. dried**			
Fingerprint	**<u>62%</u>**	**<u>7</u>**	**0.3 ± 0**
305 spectra: 151 live & 154 dried	66% (Kaiser)	10	0.65 ± 0.01
High wavenumber	76%	6	0.49 ± 0.01
306 spectra: 152 live & 154 dried	**<u>77%</u>** (Kaiser)	**<u>7</u>**	**0.16 ± 0.01**
	80%	10	0.16 ± 0.01
	85%	19	0.16 ± 0.01
**P4E6 live vs. dried**			
Fingerprint	**<u>59%</u>**	**<u>6</u>**	**0**
249 spectra: 137 live & 112 dried	63% (Kaiser)	9	0.4 ± 0
High wavenumber	**<u>72%</u>**	**<u>7</u>**	**0**
248 spectra: 136 live & 112 dried	73% (Kaiser)	8	0
	80%	18	0.19 ± 0.01
**PNT2-C2 live vs. P4E6 live**			
Fingerprint	55%	6	10 ± 0
288 spectra: 151 PNT2-C2 & 137 P4E6	**<u>60%</u>** (Kaiser)	**<u>10</u>**	**2 ± 0**
	70%	27	1.9 ± 0.01
High wavenumber	72%	6	1.91 ± 0.01
288 spectra: 152 PNT2-C2 & 136 P4E6	74% (Kaiser)	8	0.87 ± 0.01
	75%	9	0.7 ± 0.01
	**<u>79%</u>**	**<u>15</u>**	0.34 ± 0.01
	80%	18	0.34 ± 0.01
	85%	28	0.34 ± 0.01
**PNT2-C2 dried vs. P4E6 dried**			
Fingerprint	**<u>54%</u>**	**<u>10</u>**	**0**
266 spectra: 154 PNT2-C2 & 112 P4E6	58% (Kaiser)	13	0.19 ± 0.01
High wavenumber	75%	5	1 ± 0
266 spectra: 154 PNT2-C2 & 112 P4E6	**<u>77%</u>** (Kaiser)	**<u>7</u>**	0.4 ± 0

E. P4E6 LIVE VS. PNT2-C2 LIVE (FINGERPRINT): KEY SEPARATING BANDS

TABLE S5. Col. 1: $\Delta = +/-$ or 0 average spectral difference per band [Fig. 1(a); SI Figs. S18, S19]. Col. 2: δ_{peak} = Gaussian peak-fitted difference for C(L)–N(L). Col. 3: PCA loadings. Col. 4: PCA-LDA weighted-loadings. Col. 5: peak-assignments. Bands deemed: (i) *significant* in separating the classes where $\delta_{peak} >> $ the sum of the fitted SE uncertainties at 1–6 % SE per fitted band are in **purple**, (ii) $\delta_{peak} >$ the sum of the fitted SE uncertainties are in **teal**, and (iii) $\simeq$ the sum of the fitted SE uncertainties are in plain text. Cf. SI Figs. S9, S17.

(Δ) Band	δ_{peak} (E-4)	PCA loadings	LDA$_W$	Assignments
**+618*[a]**	**+0.5**	**618 PC4**	—	Proteins: phenylalanine C–C twist; Lipids (minor): cholesterol ester
(0) 640[a]	+0.05	640 PC4	—	Proteins: tyrosine
(0) 669*	+0.08	669 PC4	669	DNA/RNA & Proteins: cystine, tyrosine
−697*	−0.03	697 PC4	697	Proteins: amino acid methionine (methion.)
**−714[b]**	**−1.1**	**714 PC4**	**714**	Lipids: C–N, $CN^{+/-}(CH_3)_3$; DNA/RNA: adenine; Proteins: methion.
**−724*[a,c]**	**+0.6**	**724 PC4,8**	—	DNA/RNA: adenine, free adenine
**−745***	**−2.2**	**745 PC4,8**	**745**	DNA: thymine
−756	−0.005	756 PC1,4	756	Proteins: tryptophan (tryptoph.)
**−781*[a,c]**	**−0.2**	**781 PC4,7,8**	**781**	DNA/RNA: uracil, cytosine & thymine; O–P–O DNA
(0) 807*[b]	0.09	807 PC4,8	807	RNA: O–P–O stretch
−825*[c]	0.06	825 PC4,7,8	—	DNA: tyrosine; DNA/RNA: O–P–O; Proteins: (hydroxy)proline
**−850*[a]**	**+0.9**	**850 PC4,7**	—	Proteins: tyrosine, proline; Carbo: glycogen, polysacch.
−875*	−0.07	—	—	Proteins: hydroxyproline, tryptoph.; Lipids: choline
**−890**	**+0.1**	**890 PC4**	—	Carbo: saccharide; Proteins; C–C skeletal; methylene
-934*[c]	+0.1	934 PC4,8	934	Proteins: α-helix, proline, valine; Carbo.: C–O-C glycogen
**−968***	**−1.6**	**968 PC4,8**	**968**	Lipids C–C (mostly); Phosphorylated proteins and cellular nucleic acids
**−1001*[a,b,e]**	**+1.1**	**1001 PC4,7,8**	**1001**	Proteins: Phenylalanine ring breathing
**−1029[a]**	**+0.3**	**1029 PC4**	**1029**	Proteins: C–N & Phenylalanine (C–H); Phospholipids, Carbo.
−1061*	+0.06	1061 PC4,8	1061	Lipids: C–C; DNA/RNA: PO_2^- backbone; Carbo. C–O, C–C stretching
−1089[b,c]	−0.1	1089 PC4,7,8	1089	DNA: PO_2^- backbone; Lipids: C–C; Proteins: C–N
**−1124*[a,d]**	**−0.5**	**1124 PC4,7**	—	C–C Lipids, Carbo. & Proteins; C–N Glucose; C–O Carbo.
**−1154**	**+0.4**	**1154 PC4,8**	**1154**	Proteins (C–C, C–N stretch) & Carbo. (glycogen); carotenoids
−1171*	+0.1	1171 PC4,7	1171	Proteins: phenylalanine & tyrosine
+1205*[a]	+0.08	1205 PC4,8	1205	DNA/RNA & Proteins: tyrosine, phenylalanine, tryptophan, glycine, proline, amide III
**−1233*[a]**	**−0.5**	**1233 PC4,7**	**1233**	Proteins, DNA/RNA: amide III, glycine, proline, PO_2^-
**−1253*[c,d,e]**	**−0.4**	**1253 PC4,7**	**1253**	DNA/RNA: guanine & cytosine; Proteins: amide III & Lipids
**+1306*[c,d,e]**	**+1.0**	**1306 PC4,7,8**	**1306**	Proteins: C–N amines; DNA/RNA: adenine, cytosine; CH_2, CH_3/CH_2. Lipids
**+1336*[e]**	**+1.8**	**1336 PC4,7,8**	**1336**	Proteins: tryptophan, phenylalanine, amide III, CH deform.; DNA/RNA: A, G. CH_3CH_2. Lipids (minor)
+1399[d]	+0.09	1399 PC4,7,8	—	Proteins: C=O, CH_2, NH
**+1448*[a,c,d,e]**	**−0.5**	**1448 PC4,8**	**1448**	Lipids & Proteins: CH_2, C–H, CH_2CH_3; DNA/RNA
+1482*	+0.06	1482 PC4,8	—	Proteins: amide II, C-N, N-H; DNA/RNA: guanine, adenine
+1551	−0.04	1551 PC4,7	—	Proteins: tryptophan, amide II
+1575*[b,c]	−0.05	1575 PC4,8	—	DNA/RNA: nucleic acids, guanine, adenine
**+1603*[a]**	**+0.6**	**1603 PC4,8**	**1603**	Proteins: phenylalanine, tyrosine, C–C, C=C, C–N
**+1615*[a,d]**	**+0.3**	**1615 PC4,8**	**1615**	Proteins: tyrosine, tryptophan C=C
+1655*[b,c]	−0.3	1655 PC4,7,8	1655	Proteins: amide I, α-helix; DNA/RNA: T, G, C bases; Lipids: C=C
+1680	+0.3	1680 PC4,8	1680	Lipids: C=C cholesterol; Proteins: amide I
(0)1745*	∼0	1745 PC4	—	Lipids: carboxyl group (C=O)

E. P4E6 LIVE VS. PNT2-C2 LIVE (HIGH WAVENUMBER): KEY SEPARATING BANDS

TABLE S6. Col. 1: Δ = +/− or 0 average spectral difference per band [Fig. 1(b); SI Figs. S18, S19]. Col. 2: δ_{peak} = Gaussian peak-fitted difference for C(L)–N(L). Col. 3: PCA loadings. Col. 4: PCA-LDA weighted-loadings. Col. 5: peak-assignments. Bands deemed: (i) *significant* in separating the classes where $\delta_{peak} >> $ the sum of the fitted SE uncertainties at 1–6 % SE per fitted band are in **purple**, (ii) $\delta_{peak} >$ the sum of the fitted SE uncertainties are in **teal**, and (iii) ≃ the sum of the fitted SE uncertainties are in plain text. Cf. SI Figs. S10, S17.

(Δ) Band	δ_{peak} (E-4)	PCA loadings	LDA_W	Assignments
**+2726***	**−0.5**	**2726 PC2,8**	**2726**	Lipids: C–H stretches
**−2851***[a,e,f]	**−4.8**	**2851 PC2,4,7,15**	—	Lipids: CH_2
−2871*	−0.4	2871 PC4,7,8	—	Lipids and Proteins: CH_2 & CH_3
**−2893***[e,f]	**−3.5**	**2893 PC2,4,7,8**	**2893**	Lipids and Proteins: CH, CH_2 & CH_3
+2931*	+0.6	2931 PC2,4,7,8	—	Proteins and Lipids: CH_2 & CH_3 (predominantly proteins)
**+2940***[a,f]	**+0.8**	**2940 PC2,4,8**	**2940**	Proteins and Lipids: C–H lipids & proteins; CH_2 modes in lipids
**+2965***[a,e,f]	**+2.8**	**2965 PC2,4,7**	**2965**	Lipids: CH_3 asymmetric stretch; cholesterol & cholesterol ester
**−3013***[a,e,f]	**−0.7**	**3013 PC2,4,7**	**3013**	Lipids: unsaturated =CH stretch in lipids
**+3064***	**+1.1**	**3064 PC4,8**	—	Proteins: CH stretch; phenylalanine, tyrosine & tryptophan

References for Tables S5 and S6

Prostate cancer vs. normal equivalent or treated:

[a] Corsetti et al. 2018 DU145 (metastatic prostate cancer) vs. PNT2-C2 (normal prostate) cell lines. Ethanol fixed.

[b] Taleb et al. 2006 LNCaP (metastatic prostate cancer) vs. PNT1A (normal) cell lines. Ethanol fixed.

[c] Crow et al. 2005 LNCaP, PCa 2b (AR-positive) and PC 3, DU145 (AR-negative) metastatic cell lines. Cells kept "moist".

[d] van Breugel et al. 2023 Prostate tissues from biopsy. Benign, Gleason 3, 4 and 5 comparisons. Formalin fixed.

[e] Potcoava et al. 2014 LNCaP and PC3 (metastatic cell lines). Untreated versus treated. Formaldehyde fixed.

[f] Hislop et al. 2022 PC3 and LNCaP versus PNT2 normal prostate cell line. Cells fixed with paraformaldehyde.

I. PNT2-C2 LIVE VS. DRIED (FINGERPRINT): KEY SEPARATING BANDS

TABLE S7. Col. 1: Δ = +/− or 0 average spectral difference per band [Fig. 1(a); SI Figs. S18, S19]. Col. 2: δ_{peak} = Gaussian peak-fitted difference for N(L)–N(D). Col. 3: PCA loadings. Col. 4: PCA-LDA weighted-loadings. Col. 5: peak-assignments. Bands deemed: (i) *significant* in separating the classes where $\delta_{peak} >> $ the sum of the fitted SE uncertainties at 1–6 % SE per fitted band are in **purple**, (ii) $\delta_{peak} >$ the sum of the fitted SE uncertainties are in **teal**, and (iii) $\simeq$ the sum of the fitted SE uncertainties are in plain text. Cf. SI Figs. S11, S17.

(Δ) Band	δ_{peak} (E-4)	PCA loadings	LDA$_W$	Assignments
**+618*[e]**	**+0.6**	**618 PC2,7**	—	Proteins: phenylalanine C–C twist; Lipids (minor): cholesterol ester
**+640**	**+0.4**	**640 PC7**	—	Proteins: tyrosine
**+669***	**+0.6**	**669 PC1,2**	**669**	DNA/RNA & Proteins: cystine, tyrosine
**+697***	**+0.8**	**697 PC2,7**	—	Proteins: aminoacid methionine (methion.)
**+714[d]**	**+0.8**	**714 PC2**	**714**	Lipids: C–N, CN$^{+/-}$(CH$_3$)$_3$; DNA: adenine; Proteins: methion.
+724*[f]	−0.05	724 PC1,2	724	DNA/RNA: adenine, free adenine
**+745***	**+4.5**	**745 PC1,2,4,7**	**745**	DNA: thymine
**+756**	**+0.5**	**756 PC2**	—	Proteins: tryptophan (tryptoph.)
**+781*[b]**	**+1.2**	**781 PC1,2,7**	**781**	DNA/RNA: uracil, cytosine & thymine; O–P–O DNA
−807*	−0.01	807 PC7	—	RNA: O–P–O stretch
**−825*[b,d,f]**	**+0.2**	**825 PC2**	—	DNA: tyrosine; DNA/RNA: O–P–O; Proteins: (hydroxy)proline
−850*	+0.1	850 PC1,2	—	Proteins: tyrosine, proline; Carbo: glycogen, polysacch.
−875*[d]	+0.06	875 PC2	875	Proteins: hydroxyproline, tryptoph.; Lipids: choline
**−890[d]**	**+0.6**	**890 PC1,2,7**	**890**	Carbo: saccharide; Proteins; C–C skeletal; methylene
**−934*[d]**	**+0.8**	**934 PC1**	**934**	Proteins: α-helix, proline, valine; Carbo.: C–O-C glycogen
**−968*[f]**	**−0.2**	**968 PC1,2**	—	Lipids C–C (mostly); Phosphorylated proteins and cellular nucleic acids
**+1001*[b,c,d]**	**+3.3**	**1001 PC1,4,7**	**1001**	Proteins: Phenylalanine ring breathing
**−1029**	**+1.0**	**1029 PC1,4**	**1029**	Proteins: C–N & Phenylalanine (C–H); Phospholipids, Carbo.
**−1061***	**−0.2**	**1061 PC2**	—	Lipids: C–C; DNA/RNA: PO$_2^-$ backbone; Carbo. C–O, C–C stretching
**(0)1089[a,b,c]**	**+1.4**	**1089 PC1,2,4,7**	**1089**	DNA: PO$_2^-$ backbone; Lipids: C–C; Proteins: C–N
**(0)1124[f]**	**+1.5**	**1124 PC1,2,4,7**	—	C–C Lipids, Carbo. & Proteins; C–N glucose; C–O carbo.
**−1154[a]**	**+0.5**	**1154 PC2,7**	**1154**	Proteins (C–C, C–N stretch) & Carbo. (glycogen); carotenoids.
**−1171***	**+0.5**	**1171 PC1,7**	**1171**	Proteins: phenylalanine & tyrosine
**−1205*[b]**	**+0.6**	**1205 PC1,2**	—	DNA/RNA & Proteins: tyrosine, phenylalanine, tryptophan, glycine, proline, amide III
**−1233*[b]**	**+1.3**	**1233 PC2**	**1233**	Proteins, DNA/RNA: amide III, glycine, proline, PO$_2^-$
**(0)1253*[b,d]**	**+0.6**	**1253 PC1,2,7**	**1253**	DNA/RNA: guanine & cytosine; Proteins: amide III; Lipids
**(0)1306*[b,d,f]**	**+0.6**	**1306 PC1,2,7**	**1306**	Proteins: C–N amines; DNA/RNA: adenine, cytosine; CH$_2$, CH$_3$/CH$_2$. Lipids
**+1336*[b,f]**	**+1.3**	**1336 PC1,2**	**1336**	Proteins: tryptophan, phenylalanine, amide III, CH deform.; DNA/RNA: A, G; CH$_3$CH$_2$. Lipids (minor)
(0)1399	+0.09	1399 PC2,7	1399	Proteins: C=O, CH$_2$, NH
**−1448*[c,e]**	**−0.6**	**1448 PC2**	—	Lipids & Proteins: CH$_2$, C–H, CH$_2$CH$_3$; DNA/RNA
−1482*[c,e]	+0.1	1482 PC2,7	1482	Proteins: amide II, C-N, N-H; DNA/RNA: guanine, adenine
**+1551[a]**	**+0.1**	**1551 PC7**	—	Proteins: tryptophan, amide II
**+1575*[a,c]**	**+1.1**	**1575 PC1,2**	**1575**	DNA/RNA: nucleic acids, guanine, adenine
**+1603*[a,b,d,e]**	**−0.4**	**1603 PC2,4**	**1603**	Proteins: phenylalanine, tyrosine, C–C, C=C, C–N
**+1615***	**+0.4**	**1615 PC1,2**	**1615**	Proteins: tyrosine, tryptophan C=C
**+1655*[a,b,c,d]**	**+2.7**	**1655 PC1,2,7**	**1655**	Proteins: amide I, α-helix; DNA/RNA: T, G, C bases; Lipids: C=C
**+1680[f]**	**−1.0**	**1680 PC1,2,4**	**1680**	Lipids: C=C cholesterol; Proteins: amide I
**−1745[f]**	**−0.5**	**1745 PC2,7**	**1745**	Lipids: carboxyl group (C=O)

I. PNT2-C2 LIVE VS. DRIED (HIGH WAVENUMBER): KEY SEPARATING BANDS

TABLE S8. Col. 1: $\Delta = +/-$ or 0 average spectral difference per band [Fig. 1(b); SI Figs. S18, S19]. Col. 2: δ_{peak} = Gaussian peak-fitted difference for N(L)–N(D). Col. 3: PCA loadings. Col. 4: PCA-LDA weighted-loadings. Col. 5: peak-assignments. Bands deemed: (i) *significant* in separating the classes where $\delta_{peak} >> $ the sum of the fitted SE uncertainties at 1–6 % SE per fitted band are in **purple**, (ii) $\delta_{peak} >$ the sum of the fitted SE uncertainties are in **teal**, and (iii) $\simeq$ the sum of the fitted SE uncertainties are in plain text. Cf. SI Figs. S12, S17.

(Δ) Band	δ_{peak} (E-4)	PCA loadings	LDA_W	Assignments
**−2726***	**−1.1**	**2726 PC3,4,7**	**2726**	Lipids: C–H stretches
(0)2851*[d,f]	−1.1	2851 PC4,7	2851	Lipids: CH_2.
**(0)2871***	**−1.7**	**2871 PC2,3,4,6,7**	**2871**	Lipids and Proteins: CH_2 & CH_3
**(0)2893***	**+2.1**	**2893 PC2,4,6,7**	**2893**	Lipids & Proteins: CH, CH_2 & CH_3
**+2931***[d,f]	**−9.0**	**2931 PC2,3,4,6,7**	**2931**	Proteins and Lipids: CH_2 & CH_3 (predominantly Proteins)
**+2940***[f]	**+4.7**	**2940 PC2,3,4,7**	**2940**	Proteins and Lipids: C–H; CH_2 modes in Lipids
**+2965***	**+3.1**	**2965 PC2,4,6,7**	**2965**	Lipids: CH_3 asymmetric stretch; cholesterol & cholesterol ester
**+3013***[d,f]	**+0.5**	**3013 PC2,3,4,6,7**	**3013**	Lipids: unsaturated =CH stretch in lipids
**(0)3064***	**+0.5**	**3064 PC2,3,4,6,7**	**3064**	Proteins: CH stretch; phenylalanine, tyrosine & tryptophan

References for Tables S7 and S8

Live versus dead: [a] Notingher et al. 2002 MLE-12 mouse-derived lung cells; [b] Notingher et al. 2003 A549 human lung adenocarcinoma cells; [c] Draux et al. 2010 human, non-small-cell lung cancer cells; **Oxidative stress**: [d] Brozek-Pluska and Beton 2021) CCD-18Co normal colon cell line; [e] Ripanti et al. 2021) Various DNA samples (SERS tested); [f] Machado et al. 2012) Lipids (linoelic acid).

J. P4E6 LIVE VS. DRIED (FINGERPRINT): KEY SEPARATING BANDS

TABLE S9. Col. 1: Δ = +/− or 0 average spectral difference per band [Fig. 1(a); SI Figs. S18, S19]. Col. 2: δ_{peak} = Gaussian peak-fitted difference for C(L)–C(D). Col. 3: PCA loadings. Col. 4: PCA-LDA weighted-loadings. Col. 5: peak-assignments. Bands deemed: (i) *significant* in separating the classes where $\delta_{peak} >> $ the sum of the fitted SE uncertainties at 1–6 % SE per fitted band are in **purple**, (ii) $\delta_{peak} >$ the sum of the fitted SE uncertainties are in **teal**, and (iii) $\simeq$ the sum of the fitted SE uncertainties are in plain text. Cf. SI Figs. S13, S17.

(Δ) Band	δ_{peak} (E-4)	PCA loadings	LDA$_W$	Assignments
**+618***[e]	**+1.1**	**618 PC1,5**	—	Proteins: phenylalanine C–C twist; Lipids (minor): cholesterol ester
**+640**	**+0.1**	**640 PC4**	**640**	Proteins: tyrosine
**+669***	**−0.2**	**669 PC1,3**	**669**	DNA/RNA & Proteins: cystine, tyrosine
+697*	+0.05	697 PC4	697	Proteins: aminoacid methionine (methion.)
**+714**[d]	**+0.6**	**714 PC3**	**714**	Lipids: C–N, $CN^{+/-}(CH_3)_3$; DNA: adenine; Proteins: methion.
**+724***[f]	**+0.3**	**724 PC3,5**	**724**	DNA/RNA: adenine, free adenine
**+745***	**+2.6**	**745 PC1,3,5**	**745**	DNA: thymine
**+756**	**+0.7**	**756 PC3**	**756**	Proteins: tryptophan (tryptoph.)
**+781***[b]	**+0.9**	**781 PC1,3,5**	—	DNA/RNA: uracil, cytosine & thymine; O–P–O DNA
**−807**[d]	**−0.3**	**807 PC3**	**807**	RNA: O–P–O stretch
**−825**[b,d,f]	**+0.7**	**825 PC3**	**825**	DNA: tyrosine; DNA/RNA: O–P–O; Proteins: (hydroxy)proline
**−850***	**+0.8**	**850 PC1**	**850**	Proteins: tyrosine, proline; Carbo: glycogen, polysacch.
**−875**[d]	**−0.2**	—	**875**	Proteins: hydroxyproline, tryptoph.; Lipids: choline
**−890**[d]	**+0.5**	—	—	Carbo: saccharide; Proteins; C–C skeletal; methylene
**(0)934***[d]	**+1.0**	**934 PC1,4,5**	**934**	Proteins: α-helix, proline, valine; Carbo.: C–O-C glycogen
**−968***[f]	**−0.7**	**968 PC1**	**968**	Lipids C–C (mostly); Phosphorylated proteins and cellular nucleic acids
**(0)1001***[b,c,d]	**+2.3**	**1001 PC1,3,4,5**	**1001**	Proteins: Phenylalanine ring breathing
**−1029***	**+1.1**	**1029 PC1,4,5**	**1029**	Proteins: C–N & Phenylalanine (C–H); Phospholipids, Carbo.
**−1061***	**−0.5**	**1061 PC1,4**	**1061**	Lipids: C–C; DNA/RNA: PO_2^- backbone; Carbo. C–O, C–C stretching
**−1089***[a,b,c]	**+1.5**	**1089 PC1,3,4,5**	**1089**	DNA: PO_2^- backbone; Lipids: C–C; Proteins: C–N
−1124*[f]	+0.2	1124 PC1,3,5	1124	C–C Lipids, Carbo. & Proteins; C–N Glucose; C–O Carbo.
**−1154**[a]	**+0.6**	**1154 PC1**	**1154**	Proteins (C–C, C–N stretch) & Carbo. (glycogen); carotenoids.
**−1171**	**+1.0**	**1171 PC3,4**	—	Proteins: phenylalanine & tyrosine
−1205[b]	−0.02	1205 PC3	1205	DNA/RNA & Proteins: tyrosine, phenylalanine, tryptophan, glycine, proline, amide III
**−1233**[b]	**+0.8**	**1233 PC3**	**1233**	Proteins, DNA/RNA: amide III, glycine, proline, PO_2^-
**+1253***[b,d]	**+0.9**	**1253 PC1,3,5**	**1253**	DNA/RNA: guanine & cytosine; Proteins: amide III & lipids
**+1306***[b,d,f]	**+2.0**	**1306 PC3,5**	**1306**	Proteins: C–N amines; DNA/RNA: adenine, cytosine; CH_2, CH_3/CH_2. Lipids
**+1336***[b,f]	**+3.3**	**1336 PC1,3,5**	**1336**	Proteins: tryptophan, phenylalanine, amide III, CH deform.; DNA/RNA: A, G; CH_3CH_2. Lipids (minor)
**+1399***	**+0.3**	**1399 PC1,5**	**1399**	Proteins: C=O, CH2, NH
**−1448***[c,d,e]	**−3.6**	**1448 PC1,3,4,5**	**1448**	Lipids & Proteins: CH_2 , C–H, CH_2CH_3 ; DNA/RNA
**−1482**[c,e]	**−0.3**	**1482 PC3,5**	**1482**	Proteins: amide II, C-N, N-H; DNA/RNA: guanine, adenine
+1551*[a,]	+0.07	1551 PC1,5		Proteins: tryptophan, amide II
**+1575***[a,c,]	**+0.7**	**1575 PC1,3,5**	**1575**	DNA/RNA: nucleic acids, guanine, adenine
**+1603***[a,b,d,e]	**+0.8**	**1603 PC4**	**1603**	Proteins: phenylalanine, tyrosine, C–C, C=C, C–N
**+1615***	**+0.8**	—	**1615**	Proteins: tyrosine, tryptophan C=C
**+1655***[a,b,c,d]	**+1.4**	**1655 PC1,3,4,5**	**1655**	Proteins: amide I, α-helix; DNA/RNA: T, G, C bases; Lipids: C=C
**+1680***[f]	**−1.1**	**1680 PC3,4,5**	**1680**	Lipids: C=C cholesterol; Proteins: amide I
**−1745***[f]	**−0.5**	**1745 PC1,3**	**1745**	Lipids: carboxyl group (C=O)

J. P4E6 LIVE VS. DRIED (HIGH WAVENUMBER): KEY SEPARATING BANDS

TABLE S10. Col. 1: Δ = +/− or 0 average spectral difference per band [Fig. 1(b); SI Figs. S18, S19]. Col. 2: δ_{peak} = Gaussian peak-fitted difference for C(L)–C(D). Col. 3: PCA loadings. Col. 4: PCA-LDA weighted-loadings. Col. 5: peak-assignments. Bands deemed: (i) *significant* in separating the classes where δ_{peak} >> the sum of the fitted SE uncertainties at 1–6 % SE per fitted band are in **purple**, (ii) δ_{peak} > the sum of the fitted SE uncertainties are in **teal**, and (iii) ≃ the sum of the fitted SE uncertainties are in plain text. Cf. SI Figs. S14, S17.

(Δ) Band	δ_{peak} (E-4)	PCA loadings	LDA_W	Assignments
**(0)2726***	**−0.9**	**2726 PC1,4**	—	Lipids: C–H stretches
**−2851***[d,f]	**−6.8**	**2851 PC1,2,3,5**	**2851**	Lipids: CH_2
**−2871***	**−1.5**	**2871 PC1,2,3,4,5**	**2871**	Lipids and Proteins: CH_2 & CH_3
**−2893***	**−4.8**	**2893 PC5**	**2893**	Lipids and Proteins: CH, CH_2 & CH_3
+2931*[d,f]	−0.2	2931 PC2,3,4,5	2931	Proteins and Lipids: CH_2 & CH_3 (predominantly Proteins)
**+2940***[f]	**+8.8**	**2940 PC3,4,5**	**2940**	Proteins and Lipids: C–H; CH_2 modes in Lipids
**+2965***	**+7.5**	**2965 PC2,5**	—	Lipids: CH_3 asymmetric stretch; cholesterol & cholesterol ester
**(0)3013***[d,f]	**+1.3**	**3013 PC1,2,3,4,5**	**3013**	Lipids: unsaturated =CH stretch in lipids
**+3064***	**+1.6**	**3065 PC1,2,3,4**	**3064**	Proteins: CH stretch; phenylalanine, tyrosine & tryptophan

References for Tables S9 and S10

Live versus dead: [a] Notingher et al. 2002 MLE-12 mouse-derived lung cells; [b] Notingher et al. 2003 A549 human lung adenocarcinoma cells; [c] Draux et al. 2010 human, non-small-cell lung cancer cells; **Oxidative stress**: [d] Brozek-Pluska and Beton 2021) CCD-18Co normal colon cell line; [e] Ripanti et al. 2021) Various DNA samples (SERS tested); [f] Machado et al. 2012) Lipids (linoelic acid).

K. P4E6 DRIED VS. PNT2-C2 DRIED (FINGERPRINT): KEY SEPARATING BANDS

TABLE S11. Col. 1: Δ = +/− or 0 average spectral difference per band [Fig. 1(a); SI Figs. S18, S19]. Col. 2: δ_{peak} = Gaussian peak-fitted difference for C(D)–N(D). Col. 3: PCA loadings. Col. 4: PCA-LDA weighted-loadings. Col. 5: peak-assignments. Bands deemed: (i) *significant* in separating the classes where $\delta_{peak} >> $ the sum of the fitted SE uncertainties at 1–6 % SE per fitted band are in **purple**, (ii) $\delta_{peak} >$ the sum of the fitted SE uncertainties are in **teal**, and (iii) $\simeq$ the sum of the fitted SE uncertainties are in plain text. Cf. SI Figs. S15, S17.

(Δ) Band	δ_{peak} (E-4)	PCA loadings	LDA_W	Assignments
(0)618[a]	+0.01	618	—	Proteins: phenylalanine C–C twist; Lipids (minor): cholesterol ester
**(0)640[a]**	**+0.3**	**640 PC3,4,6**	—	Proteins: tyrosine
**(0)669**	**+0.8**	**669**	—	DNA/RNA & Proteins: cystine, tyrosine
**−697**	**+0.7**	—	**697**	Proteins: aminoacid methionine (methion.)
**−714[b]**	**−0.9**	**714 PC2,6**	**714**	Lipids: C–N, $CN^{+/-}(CH_3)_3$; DNA: adenine; Proteins: methion.
**(0)724[a,c]**	**+0.3**	**724 PC4,6**	**724**	DNA/RNA: adenine, free adenine
**−745***	**−0.4**	**745 PC2,3,6**	**745**	DNA: thymine
**−756**	**−0.2**	**756 PC2,3**	**756**	Proteins: tryptophan (tryptoph.)
+781*[a,c]	+0.1	781 PC2,3,4,6	—	DNA/RNA: uracil, cytosine & thymine; O–P–O DNA
**+807[b]**	**+0.4**	**807 PC4,6**	—	RNA: O–P–O stretch
**(0)825[c]**	**−0.5**	**825 PC4,6**	—	DNA: tyrosine; DNA/RNA: O–P–O; Proteins: (hydroxy)proline
+850*[a]	+0.2	850 PC3,4,6	850	Proteins: tyrosine, proline; Carbo: glycogen, polysacch.
**−875**	**+0.2**	**875 PC2,3,4**	—	Proteins: hydroxyproline, tryptoph.; Lipids: choline
**−890**	**+0.3**	**890 PC2,4**	—	Carbo: saccharide; Proteins; C–C skeletal; methylene
−934*[c]	−0.2	934 PC2,3,4	934	Proteins: α-helix, proline, valine; Carbo.: C–O-C glycogen
**−968***	**−1.0**	**968 PC2,3,4**	**968**	Lipids C–C (mostly); Phosphorylated proteins and cellular nucleic acids
**−1001*[a,b,e]**	**+2.1**	**1001 PC3,4,6**	**1001**	Proteins: Phenylalanine ring breathing
+1029*[a]	+0.1	1029 PC3,4,6	1029	Proteins: C–N & Phenylalanine (C–H); Phospholipids, Carbo.
**+1061***	**+0.4**	**1061 PC2**	**1061**	Lipids: C–C; DNA/RNA: PO_2^- backbone; Carbo. C–O, C–C stretching
**+1089*[b,c]**	**−0.2**	**1089 PC2,3,6**	—	DNA: PO_2^- backbone; Lipids: C–C; Proteins: C–N
**+1124*[a,d]**	**+0.9**	**1124 PC3,6**	**1124**	C–C Lipids, Carbo. & Proteins; C–N Glucose; C–O Carbo.
**−1154**	**+0.3**	**1154 PC4,6**	—	Proteins (C–C, C–N stretch) & Carbo. (glycogen); carotenoids
**−1171**	**−0.4**	**1171 PC3,6**	—	Proteins: phenylalanine & tyrosine
**(0)1205[a]**	**+0.7**	**1205 PC6**	—	DNA/RNA & Proteins: tyrosine, phenylalanine, tryptophan, glycine, proline, amide III
(0)1233[a]	+0.02	—	—	Proteins, DNA/RNA: amide III, glycine, proline, PO_2^-
**−1253*[c,d,e]**	**−0.7**	**1253 PC3,4,6**	**1253**	DNA/RNA: guanine & cytosine; Proteins: amide III & Lipids
**(0)1306*[c,d,e]**	**−0.5**	**1306 PC3,6**	**1306**	Proteins: C–N amines; DNA/RNA: adenine, cytosine; CH_2, CH_3/CH_2. Lipids
+1336*[e]	−0.1	1336 PC2,3,4	—	Proteins: tryptophan, phenylalanine, amide III, CH deform.; DNA/RNA: A, G. CH_3CH_2. Lipids (minor)
(0)1399*[d]	−0.08	1399 PC3,4	1399	Proteins: C=O, CH_2, NH
**+1448*[a,c,d,e]**	**+2.5**	**1448 PC2,3**	**1448**	Lipids & Proteins: CH_2, C–H, CH_2CH_3; DNA/RNA
**+1482**	**+0.5**	**1482 PC2,6**	**1482**	Proteins: amide II, C-N, N-H; DNA/RNA: guanine, adenine
−1551*	+0.02	1551 PC2,3,4,6	1551	Proteins: tryptophan, amide II
**(0)1575*[b,c]**	**+0.4**	**1575 PC2,4,6**	—	DNA/RNA: nucleic acids, guanine, adenine
**−1603*[a]**	**−0.6**	**1603 PC2,3,6**	—	Proteins: phenylalanine, tyrosine, C–C, C=C, C–N
(0)1615*[a,d]	−0.03	1615 PC3,6	—	Proteins: tyrosine, tryptophan C=C
**+1655*[b,c]**	**+1.0**	**1655 PC2,3,6**	—	Proteins: amide I, α-helix; DNA/RNA: T, G, C bases; Lipids: C=C
**+1680***	**+0.4**	**1680 PC2,3,4,6**	**1680**	Lipids: C=C cholesterol; Proteins: amide I
(0)1745*	~0	1745 PC3,4,6	—	Lipids: carboxyl group (C=O)

K. P4E6 DRIED VS. PNT2-C2 DRIED (HIGH WAVENUMBER): KEY SEPARATING BANDS

TABLE S12. Col. 1: Δ = +/− or 0 average spectral difference per band [Fig. 1(b); SI Figs. S18, S19]. Col. 2: δ_{peak} = Gaussian peak-fitted difference for C(D)–N(D). Col. 3: PCA loadings. Col. 4: PCA-LDA weighted-loadings. Col. 5: peak-assignments. Bands deemed: (i) *significant* in separating the classes where $\delta_{peak} >> $ the sum of the fitted SE uncertainties at 1–6 % SE per fitted band are in **purple**, (ii) $\delta_{peak} >$ the sum of the fitted SE uncertainties are in **teal**, and (iii) ≃ the sum of the fitted SE uncertainties are in plain text. Cf. SI Figs. S16, S17.

(Δ) Band	δ_{peak} (E-4)	PCA loadings	LDA_W	Assignments
**−2726***	**−0.6**	**2726 PC1,3**	**2726**	Lipids: C–H stretches
**+2851***[a,e,f]	**+0.9**	**2851 PC1,3,4,7**	**2851**	Lipids: CH_2
+2871*	−0.6	2871 PC1,3,4,6,7	2871	Lipids and Proteins: CH_2 & CH_3
+2893*[e,f]	+3.4	2893 PC1,3,4,6,7	2893	Lipids and Proteins: CH, CH_2 & CH_3
**−2931***	**−8.1**	**2931 PC3,6,7**	**2931**	Proteins and Lipids: CH_2 & CH_3 (predominantly proteins)
**−2940***[a,f]	**−3.3**	**2940 PC3**	**2940**	Proteins and Lipids: C–H lipids & proteins; CH_2 modes in lipids
**−2965***[a,e,f]	**−1.6**	**2965 PC3,6,7**	**2965**	Lipids: CH_3 asymmetric stretch; cholesterol & cholesterol ester
**−3013***[a,e,f]	**−1.4**	**3013 PC1,3,4,6,7**	**3013**	Lipids: unsaturated =CH stretch in lipids
(0)3064*	∼0	3064 PC1,3,4,7	3064	Proteins: CH stretch; phenylalanine, tyrosine & tryptophan

References for Tables S11 and S12

Prostate cancer vs. normal equivalent or treated:

[a] Corsetti et al. 2018 DU145 (metastatic prostate cancer) vs. PNT2-C2 (normal prostate) cell lines. Ethanol fixed.

[b] Taleb et al. 2006 LNCaP (metastatic prostate cancer) vs. PNT1A (normal) cell lines. Ethanol fixed.

[c] Crow et al. 2005 LNCaP, PCa 2b (AR-positive) and PC 3, DU145 (AR-negative) metastatic cell lines. Cells kept “moist”.

[d] van Breugel et al. 2023 Prostate tissues from biopsy. Benign, Gleason 3, 4 and 5 comparisons. Formalin fixed.

[e] Potcoava et al. 2014 LNCaP and PC3 (metastatic cell lines). Untreated versus treated. Formaldehyde fixed.

[f] Hislop et al. 2022 PC3 and LNCaP versus PNT2 normal prostate cell line. Cells fixed with paraformaldehyde.

M. REPRESENTATIVE PEAK ASSIGNMENTS FROM THE LITERATURE

TABLE S13. Representative peak assignments from the literature relating to Raman studies on prostate-cancer cells and tissues that have normal or treated cell line equivalents. Cf. Tables I, II, III, also Tables S5, S6, S11, S12. Peak assignments in bold are significantly discriminant in the P4E6 vs. PNT2-C2 live-cell comparison. Those with * are significantly discriminant in the live vs. dried disease-state comparison.

System	Assignments
DU145 metastatic prostate cancer vs. PNT2-C2 normal equivalent cell lines; ethanol fixed (Corsetti et al. 2018)	phenylalanine (**621***, **1003***, **1031** and 1208 cm^{-1}); tyrosine (643* and **853*** cm^{-1}); DNA at **723**, 798[†] cm^{-1}; **1126*** cm^{-1} CN stretching modes in proteins and vibrational modes of CO in carbohydrates; protein content **1447*** cm^{-1}; lipids (phosphatidylinositol) at 415[†] cm^{-1}; protein denaturing agent (thiocynate) at 445[†] cm^{-1}; glycogen (bands at 485[†], 490[†] cm^{-1}); 497[†] cm^{-1} amino acid (L-arginine); nucleic acids at 1184[†] cm^{-1}
Metastatic prostate cancers: LNCaP, PCA 2b (AR-pos.) and PC 3, DU145 (AR-neg.); CaF_2 slides. All-against-all. Cells "kept in a moist environment" (Crow et al. 2005)	glycogen (484[†] cm^{-1}); nucleic acids (**721***, **783–786**, **1305***, 1381[†] , **1450*** and 1576–1577* cm^{-1}); DNA backbone (O–P–O) (827* and 1096[†] cm^{-1}); disordered proteins (**1250** and 1658* cm^{-1}); α-helix proteins (935, 1263[†] and 1657* cm^{-1}); phospholipids (**719**, 1094, **1125*** and 1317[†] cm^{-1}); lipids (1090, **1302*** and 1373[†] cm^{-1})
LNCaP (metastatic prostate cancer) vs. PNT1A (normal equivalent) cell lines; ethanol fixed (Taleb et al. 2006)	**706–711**, 807–813*, 1099–1100[†], 1243[†], and 1572* cm^{-1} typical of A-DNA/RNA; 733[†], **790–798**, 835[†], & 1091–1092 cm^{-1} typical of B-DNA/RNA; 1650–1658* and 1668[†] amide I $^{-1}$; 1242[†] and 1265–1274[†] amide III cm^{-1}; phenylalanine at **1003*** cm^{-1} (indicated subtraction spectra & PCA, but not mentioned).
Prostate tissues from biopsy. Benign, Gleason 3, 4 and 5 comparisons. Formalin fixed. (van Breugel et al. 2022)	**881*** cm^{-1} CH_2 proteins (tryptophan); **1307*** cm^{-1} CH_2/CH_3 proteins, lipids; **1310*** cm^{-1} CH_2 proteins; 1396* cm^{-1} CH_2 lipids; 1583[†] cm^{-1} C=C proteins (phenylalanine); **1602*** cm^{-1} C=C proteins (phenylalanine, tyrosine, tryptophan); **1250** cm^{-1} & **1450*** cm^{-1} (indicated in comparison spectra, but not mentioned).
Lipid papers:<br>(i) Potcoava et al. 2014: LNCaP and PC3 metastatic cell lines. Untreated vs. treated<br><br>(ii) Hislop et al. 2022: PC3 and LNCaP versus PNT2 normal prostate cell line	 **1002*** cm^{-1}, 1263[†] cm^{-1}, 1294[†] cm^{-1}, 1343[†] cm^{-1}, **1440*** cm^{-1}, **2851*** cm^{-1}, 2873 cm^{-1}, 2930* cm^{-1}, **2959*** cm^{-1}, **3015*** cm^{-1} [see also Figs. 2(c) and (d) in their paper for comparisons between these points].<br>2930* cm^{-1} CH_3 symmetric stretch in proteins; **2851*** cm^{-1} CH_2 symmetric stretch; **2965*** cm^{-1} cholesteryl esters; **3015*** cm^{-1} =CH, unsaturated lipids.
Other comparative component spectra	Prescott et al. 1984: A-form DNA: **704***, 1396, 1418[†], 2950[†] cm^{-1}; B-form DNA: 834[†], 923[†], 932, 1292[†], 1422[†], **1445*** cm^{-1}

[†] band assignments not resolved / measured in this study.

Blue: Band assignments found in P4E6 vs. PNT2-C2 live cell comparison, but not deemed *significant*.

Blue bold: band assignments found in P4E6 vs. PNT2-C2 live cell comparison deemed *significant*.

* band assignments found in the dried-state comparison deemed *significant*.

TABLE S14. Representative peak assignments from the literature relating to live versus dead cell studies. Peak assignments highlighted in bold plus * correspond to bands with near-enough equivalence to those determined to be relevant for drying effects. Cf. Figs. II, III. See also Tables S7, S8, S9, S10.

System	Assignments
MLE-12 mouse-derived lung cells—live versus dead (Notingher et al. 2002)	**1070–1150*** cm^{-1}, **1530–1700*** cm^{-1}; 1094 cm^{-1} DNA; dead cells had intense bands at **1578*** cm^{-1} and & **1607*** cm^{-1}, plus a new band at 1114† cm^{-1}.
A549 human lung adenocarcinoma cells—live versus dead (Notingher et al. 2003)	782, 788, **828*** cm^{-1} DNA; **1005*** cm^{-1} phenylalanine; 1095 cm^{-1} lipids, DNA; **1231*** cm^{-1} protein; 1320† cm^{-1} DNA/RNA, proteins; 1342 cm^{-1} DNA/RNA, carbohydrates, glucose (**1300–1360*** cm^{-1}, and **1190–1385*** cm^{-1} region, in general); **1607*** cm^{-1}, **1660*** cm^{-1} amide I; 1742 cm^{-1} (appears in the dead cells).
Calu-1 human non-small-cell lung cancer—live versus air dried (Draux et al. 2010)	No statistical significance in difference across the DNA bands, only in RNA bands at 787, 1092, **1486***, **1575*** cm^{-1} and **1003***, 1264†, **1451***, **1660*** cm^{-1} differences in proteins.

† band assignments not resolved in this study

TABLE S15. Representative peak assignments from the literature relating to oxidative stress studies. Peak assignments highlighted in bold plus * correspond to bands with near-enough equivalence to those determined to be relevant for drying effects. Cf. Figs. II, III. See also Tables S7, S8, S9, S10.

System	Assignments
CCD-18Co normal colon cell line (paraformaldehyde fixed). Oxidative damage via *t*BHP addition (Brozek-Pluska and Beton 2021)	Reductions in the oxidative-damaged samples occur in the following bands relative to the control: **716*** cm^{-1} DNA (adenine)/lipids; **812*** cm^{-1} tyrosine (amino acid); **820*** cm^{-1} proteins; 832† cm^{-1} tyrosine (amino acid), DNA; 869† cm^{-1} proteins; 891 cm^{-1} proteins; 932 cm^{-1} DNA, proline, collagen backbone; **1004*** cm^{-1} phenylalanine; 1078† cm^{-1} DNA; 1254 cm^{-1} Amide III / DNA; **1304*** cm^{-1} DNA, lipids; **1444*** cm^{-1} lipids; **1602*** cm^{-1} phenylalanine; 1626† cm^{-1} amide; **1654*** cm^{-1} amide I; 1720† cm^{-1} C=O; 1754 cm^{-1} C=O (lipid); **2854*** cm^{-1} lipids and proteins; 2880† cm^{-1} lipids and proteins; **2926*** cm^{-1} primarily protein; **3009*** cm^{-1} lipids. PIR markers for oxidative stress with most discriminatory change: 812/832; 869/932; 832/1078; 1004/1254; 1078/1388; 1254/1656; 2854/3009.
Lipids (linoelic acid)—oxidative damage through heating (Machado et al. 2012)	**726***, **819***, 841†, 910†, 973, **1118***, 1165†, 1190†, 1266†, 1297†, **1308***, **1333***, **1597***, 1635†, 1642†, 1672†, 1695†, 1737, **2855***, 2907†, **2926***, **2944***, 2983†, **3013*** cm^{-1} lipid bands (various Raman-active modes).
Across various DNA samples (SERS tested)—oxidative damage (Ripanti et al. 2021)	**1400–1600*** cm^{-1} region in general; 8-Oxo-dGTP/dGTP: **1445*** DNA/RNA, 1535† cm^{-1} DNA-oxidised (guanine base)+ PIR of this; **1607*** cm^{-1} DNA-oxidised (guanine base) + PIR of this; 8-Oxo-dATP/dATP: **620*** cm^{-1} + PIR of this.

† band assignments not resolved in this study

II. SUPPLEMENTARY FIGURES

A. TRYPAN BLUE ASSAY DEMONSTRATING LIVE-CELL RAMAN

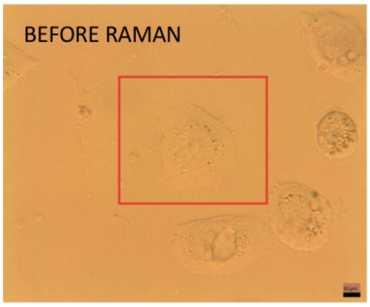

(a)

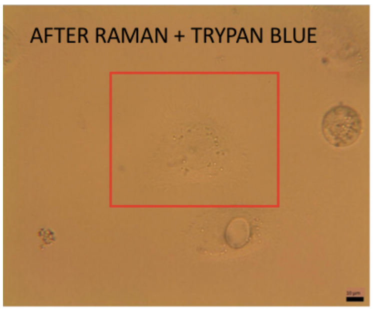

(b)

FIG. S1. Trypan-blue assay viability test of live P4E6 cells after Raman measurement on the central cell (red box) using the spectral acquisition parameters described in the Methods section (‘Raman spectroscopy measurements’). (a) Shows the cell before Raman acquisition. (b) Shows the same cell after Raman acquisition following trypan-blue application. The lack of cellular trypan-blue uptake in (b) demonstrates cell viability post-measurement, and therefore non-destructive Raman testing. Scale-bar = 10μm.

B. STATISTICAL CONVERGENCE TESTS: PNT2-C2 LIVE

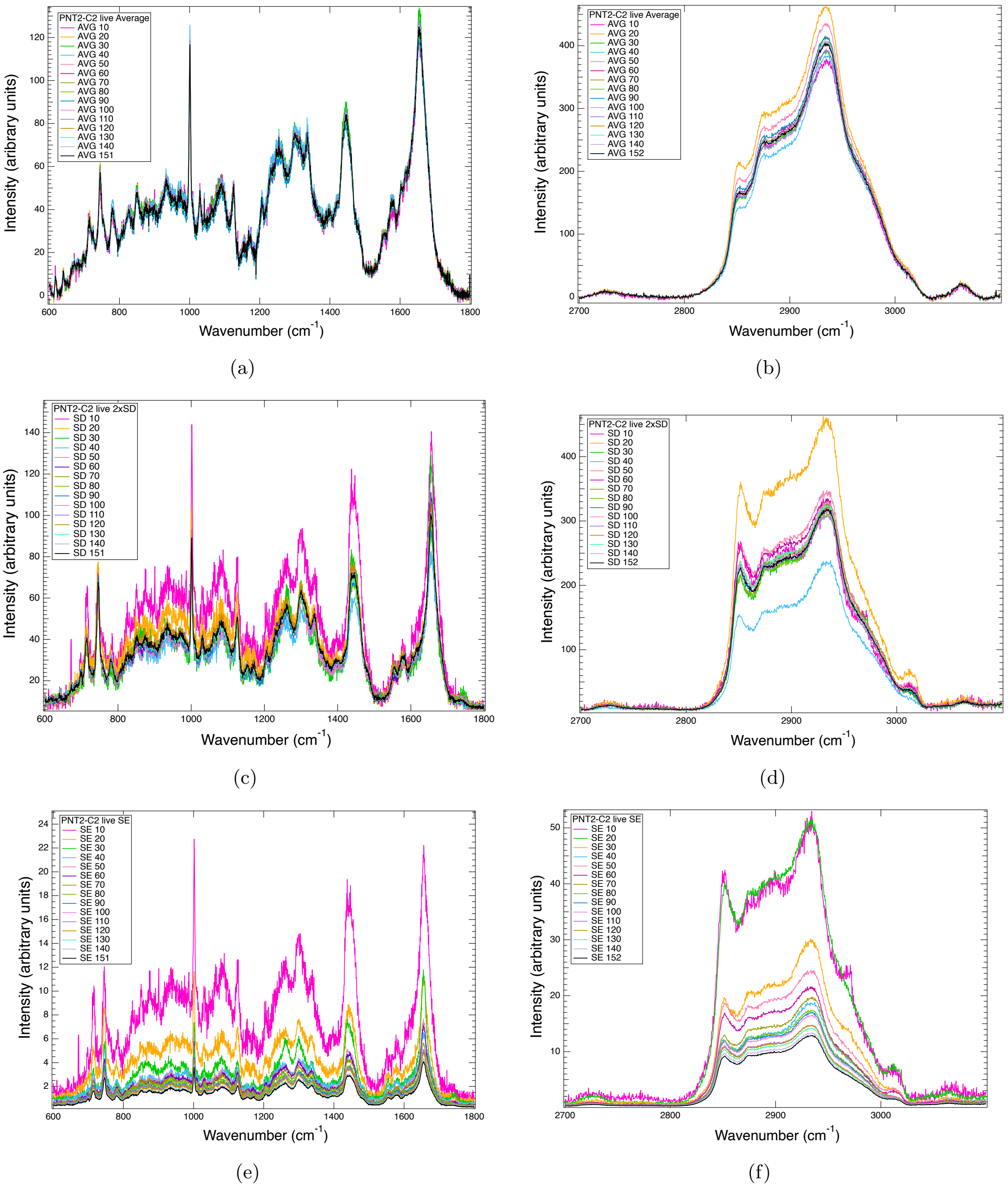

FIG. S2. Convergence of the (a) & (b) spectral average, (c) & (d) twice the standard deviation (2xSD), and (e) & (f) standard error of the mean (SE) as a function of the increasing number of spectra per spectral average for the PNT2-C2 live-cell fingerprint, and high-wavenumber regions (population-level). Each spectrum corresponds to a single-cell Raman measurement taken at random across the cell population.

B. STATISTICAL CONVERGENCE TESTS (CONT'D): P4E6 LIVE

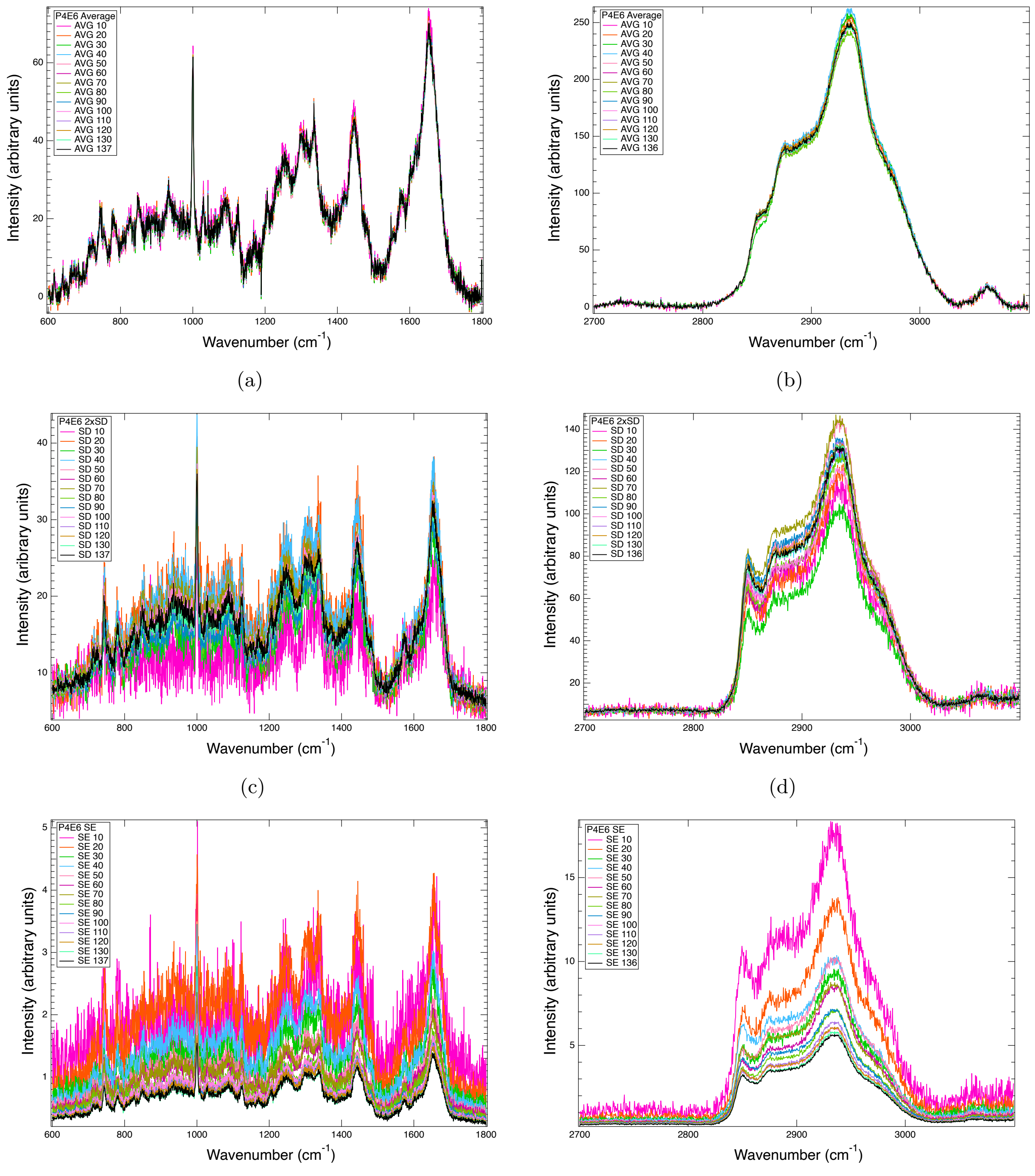

(a) (b)

(c) (d)

(e) (f)

FIG. S3. Convergence of the (a) & (b) spectral average, (c) & (d) twice the standard deviation (2xSD), and (e) & (f) standard error of the mean (SE) as a function of the increasing number of spectra per spectral average for the P4E6 live-cell fingerprint, and high-wavenumber regions (population-level). Each spectrum corresponds to a single-cell Raman measurement taken at random across the cell population.

B. STATISTICAL CONVERGENCE TESTS (CONT'D): PNT2-C2 DRIED

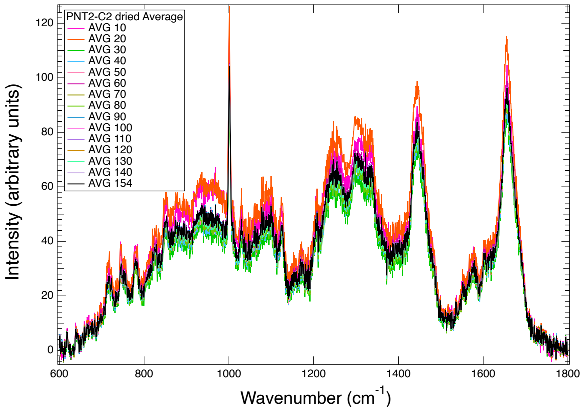

(a)

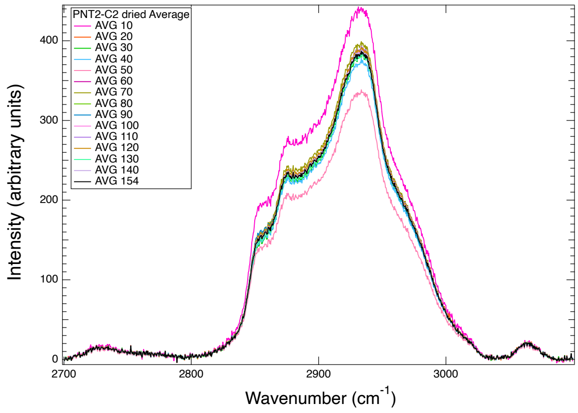

(b)

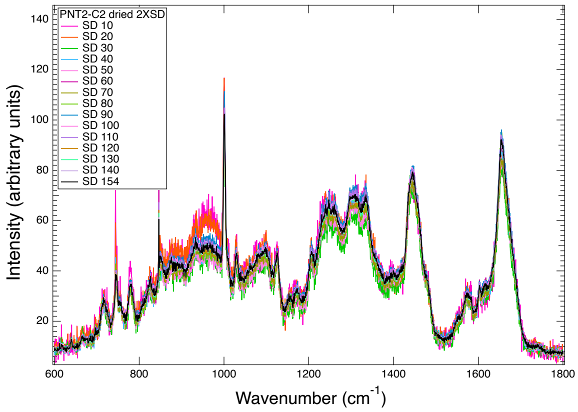

(c)

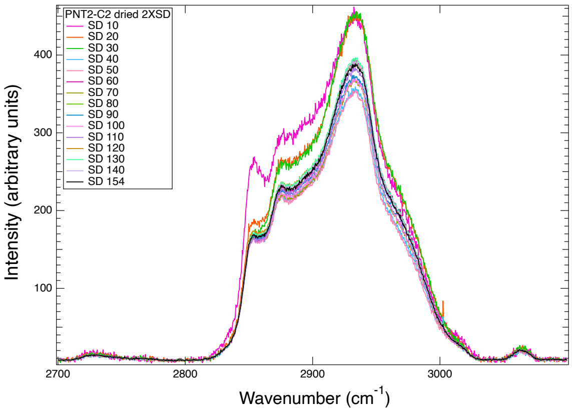

(d)

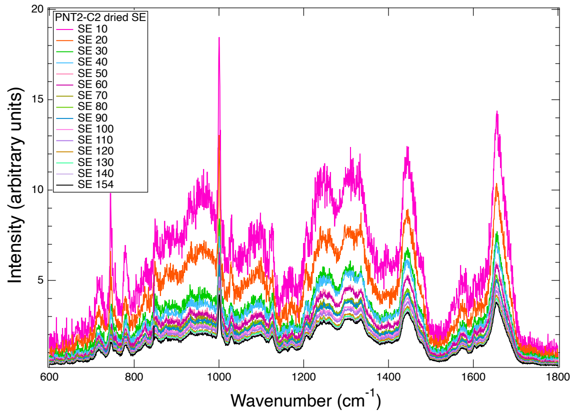

(e)

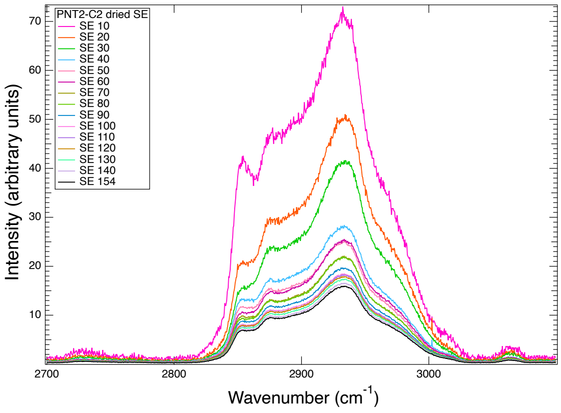

(f)

FIG. S4. Convergence of the (a) & (b) spectral average, (c) & (d) twice the standard deviation (2xSD), and (e) & (f) standard error of the mean (SE) as a function of the increasing number of spectra per spectral average for the PNT2-C2 dried-cell fingerprint, and high-wavenumber regions (population-level). Each spectrum corresponds to a single-cell Raman measurement taken at random across the cell population.

B. STATISTICAL CONVERGENCE TESTS (CONT'D): P4E6 DRIED

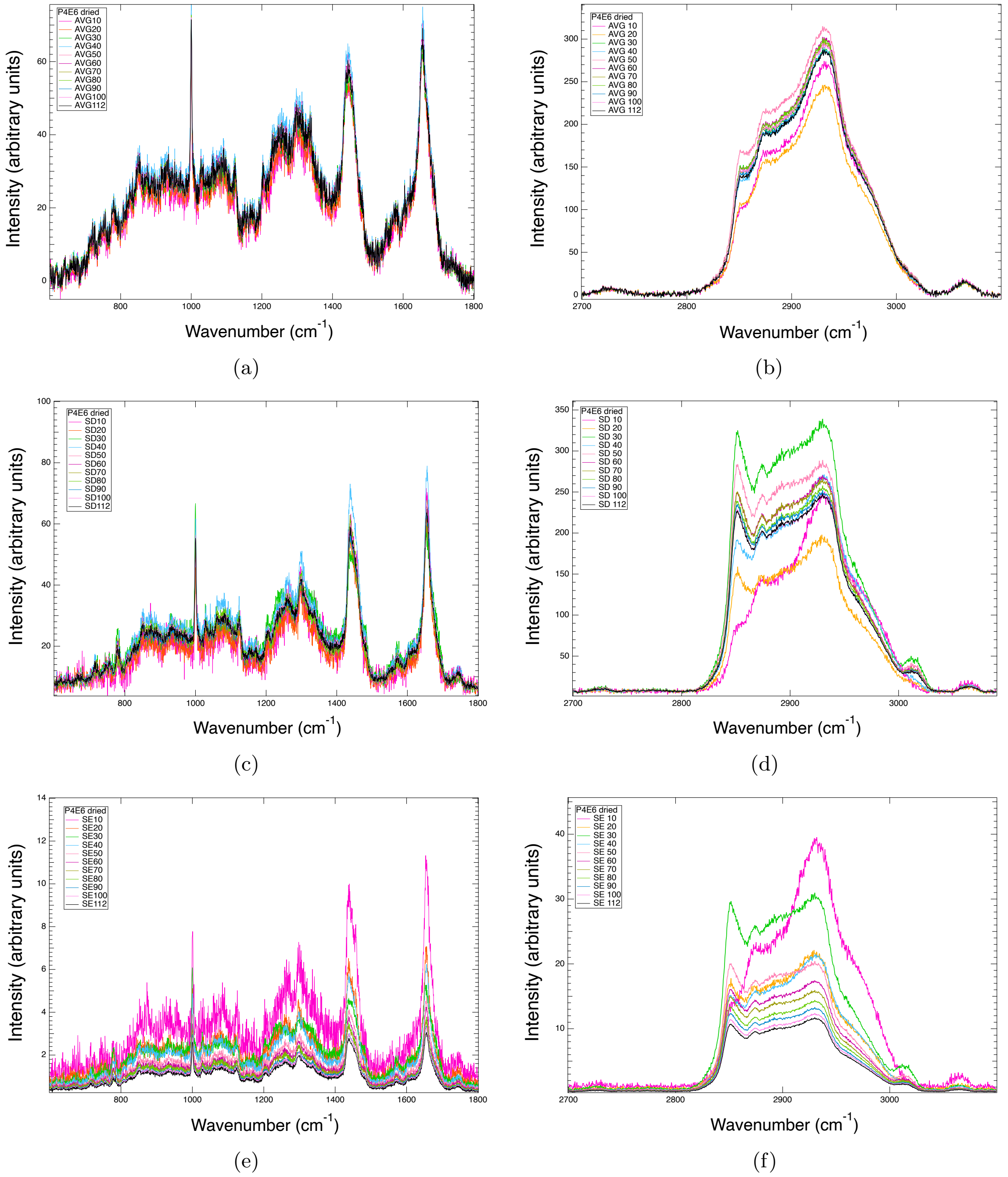

FIG. S5. Convergence of the (a) & (b) spectral average, (c) & (d) twice the standard deviation (2xSD), and (e) & (f) standard error of the mean (SE) as a function of the increasing number of spectra per spectral average for the P4E6 dried-cell fingerprint, and high-wavenumber regions (population-level). Each spectrum corresponds to a single-cell Raman measurement taken at random across the cell population.

B. AVERAGE, STATISTICALLY-CONVERGED, SPECTRA–BASELINED ONLY

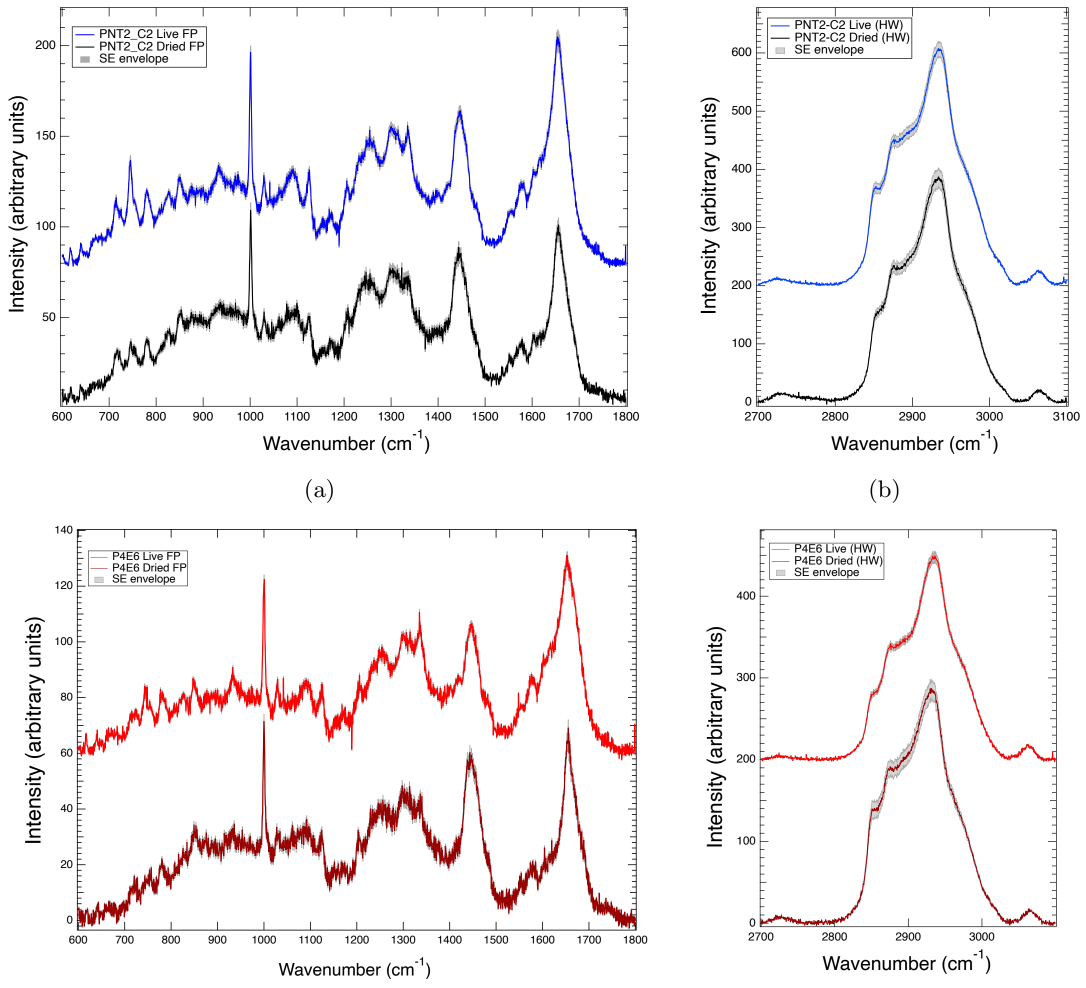

(a) (b) (c) (d)

FIG. S6. Averaged baselined-only, non-normalised spectra with statistically-converged standard error (SE) envelopes for: PNT2-C2 (a) fingerprint (FP) region (N=151 live spectra and N=154 dried spectra averaged), and (b) high wavenumber (HW) region (N=152 live spectra and N=154 dried spectra averaged), P4E6 (c) fingerprint (FP) region (N=137 live spectra and N=112 dried spectra averaged), and (d) high wavenumber (HW) region (N=136 live spectra and N=112 dried spectra averaged). Spectral processing has involved minimum linear, end-to-end baseline subtraction in the fingerprint region. For the live-cell HW region, a cubic spline background was removed due to the HBSS (buffer) background. For the dried-cell HW region, a linear, end-to-end baseline was removed.

C. %-VARIANCE FOR EACH PC, AND CUMULATIVE %-VARIANCE—FP REGION

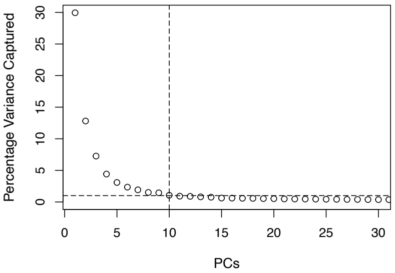

(a) %-variance. $PC_{LDA} = 7$. PNT2-C2 live vs. dried state FP.

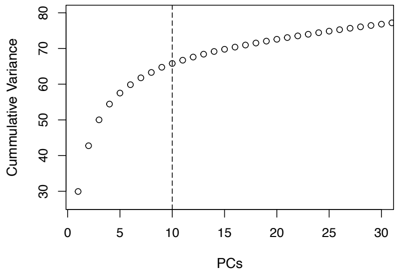

(b) Cumulative %-variance. PNT2-C2 live vs. dried state FP

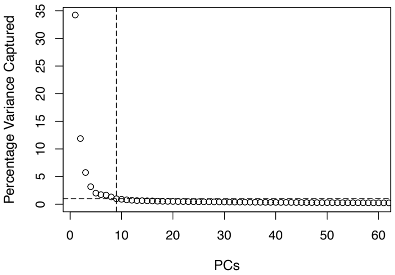

(c) %-variance. $PC_{LDA} = 6$. P4E6 live vs. dried state FP

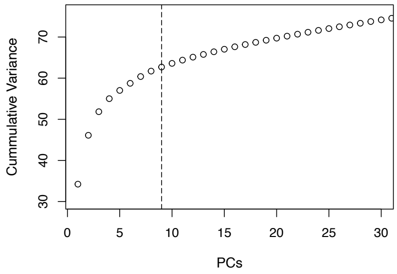

(d) Cumulative %-variance. P4E6 live vs. dried state FP

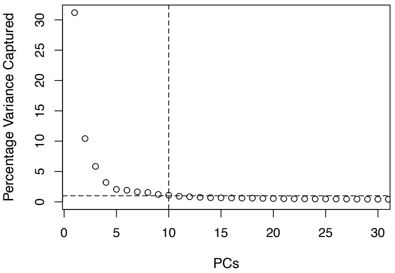

(e) %-variance. $PC_{LDA} = 10$. PNT2-C2 vs. P4E6 live state FP

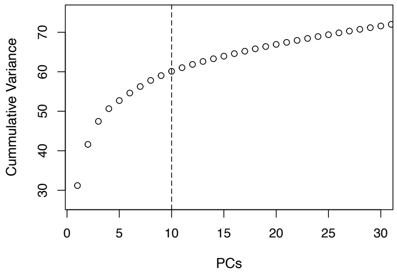

(f) Cumulative %-variance. PNT2-C2 vs. P4E6 live state FP

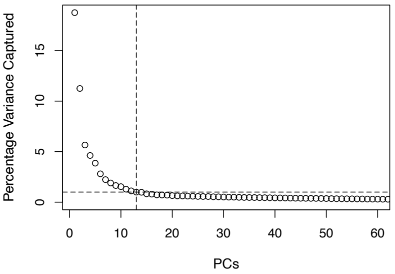

(g) %-variance. $PC_{LDA} = 10$. PNT2-C2 vs. P4E6 dried state FP

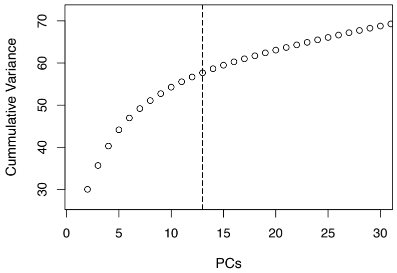

(h) Cumulative %-variance. PNT2-C2 vs. P4E6 dried state FP

FIG. S7. Elbow plots showing the %-variance for each PC [(a), (c), (e), and (g)], and cumulated %-variance for increasing numbers of PCs [(b), (d), (f), and (h)] in the fingerprint (FP) region. The vertical line defines the Kaiser criterion point, at or below which all PCs have > 1% variance. Here, PC_{LDA} is the chosen number of PCs for the LDA calculation as per the stability checks for the %-accuracy of the LOOV (cf. Table S4).

C. %-VARIANCE FOR EACH PC, AND CUMULATIVE %-VARIANCE—HW REGION

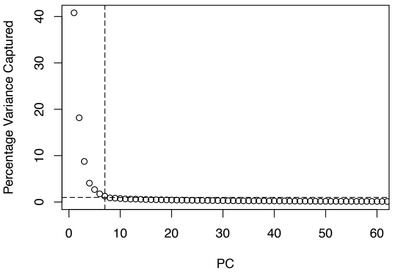

(a) %-variance. $PC_{LDA} = 7$.
PNT2-C2 live vs. dried state HW

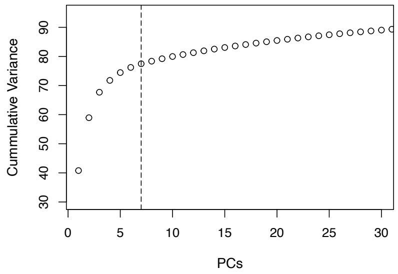

(b) Cumulated %-variance.
PNT2-C2 live vs. dried state HW

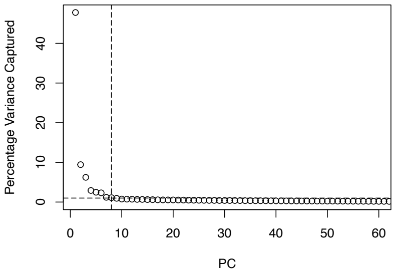

(c) %-variance. $PC_{LDA} = 7$.
P4E6 live vs. dried state HW

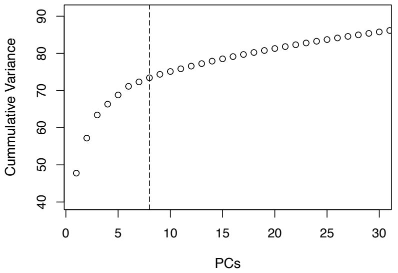

(d) Cumulated %-variance.
P4E6 live vs. dried state HW

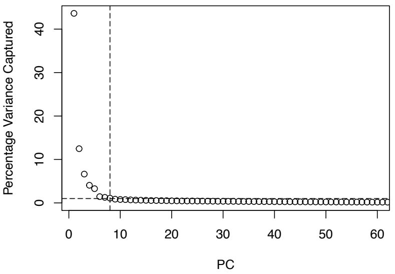

(e) %-variance. $PC_{LDA} = 15$.
PNT2-C2 vs. P4E6 live state HW

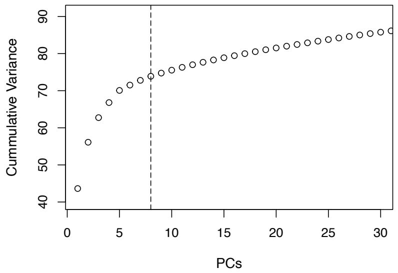

(f) Cumulated %-variance.
PNT2-C2 vs. P4E6 live state HW

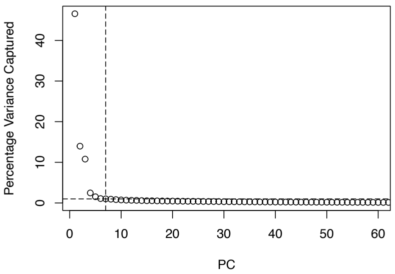

(g) %-variance. $PC_{LDA} = 7$.
PNT2-C2 vs. P4E6 dried state HW

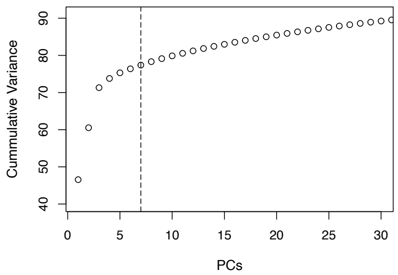

(h) Cumulated %-variance.
PNT2-C2 vs. P4E6 dried state HW

FIG. S8. Elbow plots for the comparison cases listed showing the %-variance for each PC [(a), (c), (e), and (g)], and cumulated %-variance for increasing numbers of PCs [(b), (d), (f), and (h)] in the high-wavenumber (HW) region. The vertical line defines the Kaiser criterion point, at or below which, all PCs have $> 1\%$ variance. Here, PC_{LDA} is the chosen number of PCs for the LDA calculation as per the stability checks for the %-accuracy of the LOOV (cf. Table S4).

D. PCA AND PCA-LDA—PNT2-C2 LIVE VS. P4E6 LIVE (FINGERPRINT)

PC	%-var.	PCA grp. mean sep.	Ratio btw-to-within grp. var.	LDF co-eff
4	3	8.1	0.63	-0.144
1	31	24.6	0.57	0.045
7	2	4.7	0.35	0.161
8	2	3.0	0.14	-0.111
3	6	5.3	0.12	0.052
5	2	2.4	0.06	-0.065
6	2	1.6	0.03	-0.046
10	1	0.6	0.01	-0.031
9	1	0.6	0.01	0.027
2	10	0.1	0.00	0.000

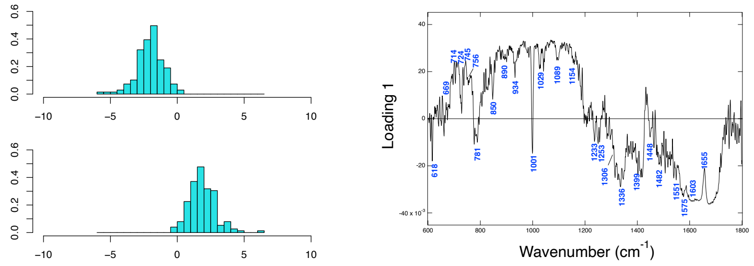

(a) (b) (c)

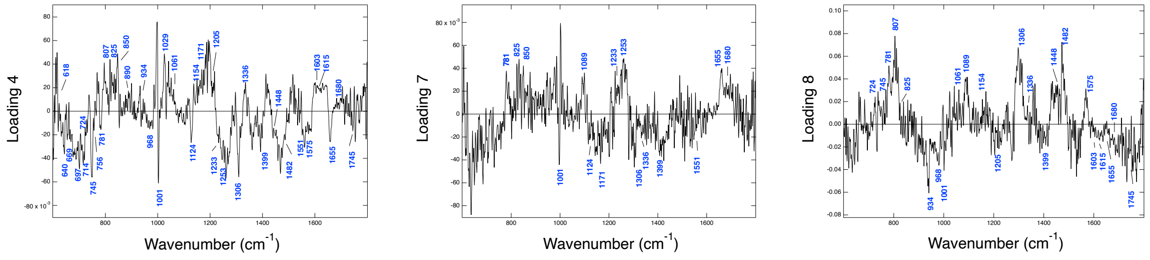

(d) (e) (f)

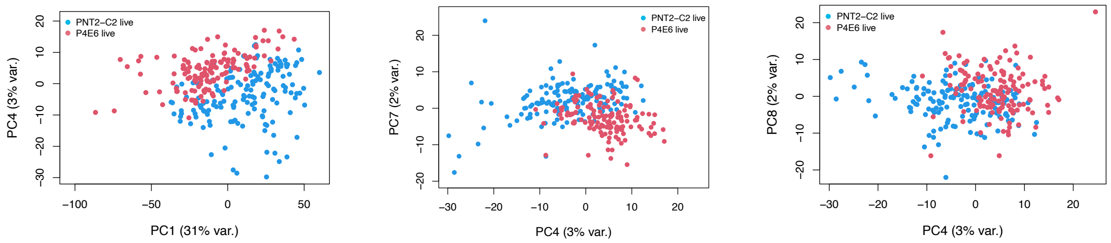

(g) (h) (i)

FIG. S9. (a) Table showing the PCs, %-variance captured, PCA group-mean separations, ratio of the between-to-within group variances, and LDF coefficients for the PNT2-C2 live versus P4E6 live comparison in the fingerprint region. The table is ordered according to column 4. (b) PCA-LDA histogram results taken over the first seven contributing PCs as per the table in (a). Converged LOOV = 98% separation of classes with 10 PCs (60% variance captured) (cf. Table S4). PCs 4, 7, and 8 with highest-weighted LDF-coefficients contribute maximally to the histogram group-separation. (c), (d), (e), and (f) show PC1 (highest %-variance), and PC4, 7 and 8 loadings, respectively, with key wavenumbers labelled. (g), (h), and (i) are PCA plots showing the maximally separated groups.

D. PCA AND PCA-LDA—PNT2-C2 LIVE VS. P4E6 LIVE (HIGH WAVENUMBER)

PC	%-var.	PCA grp. mean sep.	Ratio btw-to-within grp. var.	LDF co-eff
2	12	17.0	2.36	-0.194
1	44	14.6	0.27	0.048
4	4	4.0	0.21	0.140
8	1	1.4	0.08	0.179
5	3	1.9	0.06	0.084
7	1	1.0	0.04	0.110
12	1	0.5	0.01	-0.094
15	1	0.4	0.01	-0.102
3	7	1.3	0.01	-0.028
6	1	0.5	0.01	-0.045
14	1	0.3	0.01	-0.061
10	1	0.3	0.00	0.051
9	1	0.3	0.00	-0.049
13	1	0.1	0.00	-0.028
11	1	0.1	0.00	0.010

(a)

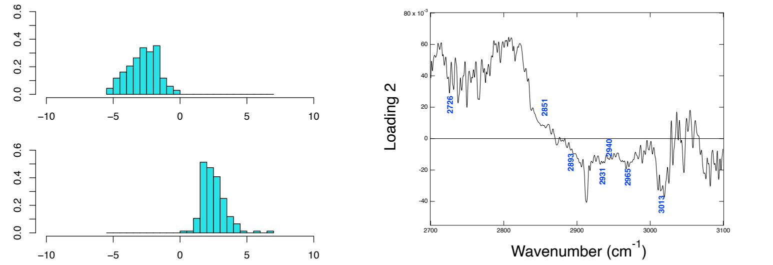

(b) (c)

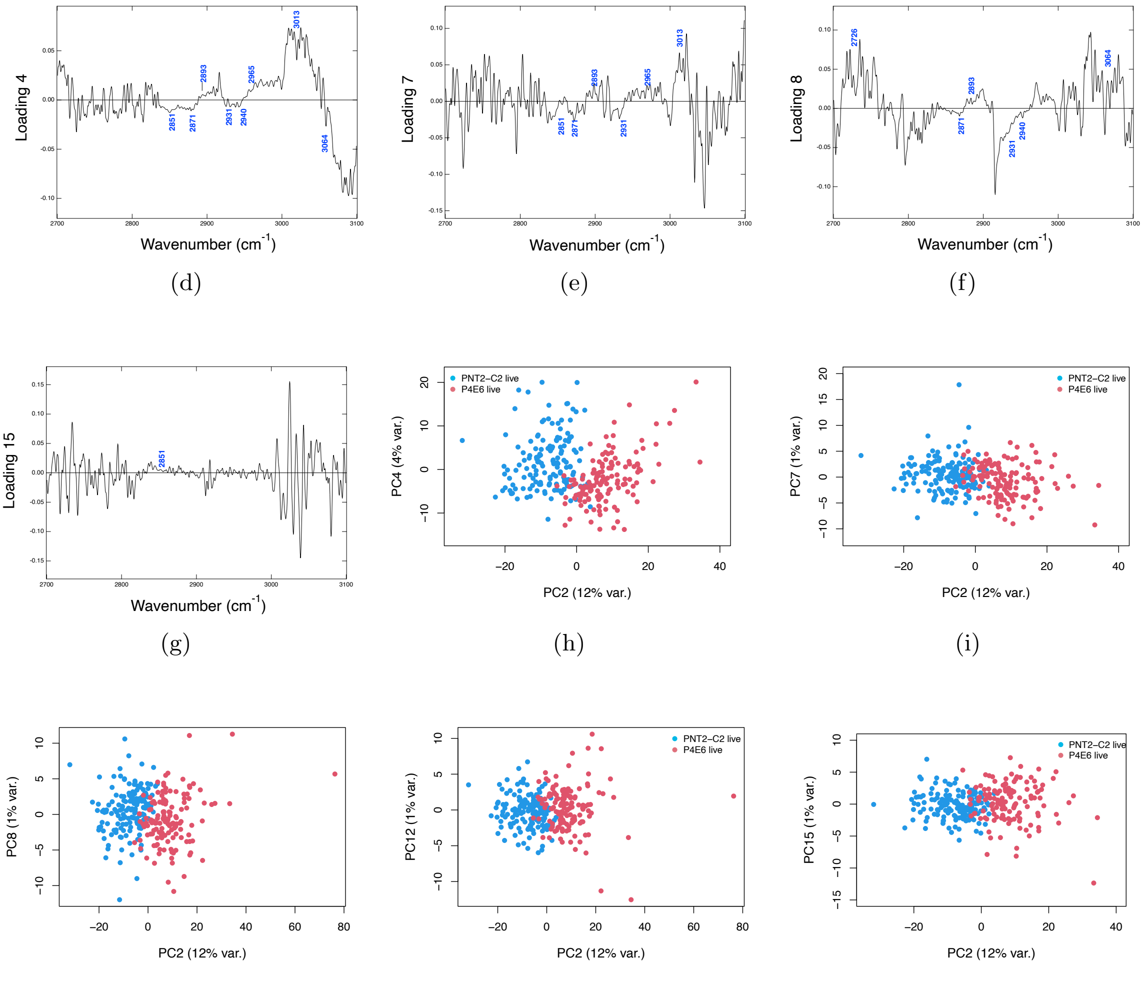

(d) (e) (f)

(g) (h) (i)

(j) (k) (l)

FIG. S10. (a) Table showing the PCs, %-variance captured, PCA group-mean separations, ratio of the between-to-within group variances, and LDF coefficients for the PNT2-C2 live versus P4E6 live comparison in the high-wavenumber region. The table is ordered according to column 4. (b) PCA-LDA histogram results taken over the first 15 contributing PCs as per the table in (a). Converged LOOV = 99.7% separation of classes with 15 PCs (79% variance captured) (cf. Table S4). PCs 2, 4, 7, 8, and 15 with highest-weighted LDF-coefficients contribute maximally to the histogram group-separation. (c), (d), (e), (f), and (g) show the loadings corresponding to these PCs with key wavenumbers labelled. (h), (i), (j), (k) and (l) are PCA plots showing the maximally separated groups.

E. PCA AND PCA-LDA—PNT2-C2 LIVE VS. DRIED (FINGERPRINT)

PC	%-var.	PCA grp. mean sep.	Ratio btw-to-within grp. var.	LDF co-eff
1	30	41.9	4.13	-0.105
2	13	11.8	0.29	0.069
4	4	3.9	0.08	0.066
7	2	1.8	0.04	-0.071
5	3	2.0	0.03	0.048
6	2	1.5	0.02	-0.047
3	7	2.0	0.01	-0.021

(a)

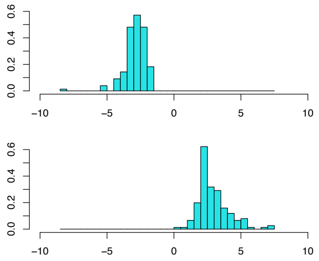

(b)

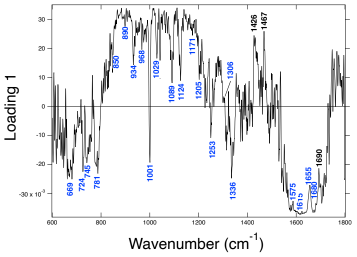

(c)

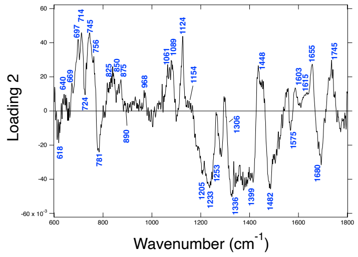

(d)

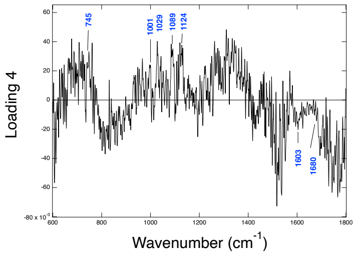

(e)

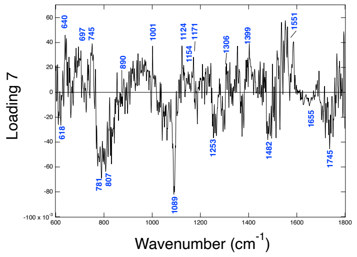

(f)

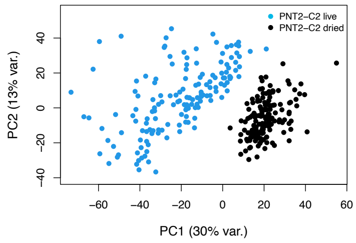

(g)

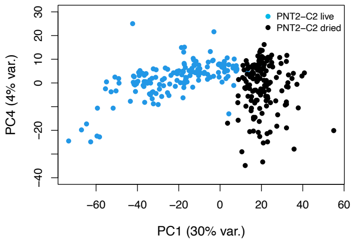

(h)

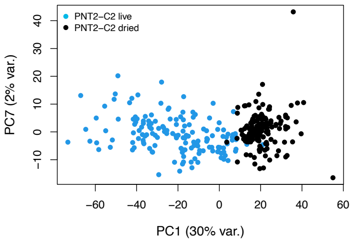

(i)

FIG. S11. (a) Table showing the PCs, %-variance captured, PCA group-mean separations, ratio of the between-to-within group variances, and LDF coefficients for the PNT2-C2 live versus dried comparison in the fingerprint region. The table is ordered according to column 4. (b) PCA-LDA histogram results taken over the first 7 contributing PCs as per the table in (a). Converged LOOV = 99.7% separation of classes with 7 PCs (62% variance captured) (cf. Table S4). PCs 1, 2, 4, and 7 with highest-weighted LDF-coefficients contribute maximally to the histogram group-separation. (c), (d), (e), and (f) show the loadings corresponding to these PCs with key wavenumbers labelled. (g), (h), and (i) are PCA plots showing the maximally separated groups.

E. PCA AND PCA-LDA—PNT2-C2 LIVE VS. DRIED (HIGH WAVENUMBER)

PC	%-var.	PCA grp. mean sep.	Ratio btw-to-within grp. var.	LDF co-eff
2	18	24.4	6.96	-0.303
3	19	5.1	0.15	0.131
4	4	2.6	0.08	-0.141
6	2	1.6	0.08	-0.211
7	1	1.2	0.05	0.213
5	3	0.6	0.01	-0.052
1	41	1.3	0.00	-0.007

(a)

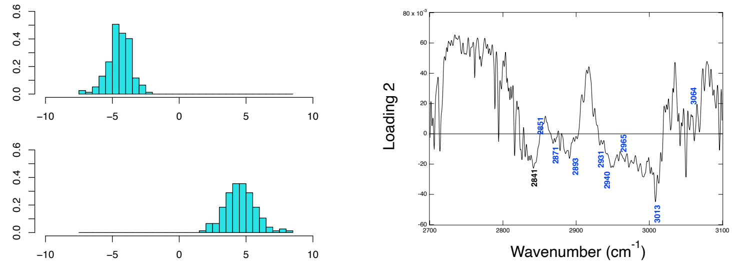

(b) (c)

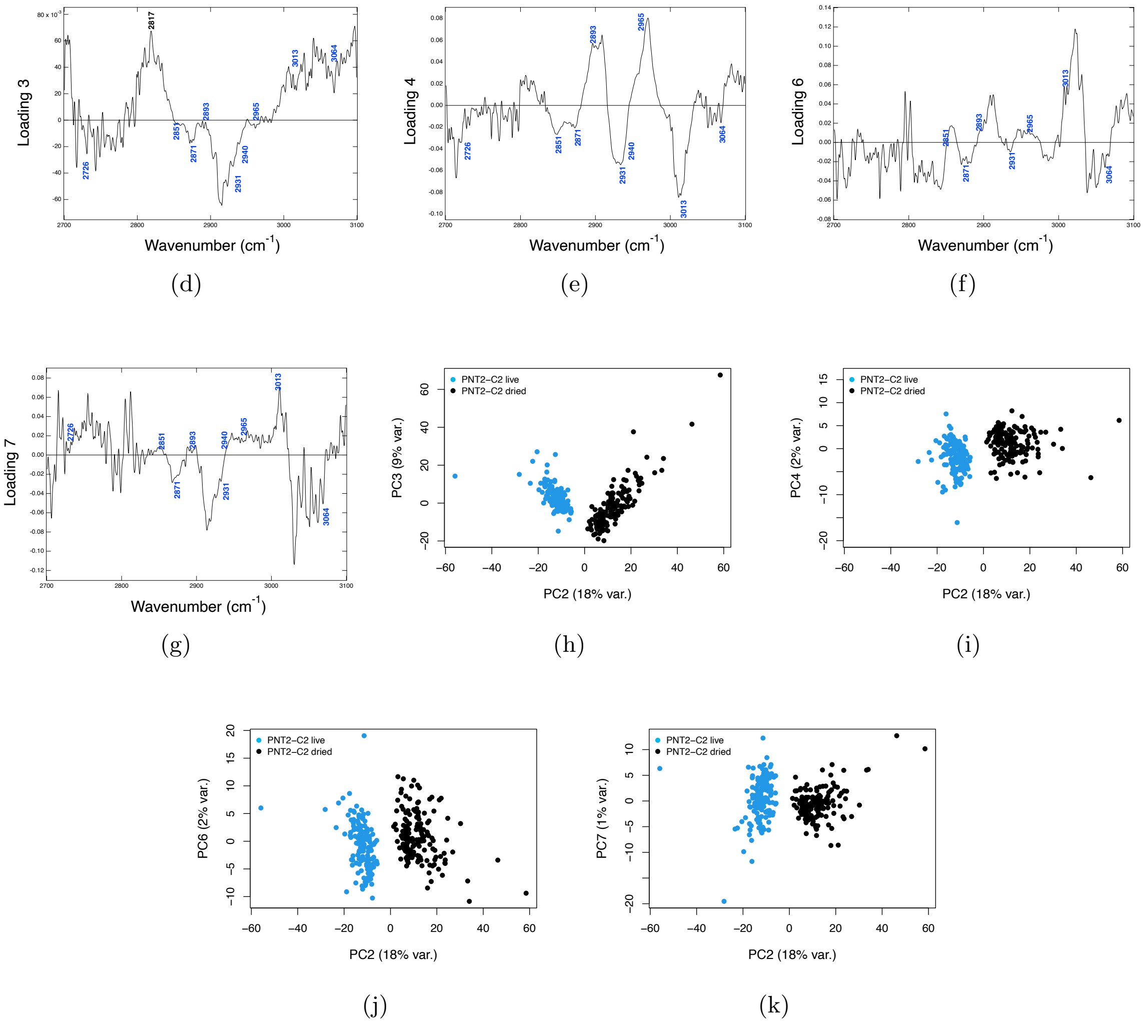

FIG. S12. (a) Table showing the PCs, %-variance captured, PCA group-mean separations, ratio of the between-to-within group variances, and LDF coefficients for the PNT2-C2 live versus dried comparison in the high-wavenumber region. The table is ordered according to column 4. (b) PCA-LDA histogram results taken over the first 7 contributing PCs as per the table in (a). Converged LOOV = 99.8% separation of classes with 7 PCs (77% variance captured) (cf. Table S4). PCs 2, 3, 4, 6, and 7 with highest-weighted LDF-coefficients contribute maximally to the histogram group-separation. (c), (d), (e), (f), and (g) show the loadings corresponding to these PCs with key wavenumbers labelled. (h), (i), (j) and (k) are PCA plots showing the maximally separated groups.

F. PCA AND PCA-LDA—P4E6 LIVE VS. DRIED (FINGERPRINT)

PC	%-var.	PCA grp. mean sep.	Ratio btw-to-within grp. var.	LDF co-eff
1	34	47.0	6.00	-0.123
4	3	5.0	0.19	-0.140
3	6	5.5	0.13	0.085
2	12	4.5	0.04	-0.034
5	2	1.7	0.03	0.075
6	2	0.8	0.01	-0.039

(a)

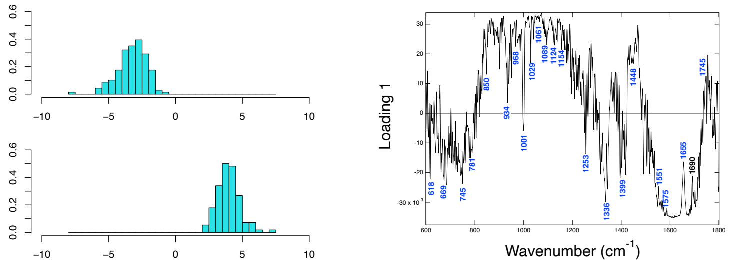

(b) (c)

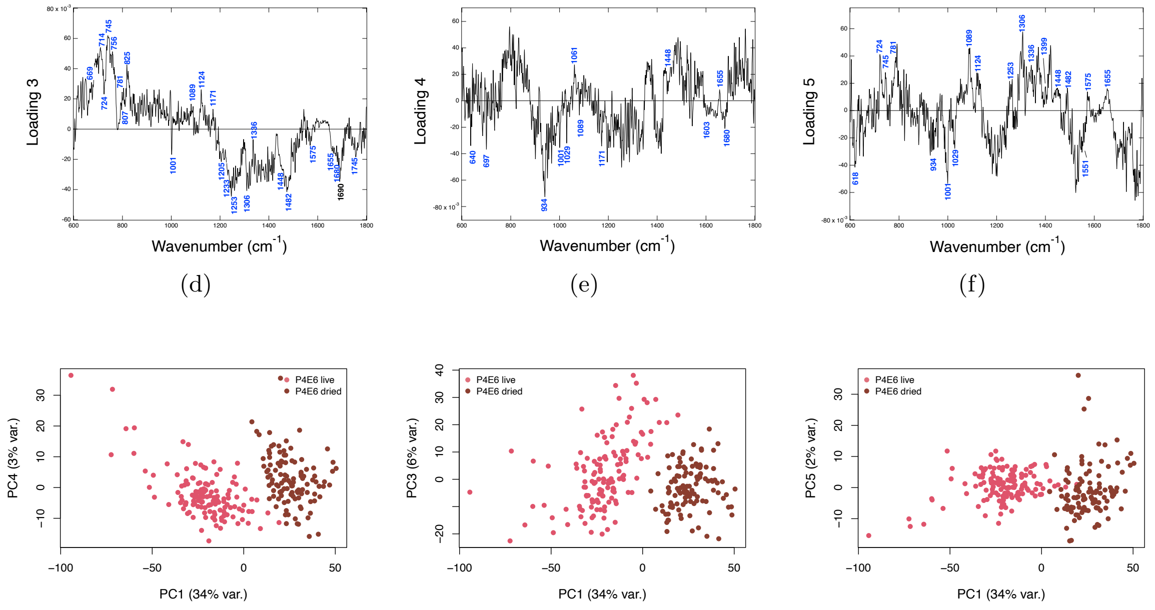

(d) (e) (f)

(g) (h) (i)

FIG. S13. (a) Table showing the PCs, %-variance captured, PCA group-mean separations, ratio of the between-to-within group variances, and LDF coefficients for the P4E6 live versus dried comparison in the fingerprint region. The table is ordered according to column 4. (b) PCA-LDA histogram results taken over the first 6 contributing PCs as per the table in (a). Converged LOOV = 100% separation of classes with 6 PCs (59% variance captured) (cf. Table S4). PCs 1, 3, 4, and 5 with highest-weighted LDF-coefficients contribute maximally to the histogram group-separation. (c), (d), (e), and (f) show the loadings corresponding to these PCs with key wavenumbers labelled. (g), (h), and (i) are PCA plots showing the maximally separated groups.

F. PCA AND PCA-LDA—P4E6 LIVE VS. DRIED (HIGH WAVENUMBER)

PC	%-var.	PCA grp. mean sep.	Ratio btw-to-within grp. var.	LDF co-eff
2	9	11.4	0.99	-0.290
3	6	9.0	0.85	-0.346
1	48	23.8	0.75	0.120
4	3	2.0	0.07	0.164
5	2	1.4	0.04	-0.138
6	0.2	0.6	0.00	-0.026
7	1	0.1	0.00	0.021

(a)

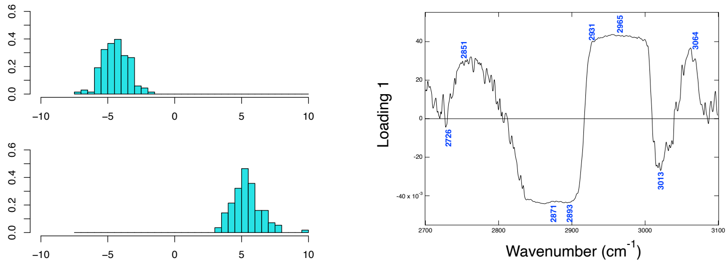

(b) (c)

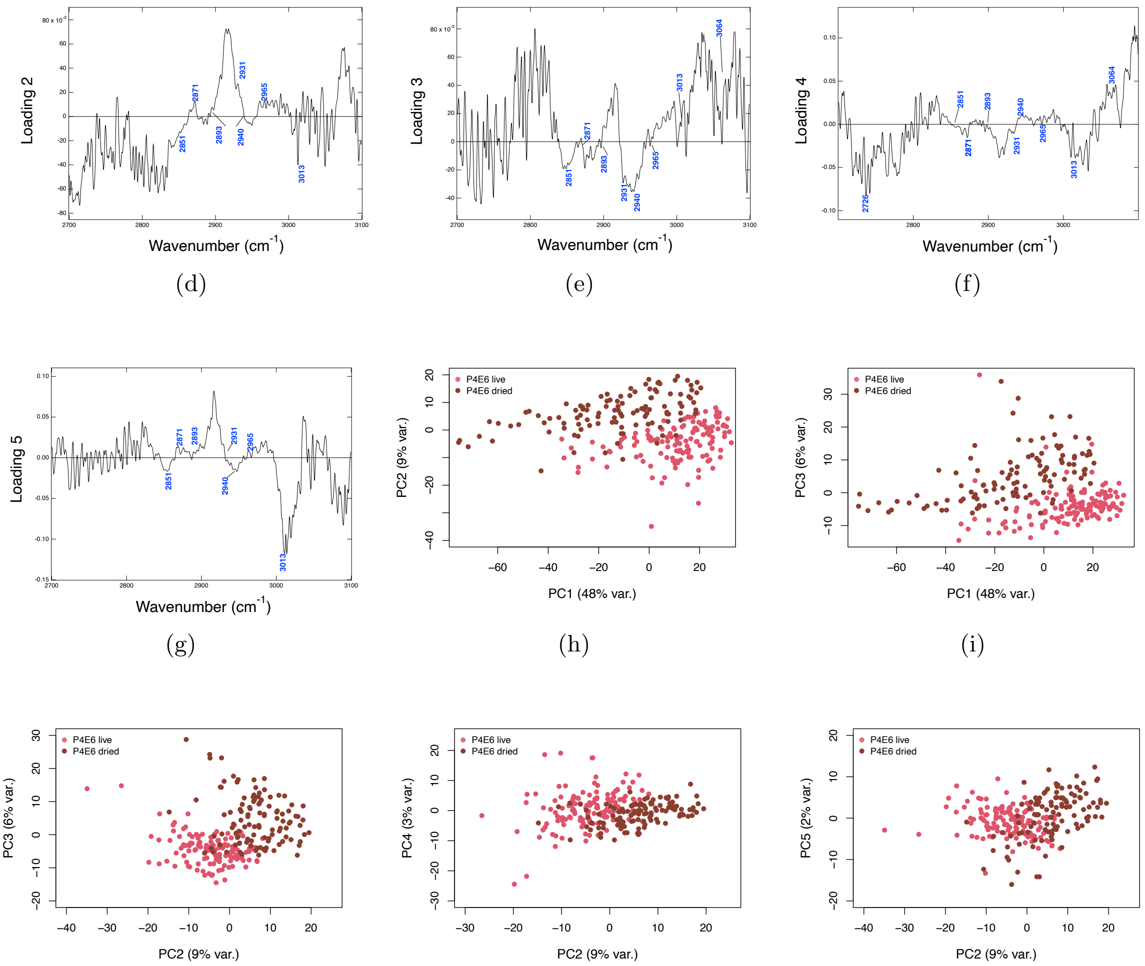

(d) (e) (f)

(g) (h) (i)

(j) (k) (l)

FIG. S14. (a) Table showing the PCs, %-variance captured, PCA group-mean separations, ratio of the between-to-within group variances, and LDF coefficients for the P4E6 live versus dried comparison in the high-wavenumber region. The table is ordered according to column 4. (b) PCA-LDA histogram results taken over the first 7 contributing PCs as per the table in (a). Converged LOOV = 100% separation of classes with 7 PCs (72% variance captured) (cf. Table S4). PCs 1, 2, 3, 4, and 5 with highest-weighted LDF-coefficients contribute maximally to the histogram group-separation. (c), (d), (e), (f), and (g) show the loadings corresponding to these PCs with key wavenumbers labelled. (h), (i), (j), (k), and (l) are PCA plots showing the maximally separated groups.

G. PCA AND PCA-LDA—PNT2-C2 DRIED VS. P4E6 DRIED (FINGERPRINT)

PC	%-var.	PCA grp. mean sep.	Ratio btw-to-within grp. var.	LDF co-eff
2	11	26.3	4.73	0.203
3	6	6.0	0.16	-0.092
4	5	4.4	0.10	0.083
6	3	3.4	0.10	-0.107
5	4	2.8	0.05	-0.063
1	19	6.2	0.04	0.029
9	2	1.3	0.02	0.066
8	2	0.9	0.01	0.041
10	2	0.8	0.01	0.044
7	2	0.5	0.00	-0.019

(a)

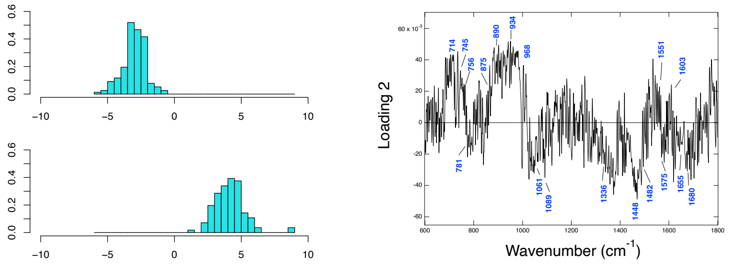

(b) (c)

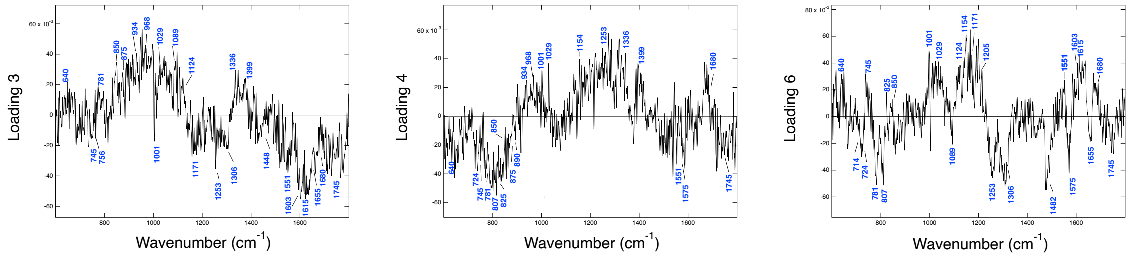

(d) (e) (f)

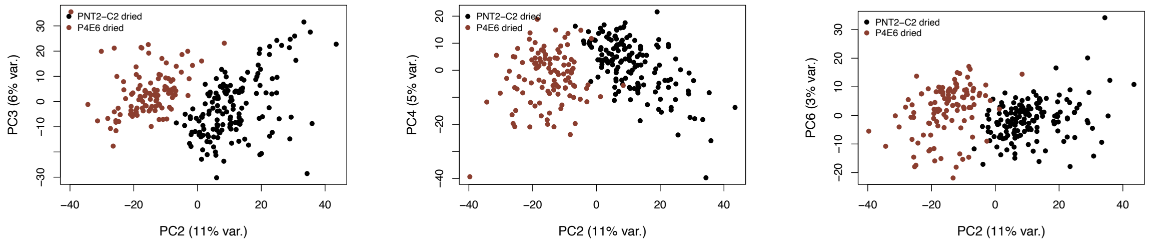

(g) (h) (i)

FIG. S15. (a) Table showing the PCs, %-variance captured, PCA group-mean separations, ratio of the between-to-within group variances, and LDF coefficients for the PNT2-C2 dried versus P4E6 dried comparison in the fingerprint region. The table is ordered according to column 4. (b) PCA-LDA histogram results taken over the first 10 contributing PCs as per the table in (a). Converged LOOV = 100% separation of classes with 10 PCs (54% variance captured) (cf. Table S4). PCs 2, 3, 4, and 6 with highest-weighted LDF-coefficients contribute maximally to the histogram group-separation. (c), (d), (e), and (f) show the loadings corresponding to these PCs with key wavenumbers labelled. (g), (h), and (i) are PCA plots showing the maximally separated groups.

G. PCA AND PCA-LDA—PNT2-C2 DRIED VS. P4E6 DRIED (HIGH WAVENUMBER)

PC	%-var.	PCA grp. mean sep.	Ratio btw-to-within grp. var.	LDF co-eff
3	11	19.3	7.51	0.328
1	47	13.6	0.19	0.054
2	14	3.6	0.05	-0.047
7	1	0.4	0.01	-0.082
4	2	0.7	0.01	-0.049
6	1	0.4	0.01	0.071
5	2	0.1	0.00	0.016

(a)

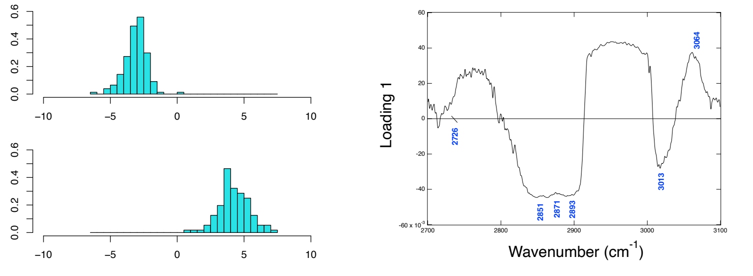

(b) (c)

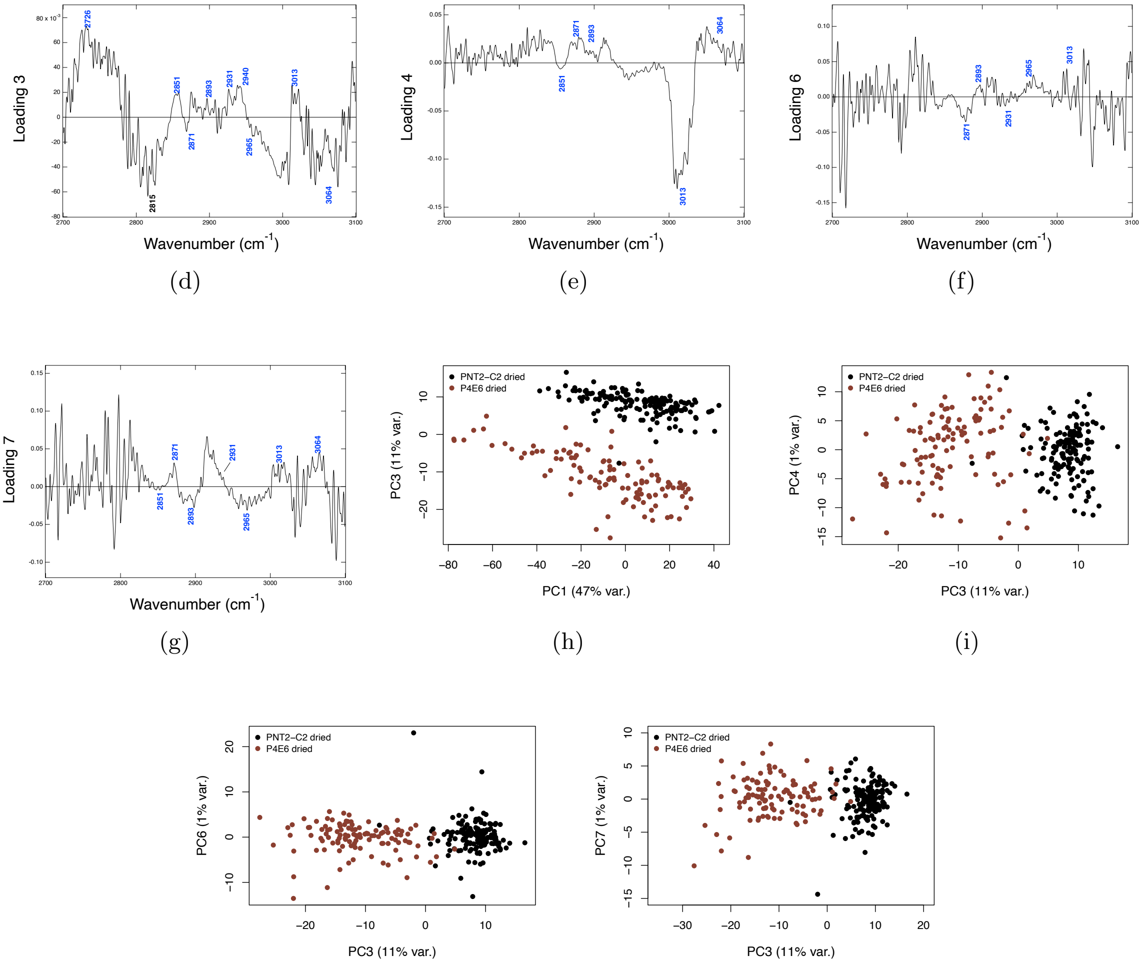

(d) (e) (f) (g) (h) (i) (j) (k)

FIG. S16. (a) Table showing the PCs, %-variance captured, PCA group-mean separations, ratio of the between-to-within group variances, and LDF coefficients for the PNT2-C2 dried versus P4E6 dried comparison in the high-wavenumber region. The table is ordered according to column 4. (b) PCA-LDA histogram results taken over the first 7 contributing PCs as per the table in (a). Converged LOOV = 99% separation of classes with 7 PCs (77% variance captured) (cf. Table S4). PCs 1, 3, 4, 6, and 7 with highest-weighted LDF-coefficients contribute maximally to the histogram group-separation. (c), (d), (e), (f), and (g) show the loadings corresponding to these PCs with key wavenumbers labelled. (h), (i), (j), and (k) are PCA plots showing the maximally separated groups.

H. PCA AND PCA-LDA—WEIGHTED LOADINGS RESULTS

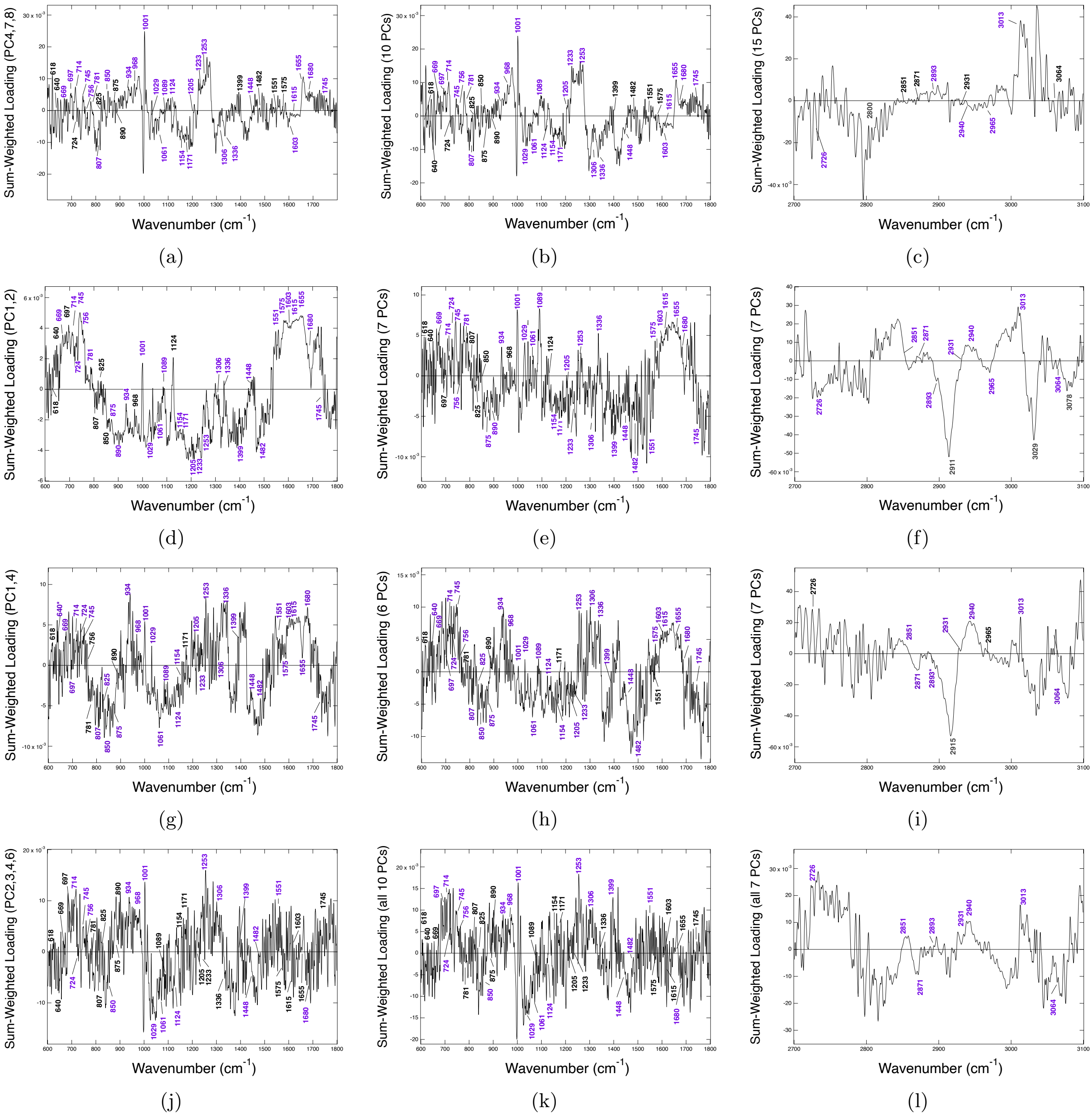

FIG. S17. Weighted-sum plots corresponding to the PCA loadings used to assess the significance of the biomarkers separating the various class comparisons (cf. Tables S9–S16). Purple indicates a marker that is emergent above the noise in the total sum-weighted loadings. Black means that the marker is missing, i.e., either it is zero or significantly within the noise.

I. DIFFERENCE PLOTS

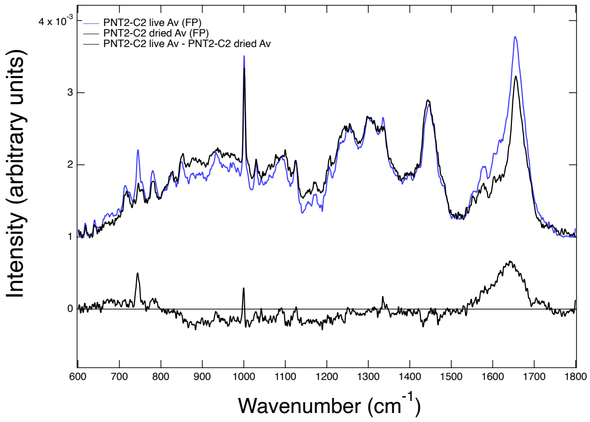

(a) PNT2-C2 live vs. dried (FP)

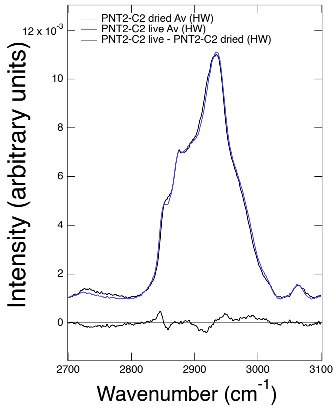

(b) PNT2-C2 live vs. dried (HW)

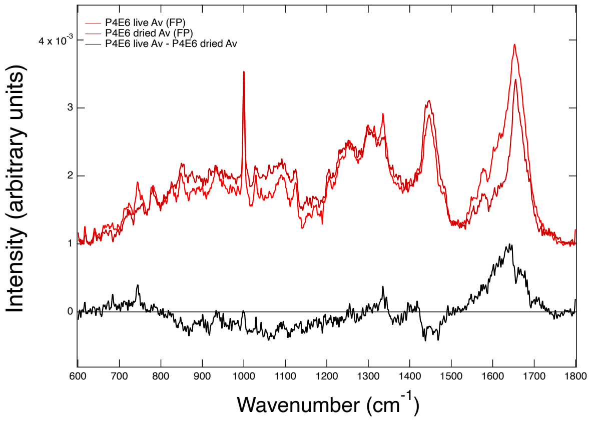

(c) P4E6 live vs. dried (FP)

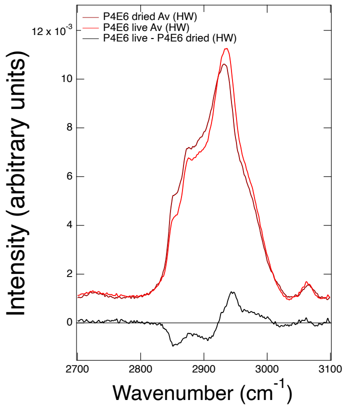

(d) P4E6 live vs. dried (HW)

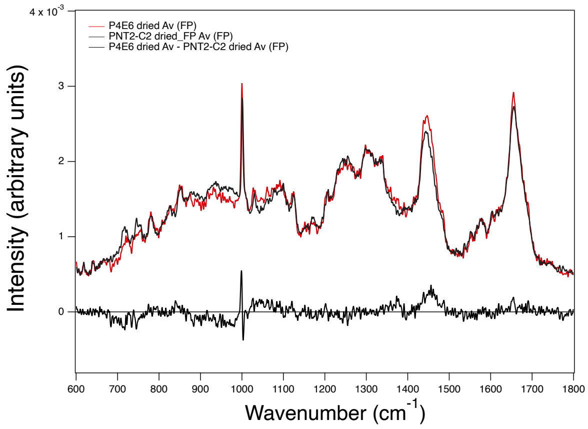

(e) PNT2-C2 vs. P4E6 dried (FP)

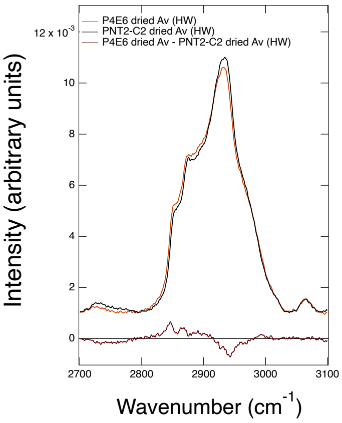

(f) PNT2-C2 vs. P4E6 dried (HW)

FIG. S18. Baselined and normalised spectral averages and spectral difference plots for the statistically converged datasets, comprising: (a) the fingerprint (FP) region, and (b) high-wavenumber (HW) region for PNT2-C2 live and dried, (c) the fingerprint (FP) region, and (d) high-wavenumber (HW) region for P4E6 live and dried, and (e) the fingerprint (FP) region, and (f) high-wavenumber (HW) region for PNT2-C2 and P4E6 dried. See also Fig. 1(a) and (b) for PNT2-C2 and P4E6 live.

J. SUBTRACTED DIFFERENCE PLOTS

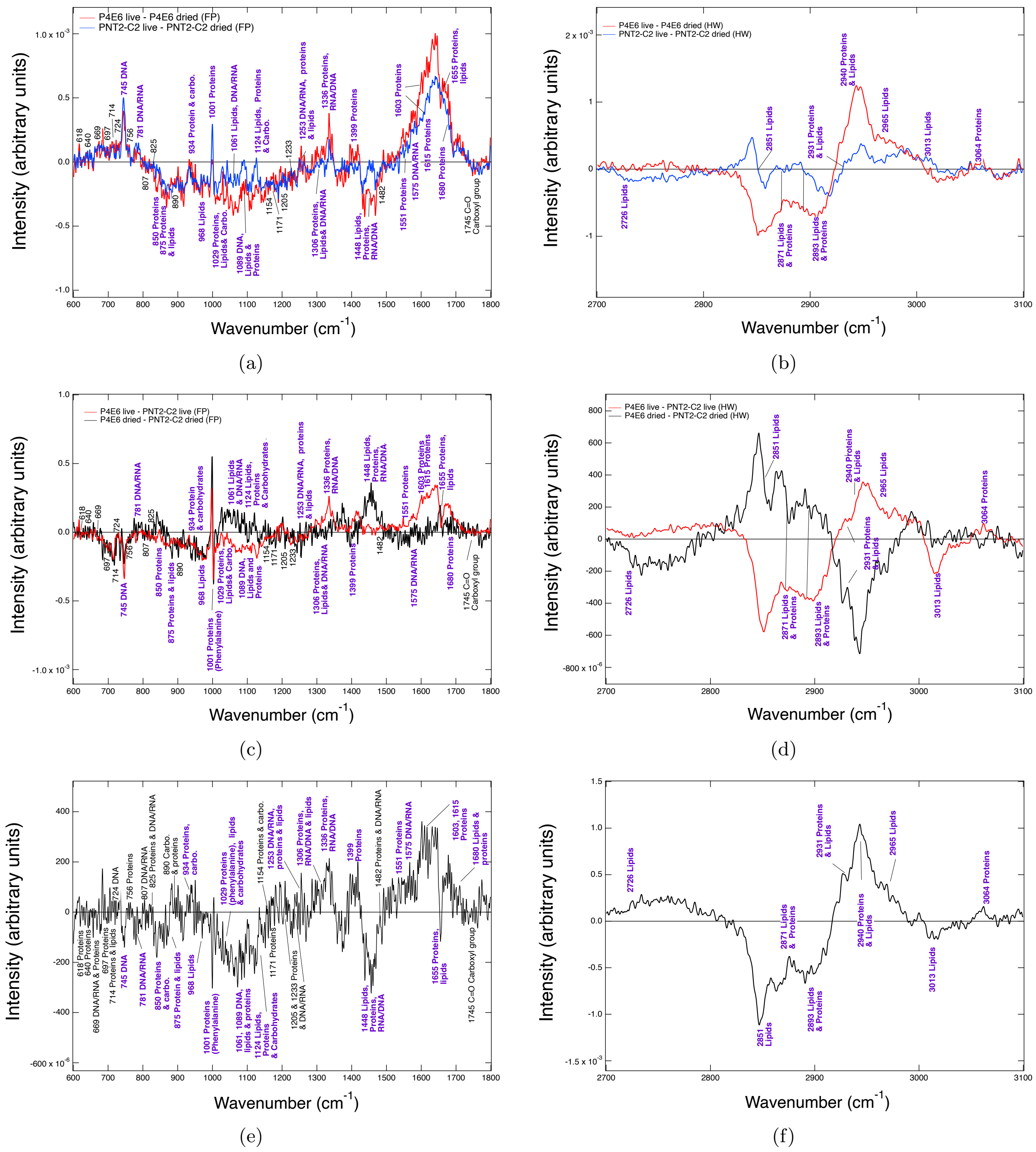

FIG. S19. Baselined and normalised spectral difference-plots inclusive of the spectral background for: (a) fingerprint and (b) high-wavenumber cancer and normal subtractions, (c) fingerprint and (d) high wavenumber live and dried disease-state subtractions, and (e) fingerprint and (f) high wavenumber live disease state minus dried disease state subtractions.
